# Molecular Recognition in an Aqueous Medium Using Water-Soluble Prismarene Hosts

**DOI:** 10.1021/acs.orglett.2c00819

**Published:** 2022-04-07

**Authors:** Rocco Del Regno, Giuseppina D. G. Santonoceta, Paolo Della Sala, Margherita De Rosa, Annunziata Soriente, Carmen Talotta, Aldo Spinella, Placido Neri, Carmelo Sgarlata, Carmine Gaeta

**Affiliations:** † Dipartimento di Chimica e Biologia “A. Zambelli”, Università di Salerno, Via Giovanni Paolo II 132, I-84084 Fisciano, Salerno, Italy; ‡ Dipartimento di Scienze Chimiche, Università degli Studi di Catania, Viale A. Doria 6, I-95125 Catania, Italy

## Abstract

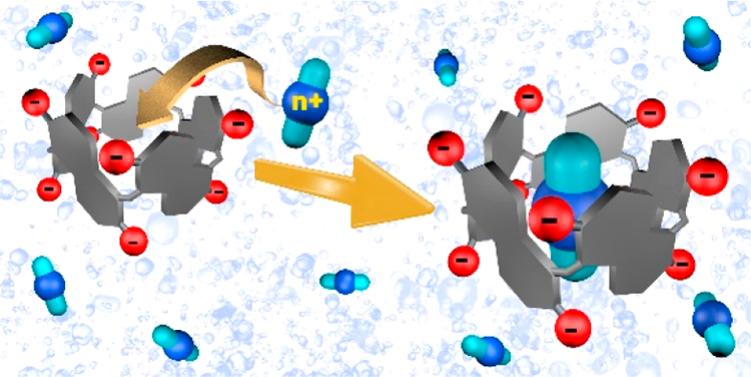

The synthesis of water-soluble prism[*n*]arenes (*n* = 5 and 6) bearing anionic carboxylato groups on the rims is described. The binding properties of this novel class of water-soluble hosts are studied by nuclear magnetic resonance and calorimetry. The complexation of singly and doubly charged ammonium guests with the more rigid pentamer is enthalpically driven by secondary interactions, while the thermodynamic fingerprint for the larger hexamer reveals driving forces that strongly depend on the guest charge and/or size.

Naphthalene-based macrocycles have recently attracted a great deal of attention in supramolecular chemistry, because of their structural and conformational features.^1−5^ In this context, very recently our group reported a new class of deep-cavity macrocycles named prismarenes.^6−9^ Prismarenes are constituted by five or six 1,5-methylene-bridged naphthalene units and are obtained by an acid-catalyzed condensation of 2,6-dialkoxynaphthalene and formaldehyde.^6,8^

Water-soluble hosts^10^ based on classical cyclophane macrocycles such as calixarenes and pillararenes^10,11^ have been widely investigated for their complexation properties in aqueous media. On the contrary, water-soluble naphthalene-based macrocycles have remained largely less studied, and only very recently have Jiang^12^ and co-workers reported the recognition of hydrophilic guests by water-soluble naphthotubes. As is known,^12^ the structural and conformational features of water-soluble hosts play a crucial role in determining their binding affinity in water for charged guests, and therefore, it would be very beneficial to investigate the strength and thermodynamic nature of the forces driving the complexation processes. Thus, we envisioned that the inclusion of charged guests inside the deep hydrophobic cavity of prismarenes could reveal intriguing thermodynamic features closely connected to their structural properties. On the basis of these considerations, in this work, we report the synthesis of novel water-soluble carboxylato-prismarenes **PrS[*n*]**^COO–^ [*n* = 5 and 6 (Scheme 1)] as well as their recognition properties in aqueous solution toward ammonium guests **1–7** (Figure 1) determined by NMR and calorimetric investigation. The synthesis of water-soluble prismarenes **PrS[*n*]**^COO–^ (*n* = 5 and 6) is outlined in Scheme 1.

**Scheme 1:**
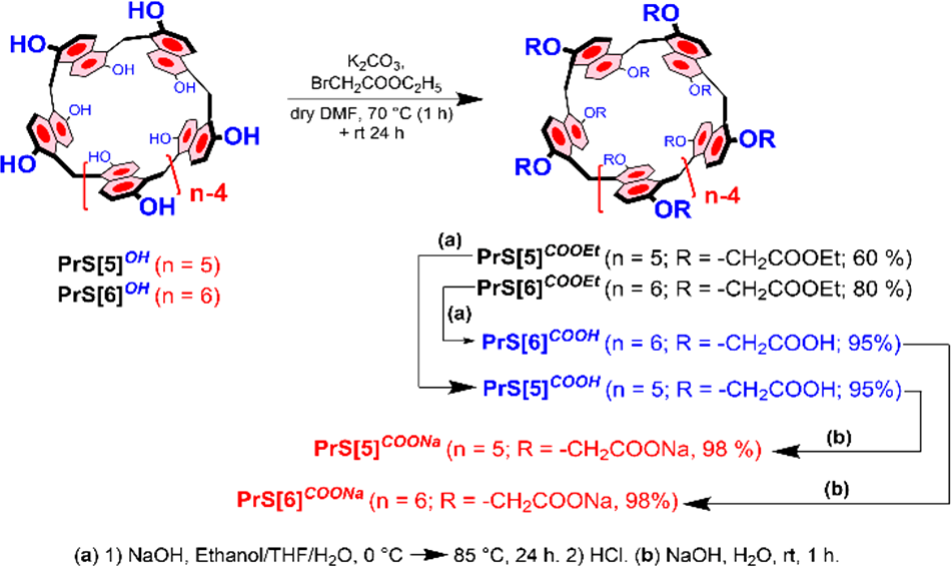
Synthesis of Water-Soluble Prismarenes

**Figure 1:**
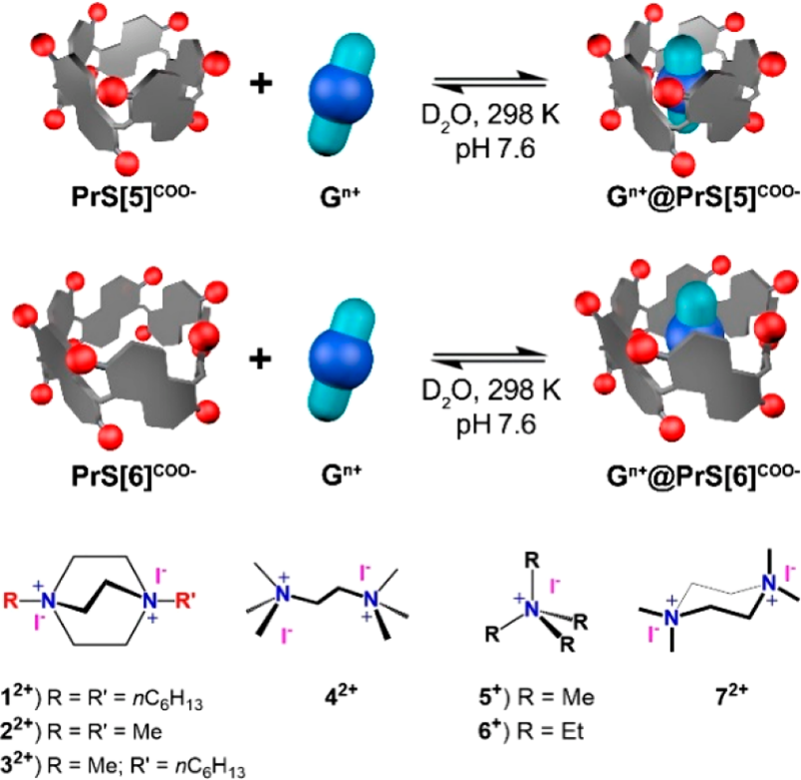
Schematic complexation equilibrium of **PrS[5]**^**COO–**^ (top) and **PrS[6]**^**COO–**^ (bottom) with ammonium guests at 25 °C in a buffered aqueous solution (pH 7.6).

*Per*-hydroxylated prismarenes^9^**PrS[5]**^**OH**^ and **PrS[6]**^**OH**^ were alkylated with ethyl bromoacetate in the presence of K_2_CO_3_ as the base and dry DMF as the solvent. This procedure gave ethoxycarbonylmethoxy-prismarenes **PrS[5]**^**COOEt**^ and **PrS[6]**^**COOEt**^ in 60% and 80% yields, respectively (Scheme 1). The hydrolysis of **PrS[5]**^**COOEt**^ and **PrS[6]**^**COOEt**^ in the presence of NaOH afforded carboxylic acid-substituted prismarenes **PrS[5]**^**COOH**^ and **PrS[6]**^**COOH**^, respectively, in 95% yield. ^1^H NMR spectra of carboxylic prismarenes **PrS[5]**^**COOH**^ and **PrS[6]**^**COOH**^ at 298 K are in agreement with their *D*_5_ and *D*_6_ symmetry, respectively, observed also for the parent **PrS[*n*]**^**COOEt**^.

Finally, neutralization of **PrS[5]**^**COOH**^ and **PrS[6]**^**COOH**^ by treatment with an aqueous NaOH solution afforded **PrS[5]**^**COONa**^ and **PrS[6]**^**COONa**^ salts, respectively, which exhibited good solubility in aqueous medium. In agreement with the *D*_5_ symmetry of the molecule, the ^1^H NMR spectrum of **PrS[5]**^**COO–**^ in a D_2_O solution (Figure 2c, pH 7.6, phosphate buffer) at 298 K showed the presence of an AX system at 7.89 and 6.88 ppm (Figure 2c; *J* = 8.0 Hz; Δδ = 1.01 ppm), attributable to the aromatic H atoms of the macrocycle, while at 4.74 ppm, a singlet was detected, attributable to the methylene bridges. Finally, OCH_2_ groups resonated as an AB system at 4.25 and 4.14 ppm.

**Figure 2:**
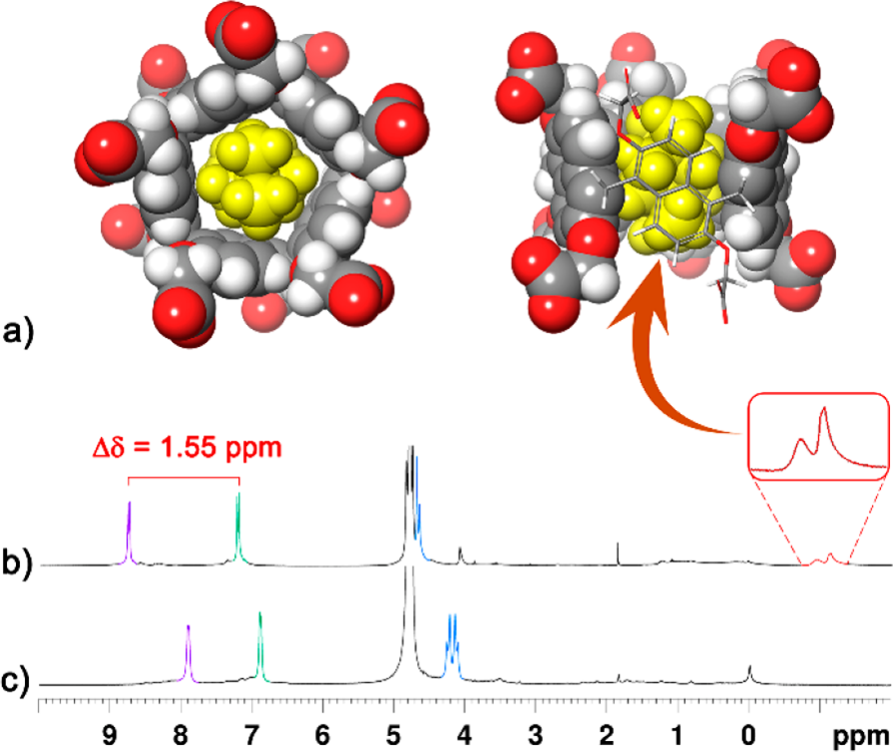
(a) Different views of the DFT-optimized structure of the **7**^2+^@**PrS[5]**^**COO–**^ complex at the B97D3/SVP/SVPFIT level of theory. (b and c) ^1^H NMR spectra (400 MHz, buffered D_2_O solution, pH 7.60, 298 K) of **PrS[5]**^**COO–**^ and of an equimolar solution of **PrS[5]**^**COO–**^ and **7**^2+^·2I^–^, respectively.

The addition of ammonium cations **1–7** as iodide salts to a D_2_O solution (pH 7.6, phosphate buffer) of **PrS[5]**^**COO–**^ gave substantial changes in its ^1^H NMR spectrum (Supporting Information), indicative of the formation of *endo*-cavity complexes (Figures 1 and 2 and the Supporting Information), in slow exchange on the NMR time scale. In detail, when *N,N,N′,N′*-tetramethylpiperazonium salt **7**^2+^·(I^–^)_2_ and **PrS[5]**^**COO–**^ were mixed in an equimolar ratio in D_2_O (Figure 2b,c), the formation of the **7**^2+^@**PrS[5]**^**COO–**^*endo*-cavity complex was confirmed by the presence of upfield-shifted H atom signals of **7**^2+^ to negative values of chemical shift (Figure 2b,c and the Supporting Information). Interestingly, the Δδ value between the aromatic doublets of **PrS[5]**^**COO–**^ increased from 1.01 ppm for the free host to 1.55 ppm [Δδ = 8.80 ppm – 7.25 ppm = 1.55 ppm (Figure 2b)] upon inclusion of **7**^2+^ inside the cavity of the carboxylato-prism[5]arene (Figure 2a). Diagnostic cross-peaks were present in the NOESY spectrum of the **7**^2+^@**PrS[5]**^**COO–**^ complex between the shielded methylene protons of **7**^2+^ at negative values of chemical shift and ArH protons of the host **PrS[5]**^**COO–**^. These results are strongly indicative of the inclusion of the piperazonium cation inside the cavity of the host.^6,8^ Analogously, upfield-shifted ^1^H NMR signals were observed upon complexation of ammonium guests **1–6** inside the cavity of the **PrS[5]**^**COO–**^ host [Figure 1].

The DFT-optimized structure of the **7**^2+^@**PrS[5]**^**COO–**^ complex (Figure 2a and the Supporting Information) at the B97D3/SVP/SVPFIT level of theory reveals that the **PrS[5]**^**COO–**^ host adopts a conformation in which the aromatic walls assume canting angle^13^ values of ∼90° and therefore define a regular pentagonal prism. The piperazonium cation (yellow in Figure 2a) is accommodated inside the internal cavity of **PrS[5]**^**COO–**^ and establishes several (approximately nine) C–H···π interactions with an average C–H···π centroid distance of 2.6 Å.^14^

ITC calorimetric studies^15^ confirm that **PrS[5]**^**COO**–****^ forms 1:1 complexes with ammonium guests **1–7** (Figure 1). In fact, the macrocycle cavity of **PrS[5]**^**COO**–****^ is unable to encapsulate more than one guest while the negatively charged rims prevent the possible assembly of capsular structures^16,17^ due to the strong electrostatic repulsion between the two facing **PrS[5]**^**COO**–****^ molecules.

The inclusion complexes exhibit affinity constant values ranging from 10^3^ to 10^5^ M^–1^, and the results in Table 1 and Figure 3 suggest that the stability of the **G**^*n*+^@**PrS[5]**^**COO–**^ complexes is significantly affected by the structural features of the differently charged **G**^*n*+^ guests.

**Table 1:** Log *K* Values and Thermodynamic Parameters^a^ for Host–Guest Complex Formation at 298 K in an Aqueous Solution (pH 7.6, 70 mM phosphate buffer)

	**PrS[5]^COONa^**			**PrS[6]^COONa^**		
guest	log *K*	Δ*H*^0^^b^	Δ*S*^0^^c^	log *K*	Δ*H*^0^^b^	Δ*S*^0^^c^
**5**^+^	4.51 (9)	–15.21 (2)	36 (2)	3.5 (2)	–1.33 (6)	63 (4)
**6**^+^	3.50 (3)	–24.66 (1)	–15.8 (6)	3.3 (2)	–1.54 (6)	58 (4)
**4**^2+^	5.1 (2)	–26.45 (2)	9 (3)	3.10 (5)	–7.95 (2)	32.7 (9)
**1**^2+^	4.5 (1)	–30.02 (2)	–14 (3)	3.41 (6)	–12.21 (2)	25 (2)
**3**^2+^	4.5 (1)	–33.65 (3)	–27 (3)	3.29 (1)	–12.63 (1)	20.5 (4)
**2**^2+^	3.87 (6)	–18.53 (2)	13 (2)	2.91 (3)	–14.92 (1)	5.7 (6)
**7**^2+^	5.3 (2)	–33.91 (3)	–12 (4)	3.05 (3)	–16.71 (1)	2.3 (6)

a σ in parentheses.

b Expressed in kilojoules per mole.

c Expressed in joules per degree per mole.

**Figure 3:**
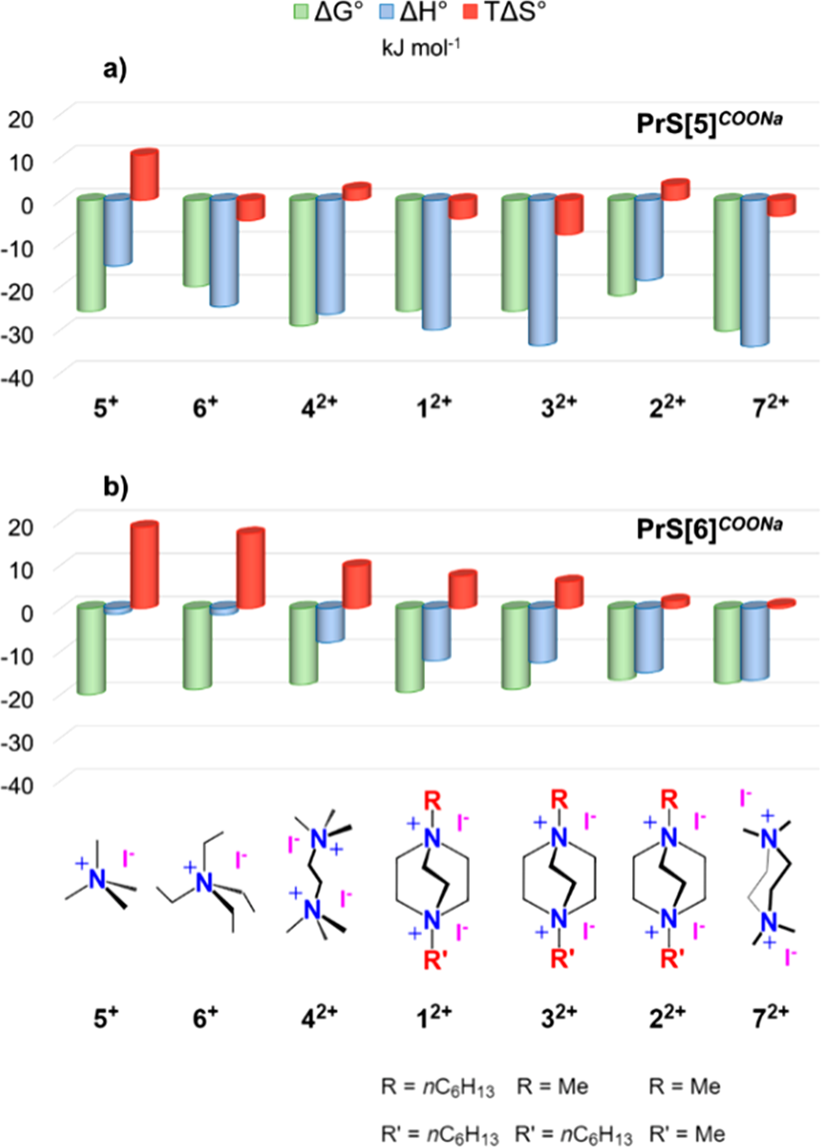
Thermodynamic parameters for host–guest complex formation of (a) **PrS[5]**^**COO–**^ and (b) **PrS[6]**^**COO–**^ with positively charged guests at 25 °C in a buffered aqueous solution (pH 7.6).

The largest binding affinities were achieved for the complexation of doubly charged cations **7**^2+^ and **4**^2+^, with log *K* values of 5.3 (2) and 5.1 (2), respectively. With regard to the complexation of singly charged ammonium guests, **PrS[5]**^**COO–**^ displays a greater affinity for tetramethylammonium **5**^+^ than the larger tetraethylammonium **6**^+^ with a **5**^+^/**6**^+^ selectivity ratio of 9.5. The larger binding affinity of **PrS[5]**^**COO–**^ for dicationic than for monocationic ammonium guests suggests that Coulombic favorable COO^–^/N^+^ interactions play a crucial role in an efficient complexation, along with cation−π interactions. 1,4-Dimethyl-DABCO **2**^2+^ has the smallest affinity for **PrS[5]**^**COO–**^, while the presence of longer *n*-hexyl chains in 1,4-dihexyl-DABCO **1**^2+^ and 1-hexyl-4-methyl-DABCO **3**^2+^ increases the stability of their respective complexes by ∼0.6 log unit. The binding parameters (Figure 3a) highlighted that the inclusion of all ammonium cations in the hydrophobic cavity of **PrS[5]**^**COO–**^ is due to enthalpically favorable attractive forces (including electrostatic, cation−π, and CH−π interactions) that drive the recognition process. The sizable enthalpy gain [Δ*H*^0^ ranges from −15.21 to −33.91 kJ mol^–1^ (see Figure 3)] overrides the cost in energy needed for the desolvation of all of the interacting components. The complexations of piperazonium **7**^2+^ and DABCOHeMe **3**^2+^ are more exothermic (Figure 3a) than those of other guests but with an entropic penalty that accounts for the loss of degrees of freedom due to host–guest complex formation and/or to a lower level of desolvation upon inclusion in the aromatic cavity of the host. Desolvation is still a relevant process for the binding of ^+^NMe-based cations such as **2**^2+^ and **4**^2+^ and, interestingly, for tetramethylammonium guest **5**^+^, which all benefit from a favorable entropic contribution (see Figure 3a). Significantly, the *endo*-cavity complexes of doubly charged guests such as **7**^2+^, **2**^2+^, **1**^2+^, **3**^2+^, and **4**^2+^ with **PrS[5]**^**COO–**^, which show large enthalpic gains (Table 1), have packing coefficients (PC) of 57%, 63%, 65%, 66%, and 56%, respectively, in the optimal 55 ± 9% PC range as reported by Rebek and Mecozzi.^18^

NMR and calorimetry measurements indicated that **PrS[6]**^**COO–**^ also forms only 1:1 inclusion complexes with all of the charged guests despite its larger cavity. Also in this case, upon addition of cations **1–7** as iodide salts to a D_2_O solution (pH 7.6, phosphate buffer) of **PrS[6]**^**COO–**^, a new set of upfield-shifted signals emerged in its ^1^H NMR spectrum (Supporting Information) due to the formation of complexes slowly exchanging on the NMR time scale.

The association constants (Table 1 and Figure 3) determined for the formation of the **G**^*n*+^@**PrS[6]**^**COO–**^ complexes (Figures 1) are all on the order of 10^3^ M^–1^ and lower than the values found for the analogous **G**^*n*+^@**PrS[5]**^**COO–**^ complexes. This difference in affinity could be attributed to the lower **G**^*n*+^/**PrS[6]**^**COO–**^ complementarity, due to the larger size of the hexamer cavity that causes a scarce steric fit with **G**^*n*+^ in Figure 1. Although the binding free energies for all of the **G**^2+^@**PrS[6]**^**COO–**^ complexes are comparable (approximately −18 kJ mol^–1^), intriguing differences can be revealed when the enthalpic and entropic contributions are determined. The inclusion of doubly charged guests **G**^2^^+^ inside the **PrS[6]**^**COO–**^ cavity is an enthalpically driven process occurring by electrostatic interactions between the carboxylate and cationic groups, along with multiple cation−π and CH−π interactions with the π-electron rich **PrS[6]**^**COO–**^ cavity (Figure 3b). Interestingly, enthalpically favored **G**^2+^@**PrS[6]**^**COO–**^ complexes have packing coefficients (PC) of 63% (**1**^2+^), 48% (**2**^2+^), 60% (**3**^2+^), and 40% (**4**^2+^), within the optimal 55 ± 9% PC range.^18^ However, the structural features of doubly charged guests **G**^2^^+^ seem to strongly influence the enthalpic term (Δ*H*^0^ gain follows the order **7**^2+^ > **2**^2+^ > **3**^2+^ > **1**^2+^ > **4**^2+^). In fact, in the case of DABCO cations **1**^2+^–**3**^2+^, longer *n*-hexyl chains in **1**^2+^ confer larger hydrophobicity and a smaller charge to radius ratio, resulting in a lower overall charge density. Consequently, for the formation of the **1**^2+^@**PrS[6]**^**COO–**^ complex, electrostatic and/or cation−π interactions are less efficient than for the other DABCO cations **2**^2+^ and **3**^2+^.^19^ Similar binding affinities of **PrS[6]**^**COO–**^ were measured for the complexation of singly charged cations **5**^+^ and **6**^+^, with log *K* values of 3.5 (2) and 3.3 (2), respectively.

The *K* value of 1990 M^–1^ (log *K* = 3.3) measured by ITC for the formation of the **6**^+^@**PrS[6]**^**COO–**^ complex was confirmed by integration of the slowly exchanging ^1^H NMR signals for the free and complexed guest [*K* = 1815 M^–1^ (Figure S32)]. The inclusion of monocations **5**^+^ and **6**^+^ in the **PrS[6]**^**COO–**^ cavity is an entropically favored and driven process (*T*Δ*S*^0^ ∼ 18 kJ mol^–1^ on average). Desolvation of the **5**^+^ and **6**^+^ cations, which are strongly hydrated in their free state, as well as the release of water molecules from the solvent-filled cavity of **PrS[6]**^**COO–**^ to the bulk of the solvent upon complexation account for a favorable increase in entropy. Moreover, the less suitable fit between these smaller monocationic guests and the larger host cavity, which results in greater prismarene walls flexibility, further contributes to the large entropic gain observed. As one can see from Figure 3b, moving from doubly charged guests to singly charged cations **5**^+^ and **6**^+^, the decrease in the Δ*H*^0^ contribution is compensated by an increased *T*Δ*S*^0^ term. This result indicates, for the *endo*-cavity complexation of **PrS[6]**^**COO–**^ with charged guests, an enthalpy–entropy compensation effect (EEC), which is commonly observed in supramolecular recognition systems.^20^ As reported in the literature,^20^ EEC plots (see the Supporting Information) permit one to gain insight into complexation thermodynamic fingerprints. In detail, the slope and the intercept (*T*Δ*S*_0_^0^) of the plot of *T*Δ*S*^0^ versus Δ*H*^0^ (Supporting Information) might provide relevant information for changes in the conformation and desolvation of the host and guest.^20^ Generally, slope values of <1 might be expected for macrocycles characterized by a more rigid structure in which secondary host–guest interactions prevail. A slope closer to unity could be indicative of a rearrangement of the hydrogen-bond network^21^ and conformational changes in the macrocycle structure. In agreement with these observations, the complexation of **PrS[6]**^**COO–**^ exhibits a slope of 1.12 (7) and a *T*Δ*S*_0_^0^ value of 19.6 (8) kJ mol^–1^, which both account for greater host flexibility and a larger number of water molecules in the interior and exterior of the hexamer **PrS[6]**^**COO–**^ cavity than in the case of the pentamer. In fact, the complexation thermodynamic fingerprint for **PrS[5]**^**COO–**^ shows a lower enthalpy–entropy compensation effect; a slope of 0.7 (1) and an intercept of 19 (3) kJ mol^−1^ can be attributed to the higher conformational rigidity of the pentamer. In agreement with these deductions, **G**^*n*+^@**PrS[5]**^**COO–**^ complexes show packing coefficients in the optimal 55 ± 9% PC range, indicative of a good steric fit between size and shape of the cations **G**^*n*+^ and the narrower cavity of the pentamer.

In conclusion, here are described the synthesis of novel water-soluble carboxylato-prism[*n*]arenes (*n* = 5 and 6) and their *endo*-cavity complexation properties toward organic ammonium cations **G**^*n*+^@**PrS[*n*]**^**COO–**^ (*n* = 5 and 6) in water. Calorimetry studies showed that the complexation processes are driven by thermodynamic factors depending on prismarene size and cation charge and shape. The inclusion of ammonium cations **1–7** in the cavity of **PrS[5]**^**COO–**^ is driven by enthalpically favorable attractive forces. The larger **PrS[6]**^**COO–**^ hexamer forms *endo*-cavity complexes with association constants lower than those found for **PrS[5]**^**COO–**^. *Endo*-cavity inclusion of a singly charged ammonium guest by carboxylato-prism[6]arene is entropically favored; desolvation of the cations and the release of water molecules from the solvent-filled cavity of the hexamer account for the large entropic gain. On the contrary, the encapsulation of doubly charged guests inside the cavity of **PrS[6]**^**COO–**^ is enthalpically driven mostly due to electrostatic, cation−π, and CH−π interactions.

The synthesis of water-soluble prismarenes, described here for the first time, and the thermodynamic fingerprints of their complexations in aqueous medium could pave the way for the construction of new prismarene-based supramolecular systems with intriguing properties.

## Supporting Information Available

Detailed synthetic procedures, one- and two-dimensional NMR spectra, HR mass spectra, details of DFT calculations details, and details of ITC titrations (PDF)
