# Supplementary material for: Molecular Recognition in an Aqueous Medium Using Water-Soluble Prismarene Hosts
Source: Org Lett. 2022 Apr 7;24(14):2711–5. doi: 10.1021/acs.orglett.2c00819 (PMC9016763; doi:10.1021/acs.orglett.2c00819)
Supplement: Supplementary file 1 — ol2c00819_si_001.pdf [file ol2c00819_si_001.pdf]

Supplementary Information

Molecular Recognition in Aqueous Medium

Using Water Soluble Prismarene Hosts

Rocco Del Regno,[a,†] Giuseppina D. G. Santonoceta, [b,†] Paolo Della Sala,[a] Margherita De Rosa,[a] Annunziata Soriente,[a] Carmen Talotta,[a] Aldo Spinella,[a] Placido Neri,[a] Carmelo Sgarlata[b,*] and Carmine Gaeta[a,*]

[a.] Laboratory of Supramolecular Chemistry (SupraLab@UniSa), Dipartimento di Chimica e Biologia "A. Zambelli", Università di Salerno, Via Giovanni Paolo II 132, I-84984, Fisciano, Italy.

[b.] Dipartimento di Scienze Chimiche, Università degli Studi di Catania, Viale A. Doria 6, I-95125 Catania, Italy.

[†] R.D.R. and G.D.G.S. contributed equally to this work.

**Table of Contents**	**Pages**
General Experimental Details	**S2**
Procedures for the Synthesis of the Carboxylato-Prismarenes	**S3-S5**
Copies of 1D, 2D NMR and HR Mass Spectra	**S6-S27**
1D NMR Titration Experiments for the Formation of the $\mathbf{G^{n+}@PrS[\mathit{n}]^{COO-}}$ Complexes	**S28-S37**
ITC Titrations Experiments	**S38-S53**
DFT optimized structure of the $\mathbf{7^{2+}@\ PrS[5]^{\mathit{COO-}}}$ complex	**S54-S59**
2D NOESY of 7^{2+}@PrS[5]$^{COO-}$	**S60**
References	**S61**

General Experimental Details

HR MALDI mass spectra were recorded using a Bruker Solaris XR Fourier transform ion cyclotron resonance mass spectrometer equipped with a 7T refrigerated actively shielded superconducting magnet. All samples were recorded in MALDI (4 laser shots were used for each scan) and they were prepared by mixing 10 μL of analyte in dichloromethane or methanol (1 mg/mL) with 10 μL of solution of 2,5-dihydroxybenzoic acid (10 mg/mL in Methanol). The mass spectra were calibrated externally, and a linear calibration was applied. Dichloromethane was dried by heating under reflux over calcium hydride. Tetrahydrofuran was distilled over sodium wire in the presence of benzophenone as indicator[1] while DMF were dried over activated 3 Å molecular sieves[2]. All chemical reagents grade was used without further purification and were used as purchased by Merck, TCI and Fluorochem. Reaction temperatures were measured externally. Reactions were monitored by Merck TLC silica gel plates (0.25 mm) and visualized by UV light 254 nm, or by spraying with H_2SO_4-$Ce(SO_4)_2$. NMR spectra were recorded on a Bruker Avance-600 [600 (^{1}H) and 150 MHz (^{13}C)], Avance-400 [400 (^{1}H) and 100 MHz (^{13}C)], Avance-300 MHz [300 (^{1}H) and 75 MHz (^{13}C)] or Avance-250 MHz [250 (^{1}H) and 62.5 MHz (^{13}C)] spectrometers. Chemical shifts are reported relative to the residual solvent peak[3]. Standard pulse programs, provided by the manufacturer, were used for 2D COSY (cosygpqf), 2D HSQC (hsqcedetgpsisp2.2) and 2D NOESY (noesygpphpp) experiments.
Structural assignments were made with additional information from gCOSY, gHSQC, and geNOESY experiments.

The derivatives **PrS[5]^{OH}** and **PrS[6]^{OH}** were synthesized according to literature procedures.[4]

Procedures for the Synthesis of the Carboxylato-Prismarenes

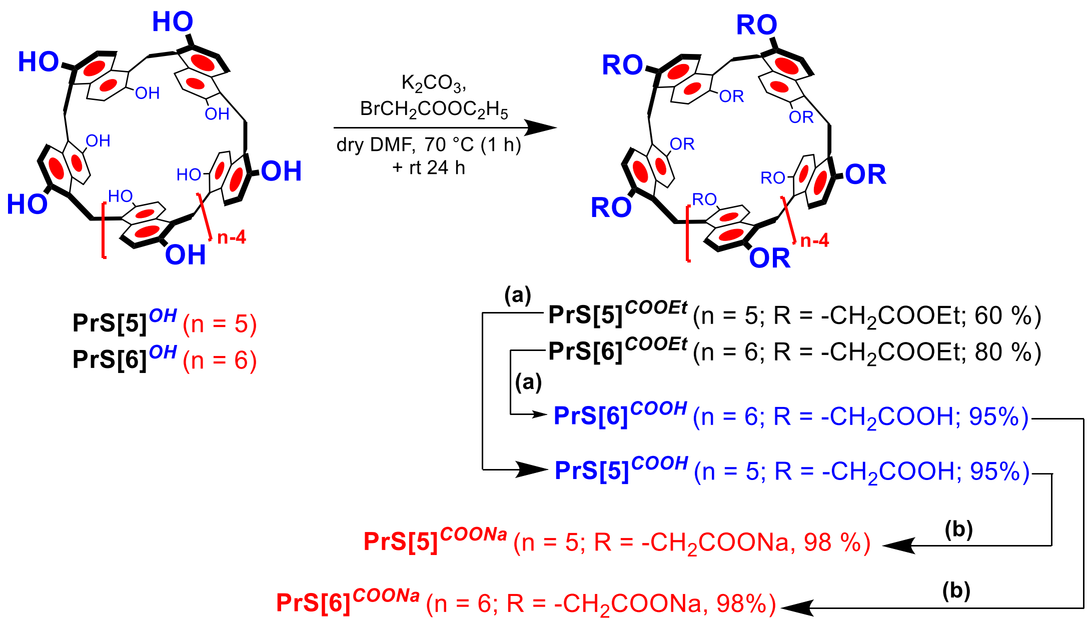

(**a**) 1) NaOH, Ethanol/THF/H_2O, 0 °C → 85 °C, 24 h. 2) HCl. (**b**) NaOH, H_2O, rt, 1 h.

Ethoxycarbonylmethoxy-Substituted Prism[5]arene: Under a nitrogen atmosphere **PrS[5]*^{OH}*** (0.40 g, 0.46 mmol) and potassium carbonate (2.57 g, 18.58 mmol) were dissolved in dry DMF (46 mL). The reaction mixture was stirred at 70 °C (oil bath temperature) for 1 h. Then, the reaction was reported to room temperature and an excess of ethyl bromoacetate (1.55 mL, 13.94 mmol) was added. The mixture was stirred at room temperature for 24 h. The reaction was stopped by adding a 10 % aqueous solution of NH_4Cl (50 mL). After removal of the solvent, the resulting solid was triturated with water (60 mL) and then, the precipitate was filtered. The solid was washed with water (2 x 30 mL) and methanol (2 x 30 mL). The crude product was subject to chromatography on silica gel (CH_2Cl_2/ MeOH = 99:1, v/v) to give the pure product **PrS[5]*^{COOEt}*** (0.48 g, 60 %) as a white solid.

Carboxylic Acid Groups-Substituted Prism[5]arene. PrS[5]*^{COOEt}* (0.40 g, 0.23 mmol), and NaOH (0.70 g, 17.40 mmol), in THF (34 mL), H_2O (34 mL) and Ethanol (34 mL) was stirred at 85 °C (oil bath temperature) for 24 h. After removal of the solvent, 100 mL of ethyl acetate and 50 mL of 1 N HCl solution were added at 0 °C. The aqueous layer was washed with ethyl acetate (5 x 50 mL). Then, the organic layer was washed with brine (50 mL), and it was dried with Na_2SO_4 and concentrated to give the derivative **PrS[5]*^{COOH}*** (0.31 g, 95 %) as a white solid.

Synthesis of Carboxylato-Prism[5]arene. To a suspension of **PrS[5]*^{COOH}*** (0.30 g, 0.21 mmol) in water (15 mL) was added an aqueous solution of NaOH (0.1 g, 2.10 mmol in 3 mL). The reaction mixture was stirred for 1 h at room temperature. The solution was evaporated and triturated with 20 mL of methanol to give the derivative **PrS[5]*^{COONa}*** as a white solid (0.34 g, 98 %).

Ethoxycarbonylmethoxy-Substituted Prism[6]arene: Under a nitrogen atmosphere **PrS[6]*OH*** (0.370 g, 0.36 mmol) and potassium carbonate (2.38 g, 17.19 mmol) were dissolved in DMF (36 mL). The reaction mixture was stirred at 70 °C (oil bath temperature) for 1 h. Then, the reaction was reported to room temperature and an excess of ethyl bromoacetate (1.44 mL, 12.89 mmol) was added. The mixture was stirred at room temperature for 24 h. The reaction was stopped by adding a 10 % aqueous solution of NH_4Cl (50 mL). After removal of the solvent, the resulting solid was triturated with water (60 mL) and then, the precipitate was filtered. The solid was washed with water (2 x 30 mL) and methanol (2 x 30 mL). The crude product was purified through chromatographic column on silica gel (CH_2Cl_2/ MeOH = 98:2, v/v) to give the pure product **PrS[6]*COOEt*** (0.59 g, 80 %) as a brown solid.

Carboxylic Acid Groups-Substituted Prism[6]arene: PrS[6]*COOEt* (0.40 g, 0.19 mmol), and NaOH (0.70 g, 17.42 mmol), in THF (28 mL), H_2O (28 mL) and Ethanol (28 mL), was stirred at 85 °C (oil bath temperature) for 24 h. After removal of the solvent, 100 mL of ethyl acetate and 50 mL of aqueous 1 N solution of HCl were added at 0 °C. The aqueous layer was washed with ethyl acetate (5 x 50 mL). Then, the organic layer was washed with brine (50 mL), and it was dried with Na_2SO_4 and concentrated to give the derivative **PrS[6]*COOH*** (0.32 g, 95 %) as a brown solid.

Synthesis of Carboxylic Groups-Substituted Prism[6]arene: To a suspension of **PrS[6]*COOH*** (0.30, 0.17 mmol) in water (15 mL) was added an aqueous solution of NaOH (0.1 g, 2.10 mmol in 3 mL). The reaction mixture was stirred for 1 h at room temperature. The solution was evaporated and triturated with 20 mL of methanol to give the derivative **PrS[6]*COONa*** as a white solid quantitatively (0.33 g, 98 %).

Derivative PrS[5]*COOEt*:

Mp:.> 300 - 302 °C dec. ^{1}H NMR (CD_2Cl_2, 600 MHz, 298 K): δ 8.02 (*d*, 10H, Ar-*H*, J = 9.6 Hz), 6.84 (*d*, 10H, Ar-*H*, J = 9.6 Hz), 4.83 (*s*, 10H, ArC*H*$_2$Ar), 4.37 and 4.31 (AB, 20H, OC*H*$_2$, J = 16.5 Hz), 4.07-3.96 (m, 20H, OC*H*$_2$CH$_3$), 1.05 (*t*, 30H, OCH$_2$C*H*$_3$, J = 7.2 Hz). ^{13}C{^{1}H} NMR (CD_2Cl_2, 150 MHz, 298 K): δ 169.7, 152.0, 130.1, 125.7, 124.3, 114.6, 67.1, 61.4, 22.2, 14.2. HRMS (FT-ICR MALDI) *m/z* $[M]^+$ calcd for $C_{95}H_{100}O_{30}$: 1720.6299; found: 1720.6300.

Derivative PrS[5]*COOH*:

Mp:.> 309 - 311 °C dec. ^{1}H NMR (CD_3OD, 400 MHz, 298 K): δ 8.02 (*d*, 10H, Ar-*H*, J = 9.6 Hz), 6.91 (*d*, 10H, Ar-*H*, J = 9.6 Hz), 4.83 (overlapped to signal of H_2O, 10H, ArC*H*$_2$Ar), 4.35 and 4.20 (AB, 20H, OC*H*$_2$, J = 16.4 Hz). ^{13}C{^{1}H} NMR (CD_3OD, 100 MHz, 298 K): δ 173.4, 153.1, 130.9, 126.5, 125.2, 115.6, 67.6, 22.7. HRMS (FT-ICR MALDI) m/z $[M]^+$ calcd for $C_{75}H_{60}O_{30}$: 1440.3169; found: 1440.3179.

Derivative PrS[5]*COONa*:

Mp:.> 325 - 327 °C dec. ^{1}H NMR (D_2O, 400 MHz, 298 K): δ 7.89 (*d*, 10H, Ar-*H*, J = 8.0 Hz), 6.88 (*d*, 10H, Ar-*H*, J = 8.8 Hz), 4.74 (overlapped to H_2O signal, 10H, ArC*H*$_2$Ar), 4.21 and 4.14 (AB, 20H, OC*H*$_2$, J = 15.4 Hz). ^{13}C{^{1}H} NMR (D_2O, 100 MHz, 298 K): δ 177.9, 160.6, 151.8, 129.2, 125.1, 123.4, 114.6, 68.3, 21.8. Anal. Calcd for $C_{75}H_{50}Na_{10}O_{30}$: C, 54.23; H, 3.03; Na, 13.84; O, 28.89. Found: C, 54.33; H, 3.13.

Derivative PrS[6]^{COOEt}:

Mp:.> 302 - 304 °C dec. ^{1}H NMR (CD_2Cl_2, 400 MHz, 298 K): δ 8.04 (br, 12H, Ar-H), 6.94 (br, 12H, Ar-H), 4.82-4.67(overlapped, 36 H, ArCH_2Ar and OC*H*$_2$), 4.21 (broad, 24H, OC*H*$_2$CH$_3$), 1.23 (broad, 36H, OCH$_2$C*H*$_3$). ^{13}C{^{1}H} NMR (CD_2Cl_2, 100 MHz, 298 K): δ 169.6, 151.4, 130.4, 125.2, 125.0, 115.0, 67.1, 61.5, 22.1, 14.4. HRMS (FT-ICR MALDI) *m*/*z* $[M]^+$ calcd for $C_{114}H_{120}O_{36}$: 2064.7559; found: 2064.7565.

Derivative PrS[6]^{COOH}:

Mp:.> 311 - 313 °C dec. ^{1}H NMR (DMSO-*d*$_6$, 300 MHz, 393 K): δ 7.98 (*d*, 12H, Ar-*H*, *J* = 9.0 Hz), 7.05 (*d*, 12H, Ar-*H*, *J* = 9.7 Hz), 4.80-4.69 (overlapped, 36H, ArC*H*$_2$Ar and OC*H*$_2$). ^{13}C{^{1}H} NMR (DMSO-*d*$_6$, 75 MHz, 393 K): δ 169.5, 150.5, 129.1, 123.9, 123.4, 114.3, 66.0, 21.0. HRMS (FT-ICR MALDI) m/z $[M]^+$ calcd for $C_{90}H_{72}O_{36}$: 1728.3803; found: 1728.3813.

Derivative PrS[6]^{COONa}:

Mp:.> 320 – 322 °C dec. ^{1}H NMR (D_2O, 250 MHz, 333 K): δ 8.42 (*d*, 12H, Ar-*H*, *J* = 6.5 Hz), 7.43 (*d*, 12H, Ar-*H*, *J* = 8.5 Hz), 5.19 (*s*, 12H, ArC*H*$_2$Ar), 4.59 (overlapped to signal of H_2O, 24H, OC*H*$_2$). ^{13}C{^{1}H} NMR (D_2O, 75 MHz, 333 K): δ 177.0, 152.0, 129.4, 125.0, 124.1, 116.6, 69.4, 22.3. Anal. Calcd for $C_{90}H_{60}Na_{12}O_{36}$: C, 54.23; H, 3.03; Na, 13.84; O, 28.89. Found: C, 54.34; H, 3.12.

Copies of 1D, 2D NMR and HR Mass Spectra

PrS[5]^{COOEt}

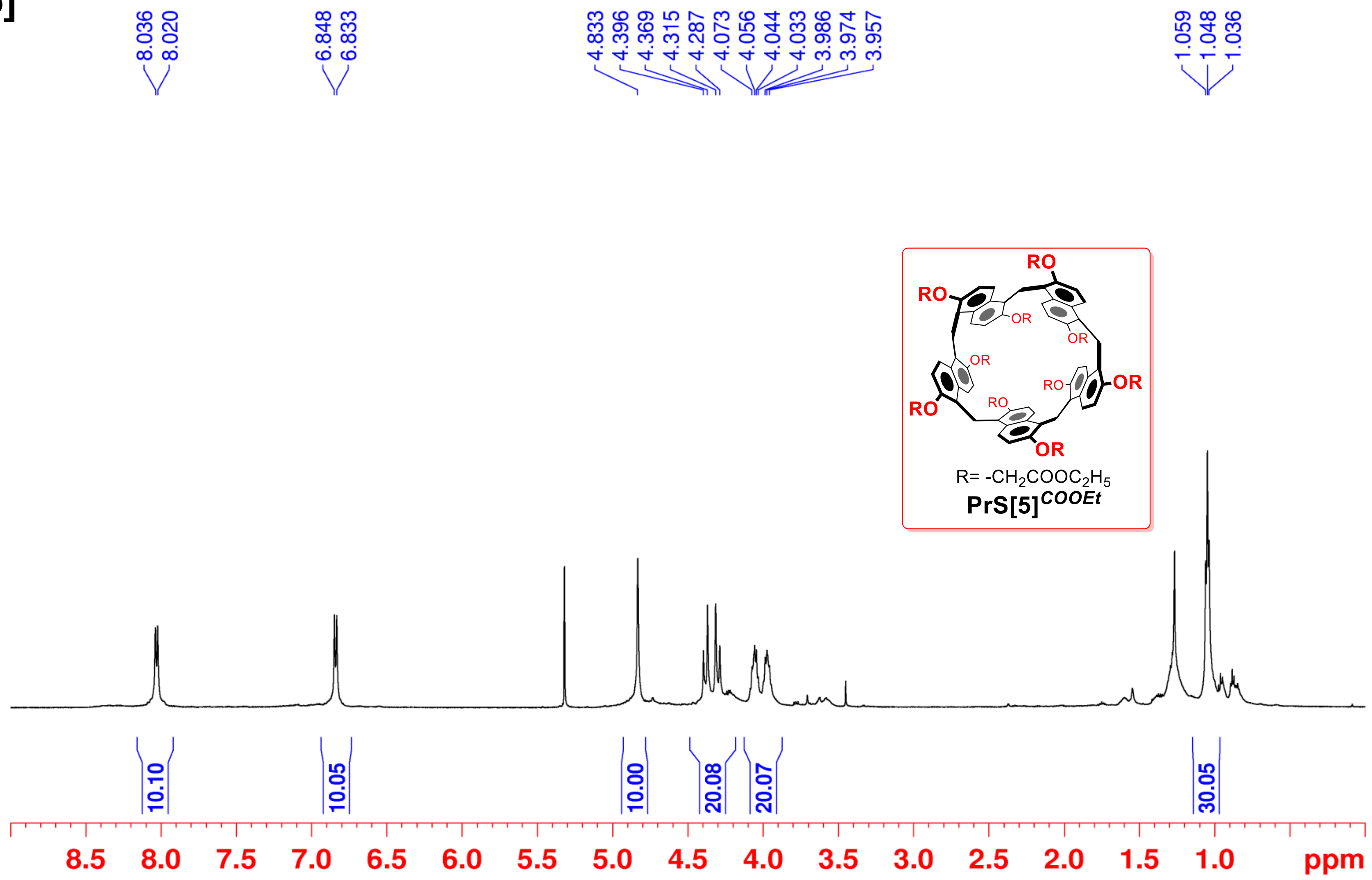

Figure S1: ^{1}H NMR spectrum of **PrS[5]^{COOEt}** (CD_2Cl_2, 600 MHz, 298 K).

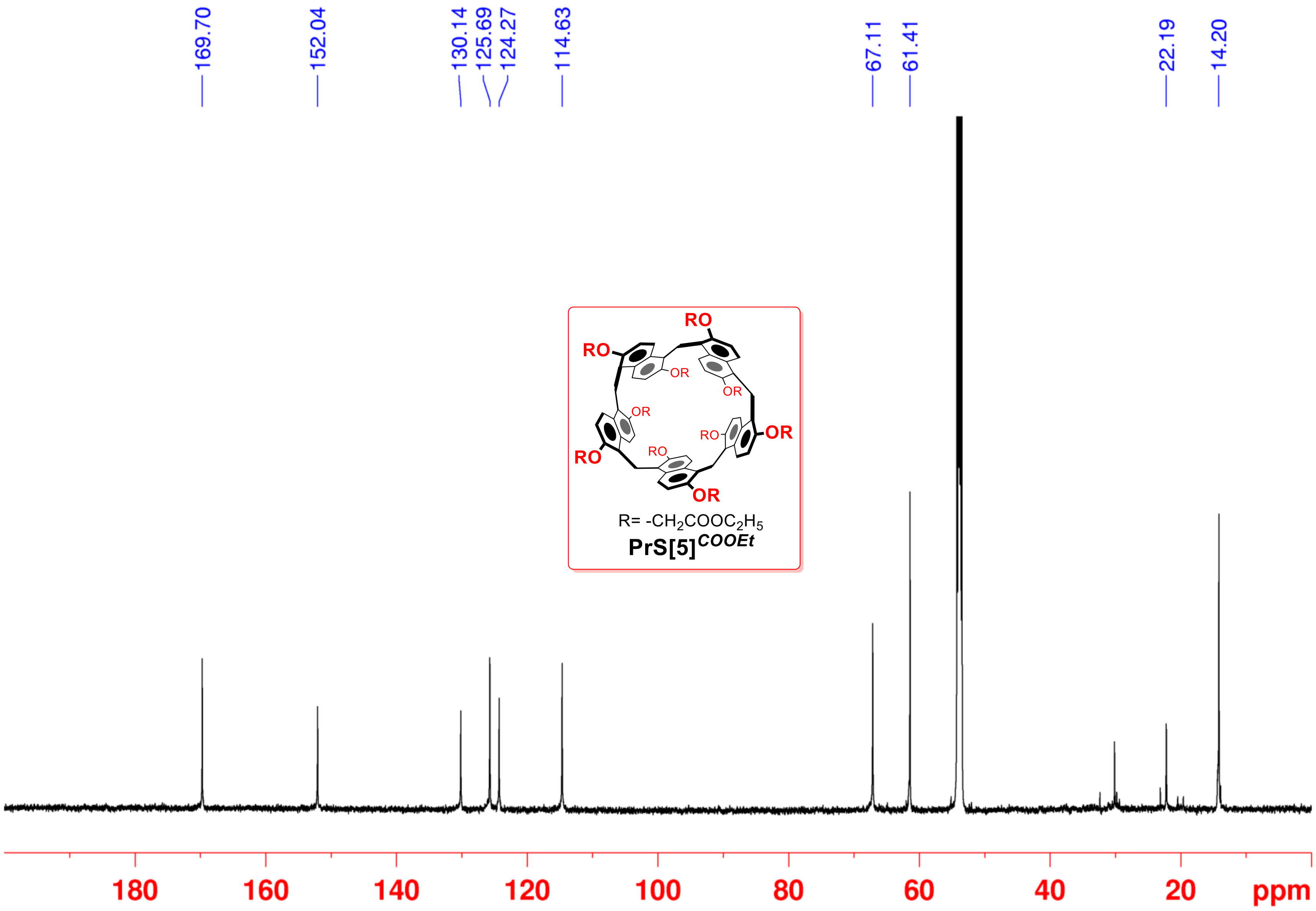

Figure S2: $^{13}C\{^{1}H\}$ NMR spectrum of **PrS[5]*COOEt*** (CD_2Cl_2, 150 MHz, 298 K).

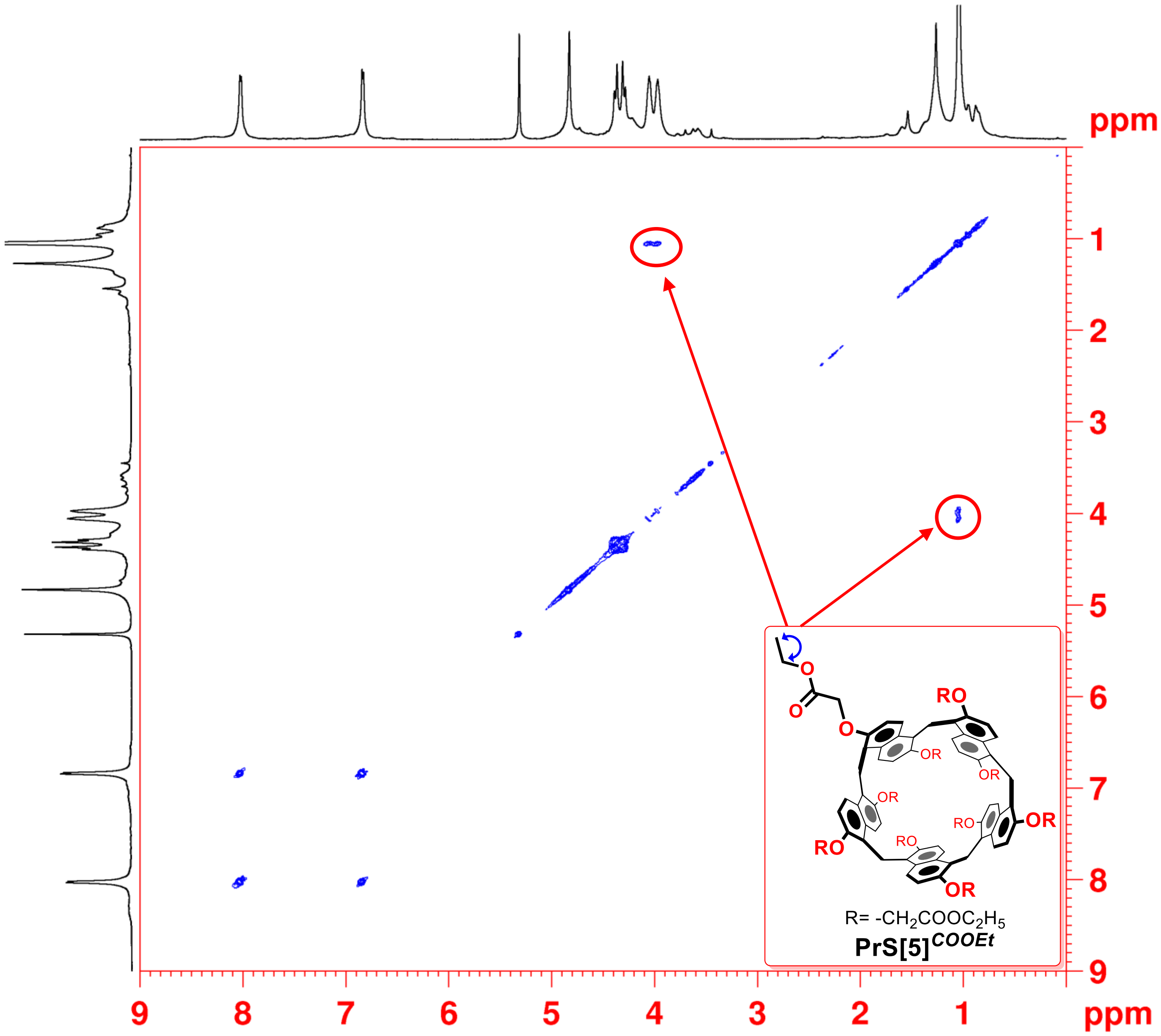

Figure S3: 2D-DQF COSY spectrum of **PrS[5]**^{COOEt} (CD_2Cl_2, 400 MHz, 298 K).

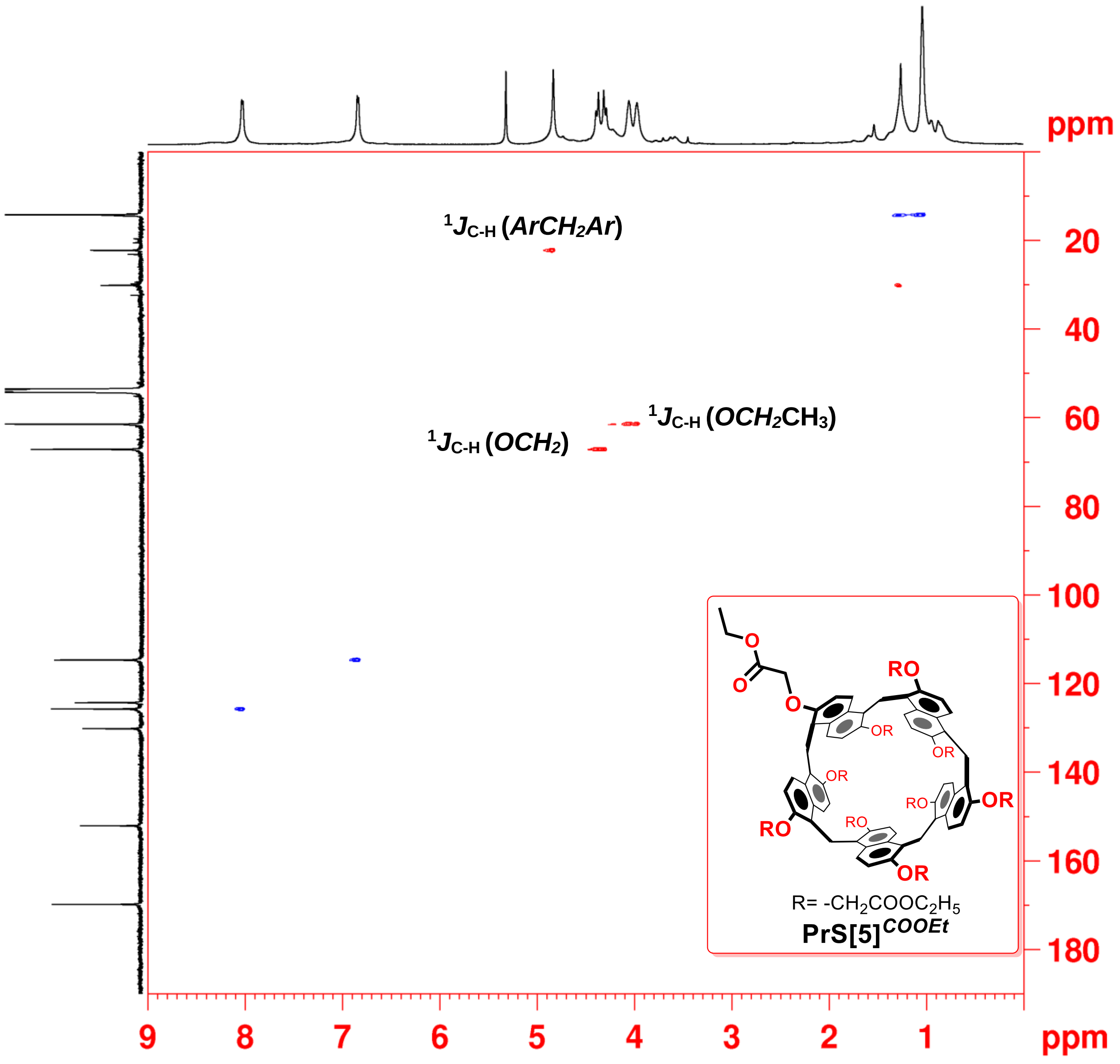

Figure S4: 2D-HSQC spectrum of **PrS[5]^{COOEt}** (CD_2Cl_2, 600 MHz, 298 K).

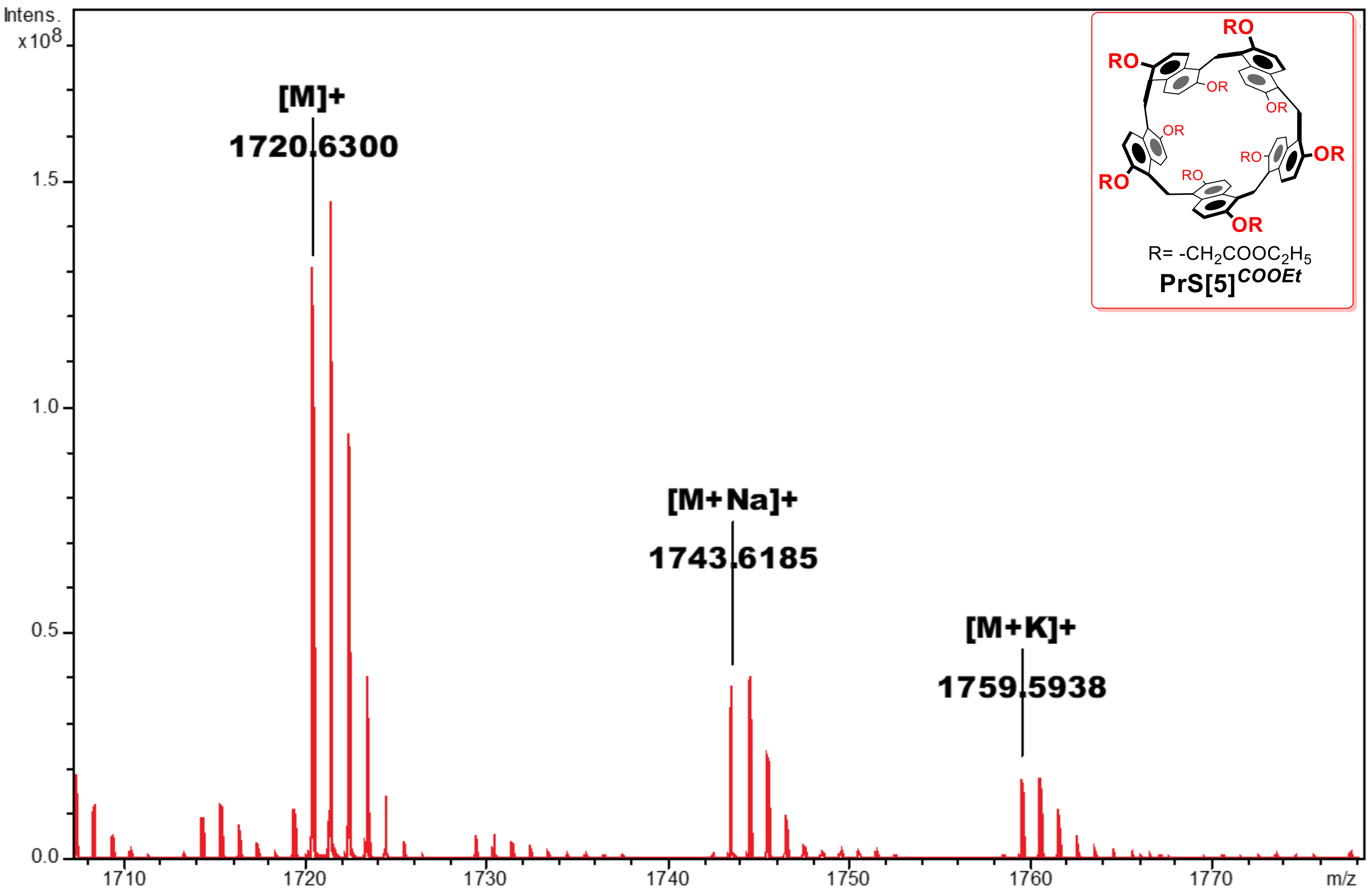

Figure S5: Significant portion of the HR MALDI FT-ICR mass spectrum of **PrS[5]**^{COOEt} $[M]^+$, $[M+Na]^+$ and $[M+K]^+$.

PrS[5]^{COOH}

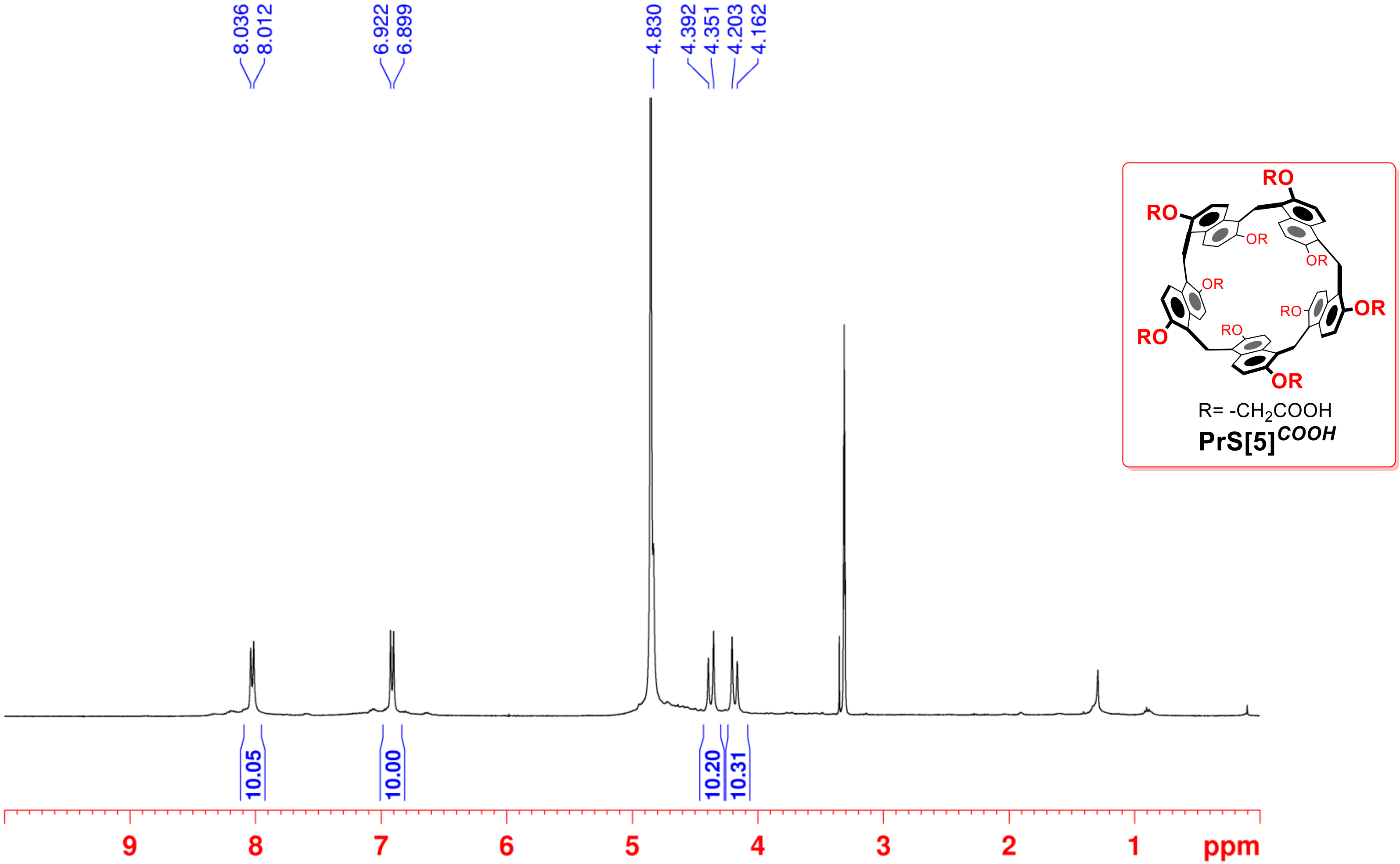

Figure S6: ^{1}H NMR spectrum of **PrS[5]^{COOH}** (CD_3OD, 400 MHz, 298 K).

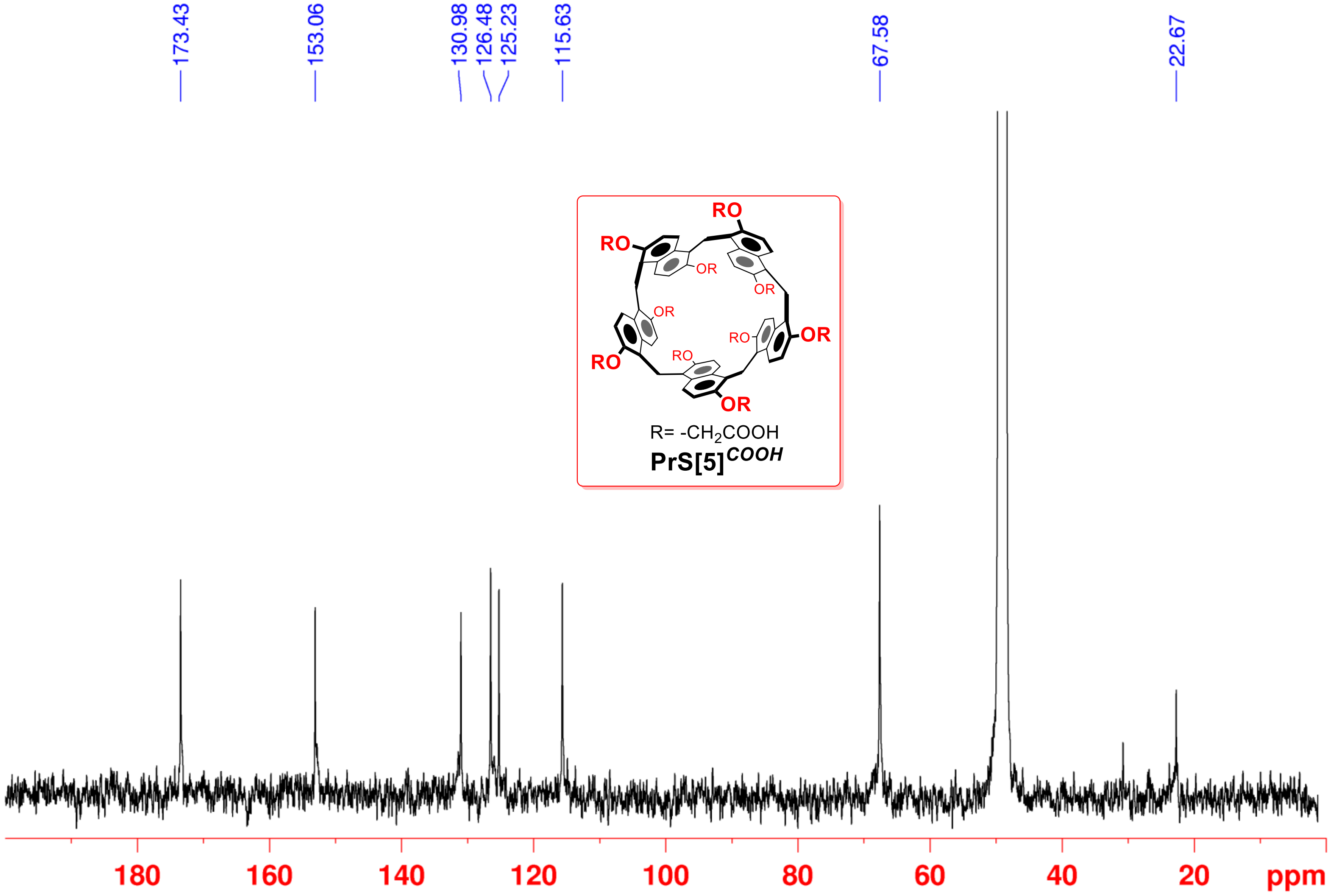

Figure S7: $^{13}C\{^1H\}$ NMR spectrum of **PrS[5]**^{COOH} (CD_3OD, 100 MHz, 298 K).

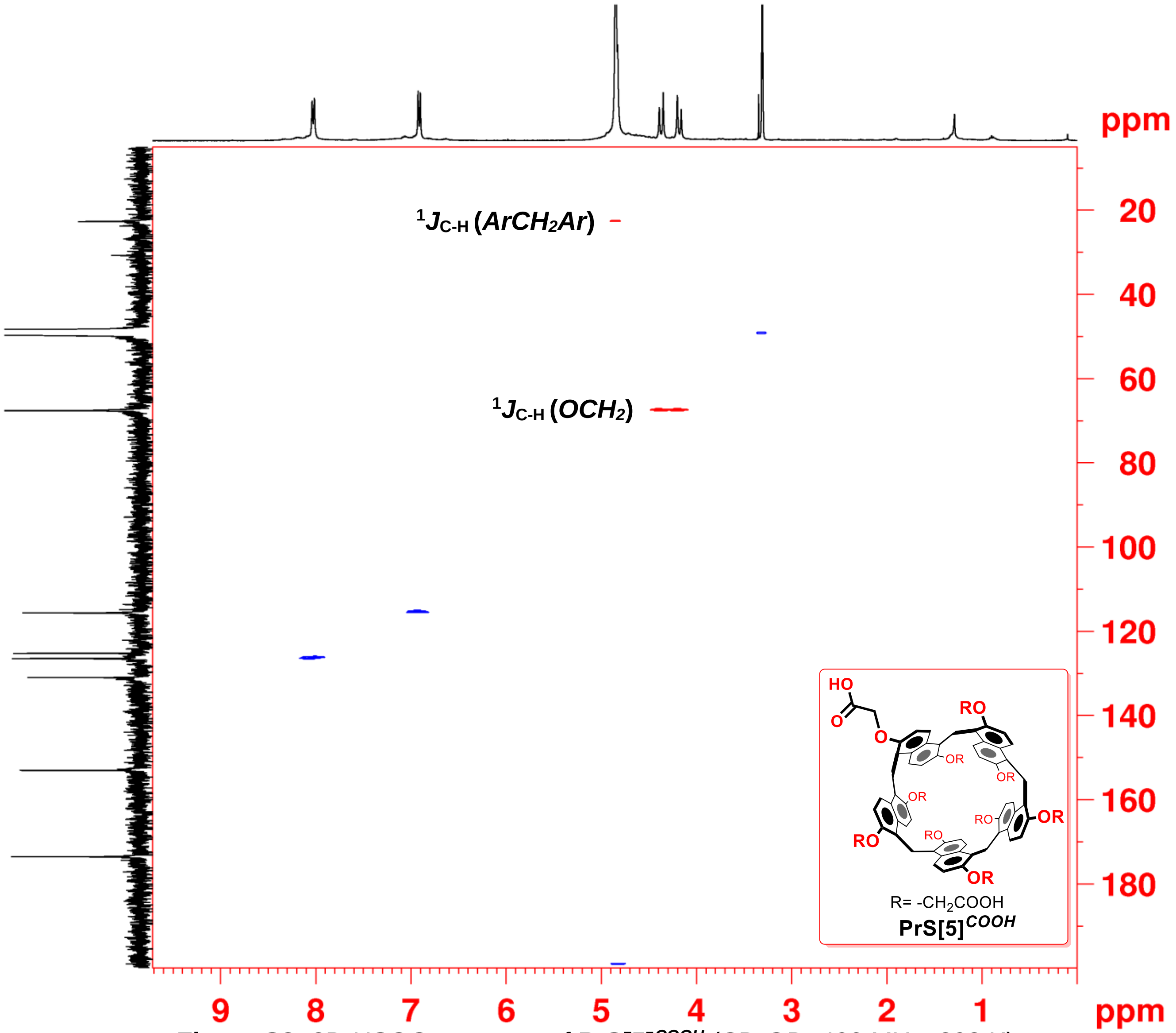

Figure S8: 2D-HSQC spectrum of **PrS[5]**^{COOH} (CD_3OD, 400 MHz, 298 K).

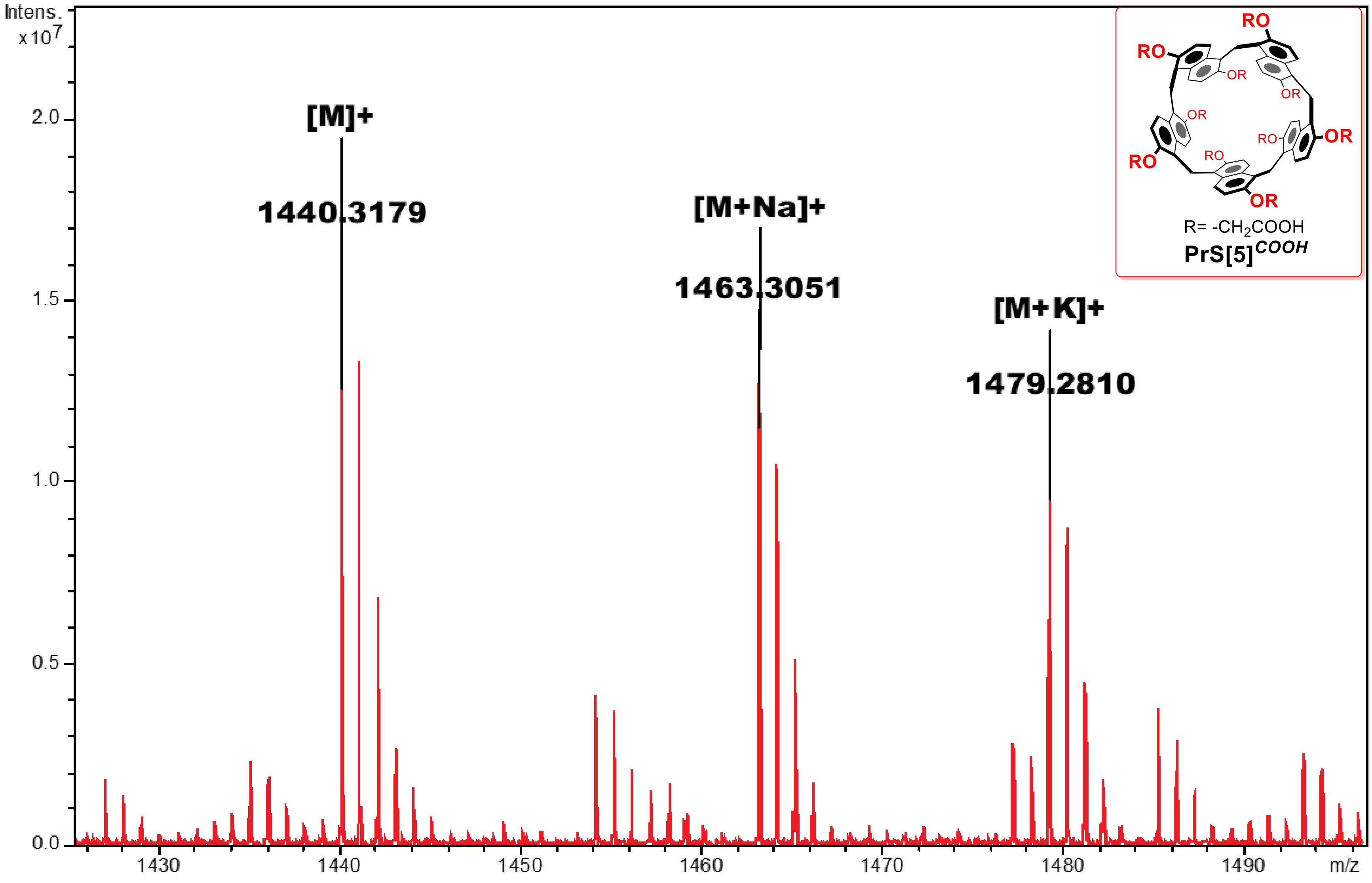

Figure S9: Significant portion of the HR MALDI FT-ICR mass spectrum of **PrS[5]**^{COOH} $[M]^+$, $[M+Na]^+$ and $[M+K]^+$.

PrS[5]^{COONa}

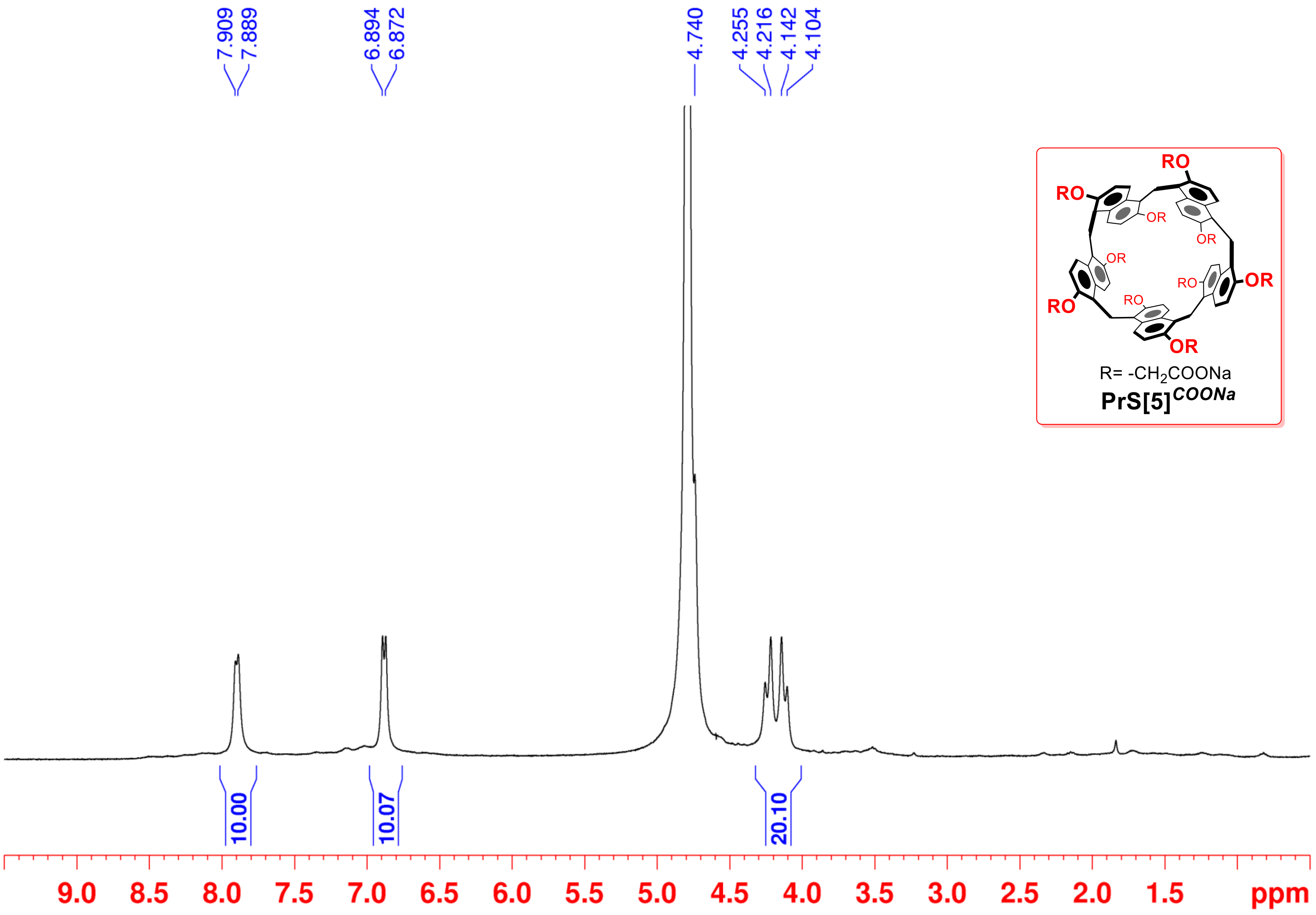

Figure S10: ^{1}H NMR spectrum of **PrS[5]^{COONa}** (D_2O, 400 MHz, 298 K).

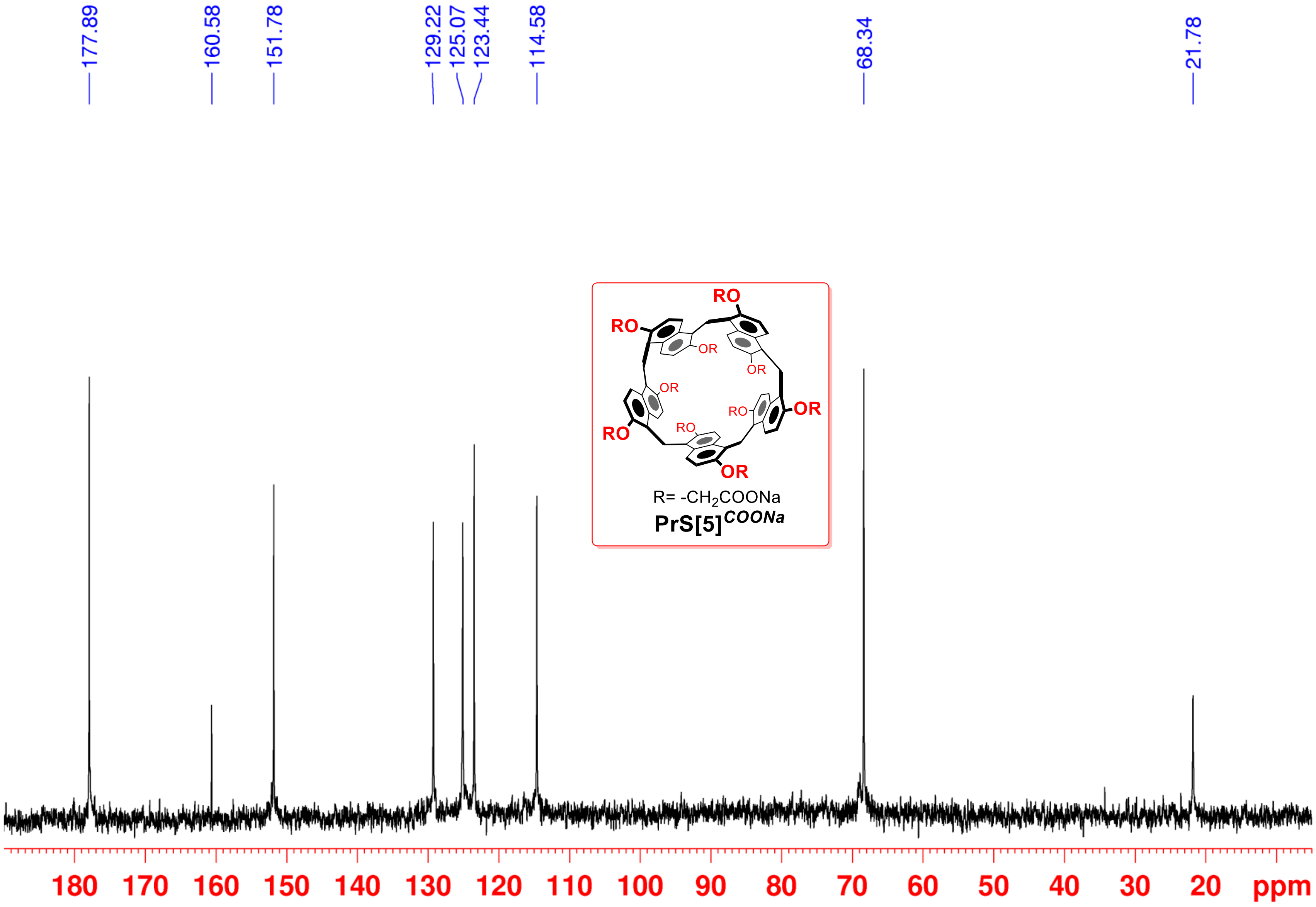

Figure S11: $^{13}C\{^1H\}$ NMR spectrum of **PrS[5]*COONa*** (D_2O, 100 MHz, 298 K).

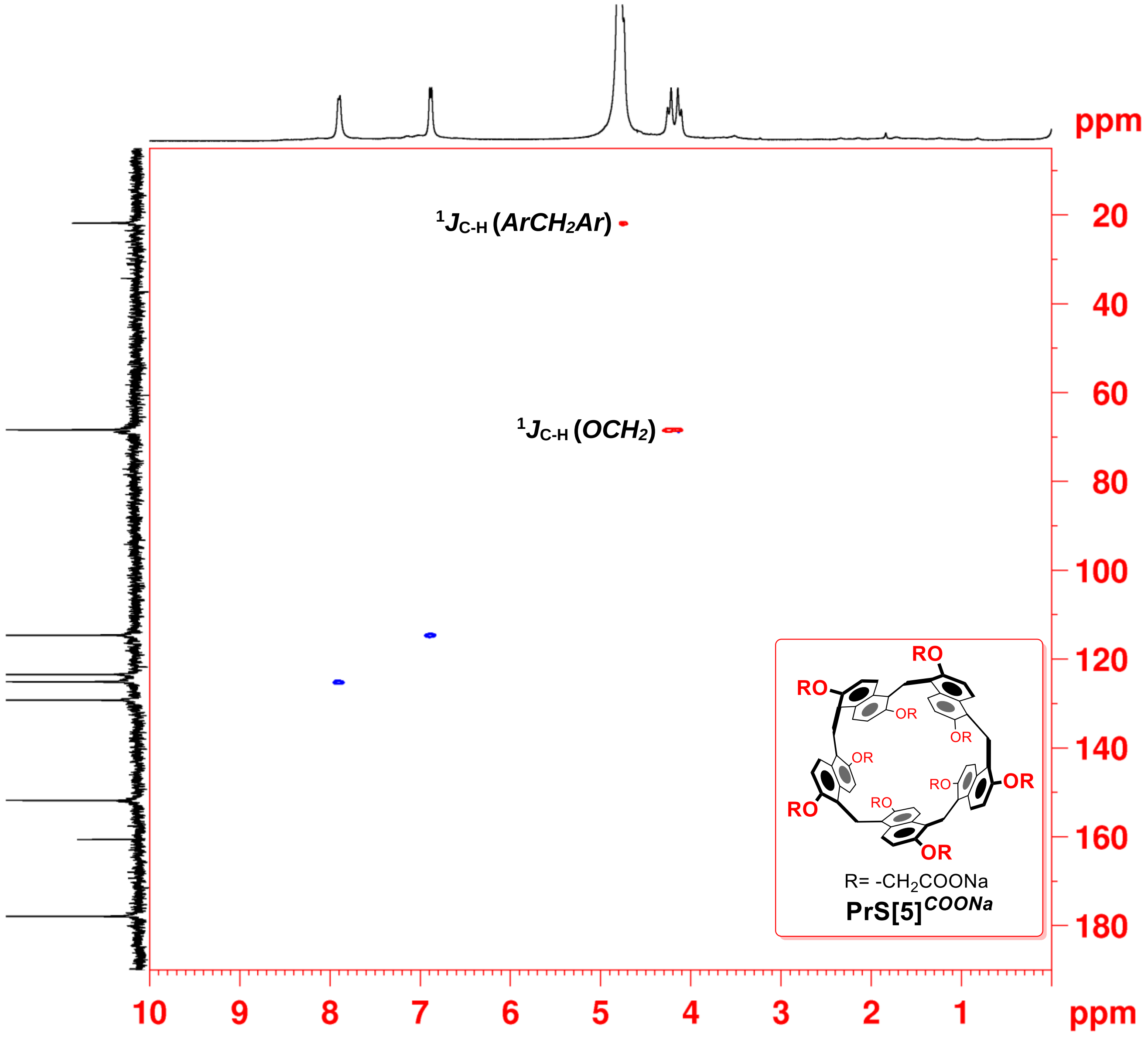

Figure S12: 2D-HSQC spectrum of **PrS[5]**^{COONa} (D_2O, 400 MHz, 298 K).

PrS[6]^{COOEt}

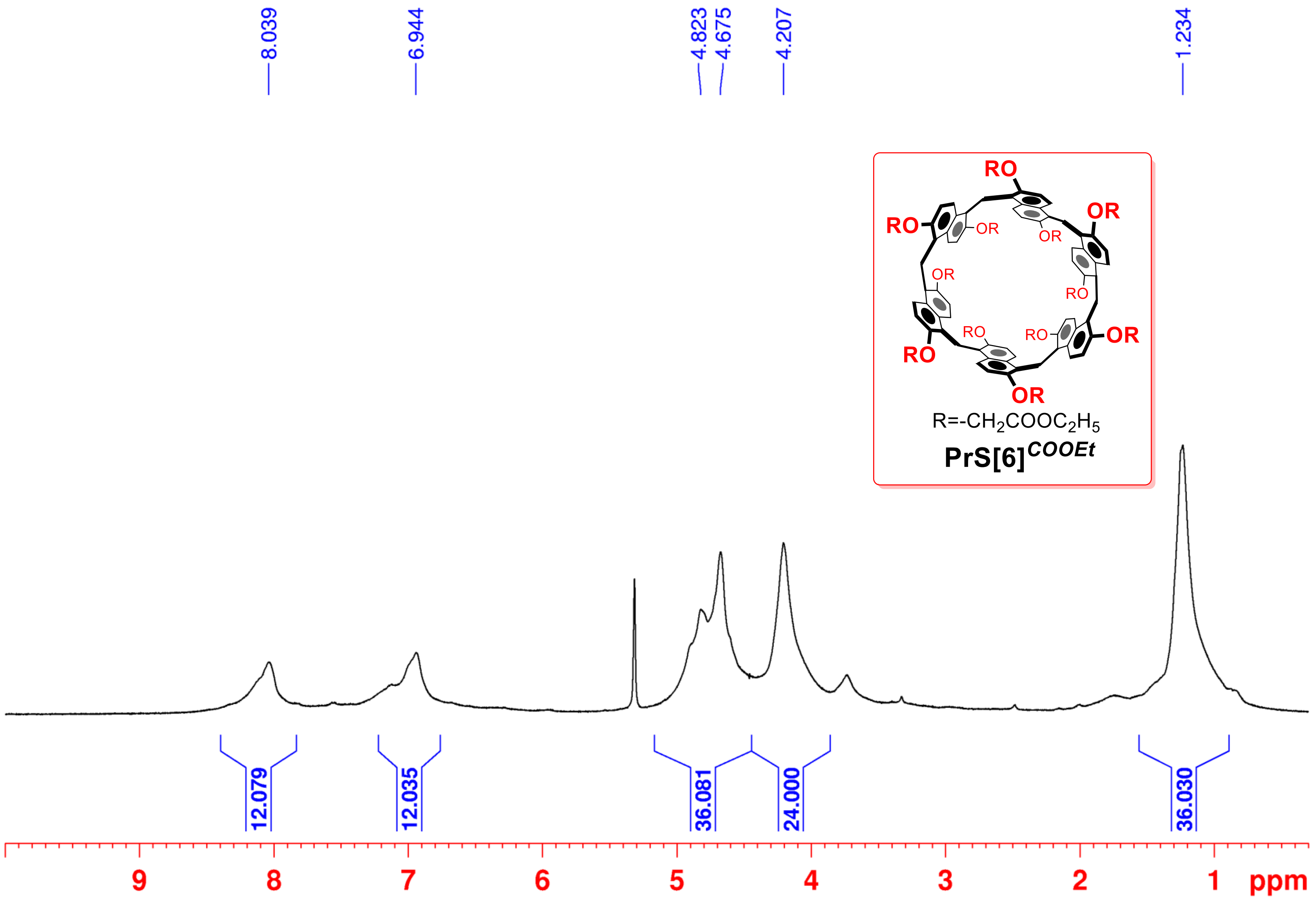

Figure S13: ^{1}H NMR spectrum of **PrS[6]^{COOEt}** (CD_2Cl_2, 400 MHz, 298 K).

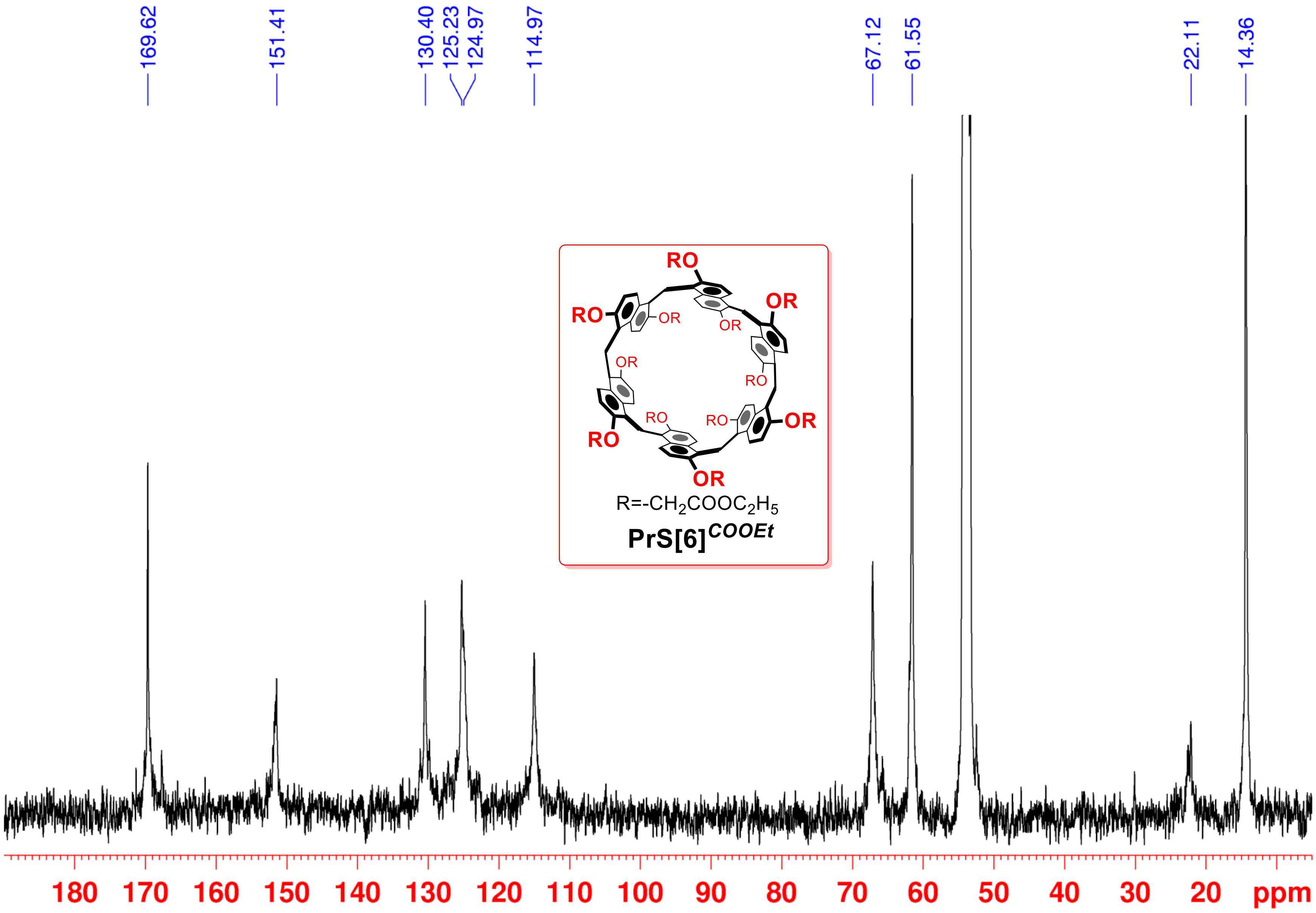

Figure S14: $^{13}C\{^1H\}$ NMR spectrum of **PrS[6]*COOEt*** (CD_2Cl_2, 100 MHz, 298 K).

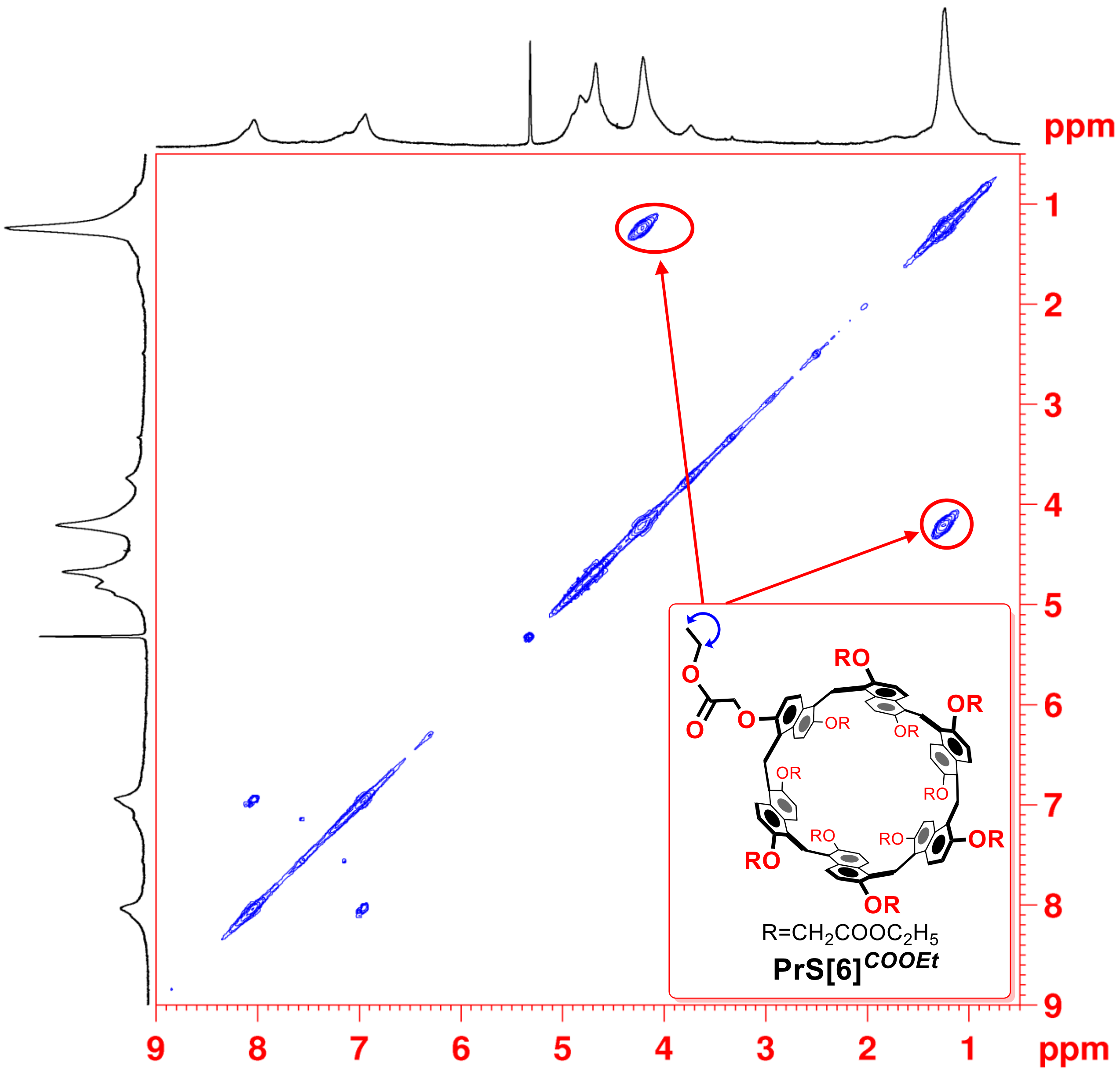

Figure S15: 2D-DQF COSY spectrum of **PrS[6]*COOEt*** (CD_2Cl_2, 400 MHz, 298 K).

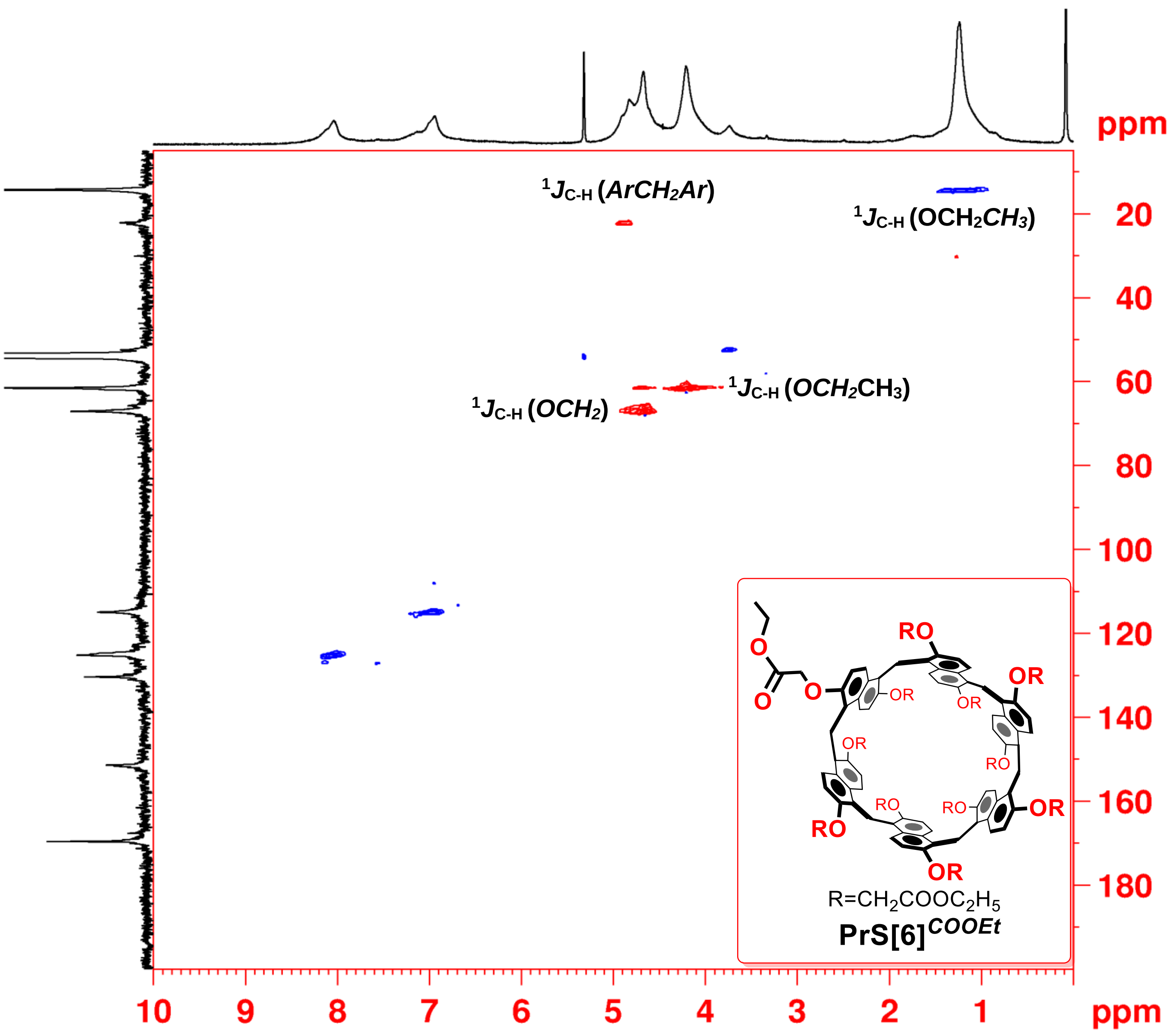

Figure S16: 2D-HSQC spectrum of **PrS[6]**^{COOEt} (CD_2Cl_2, 400 MHz, 298 K).

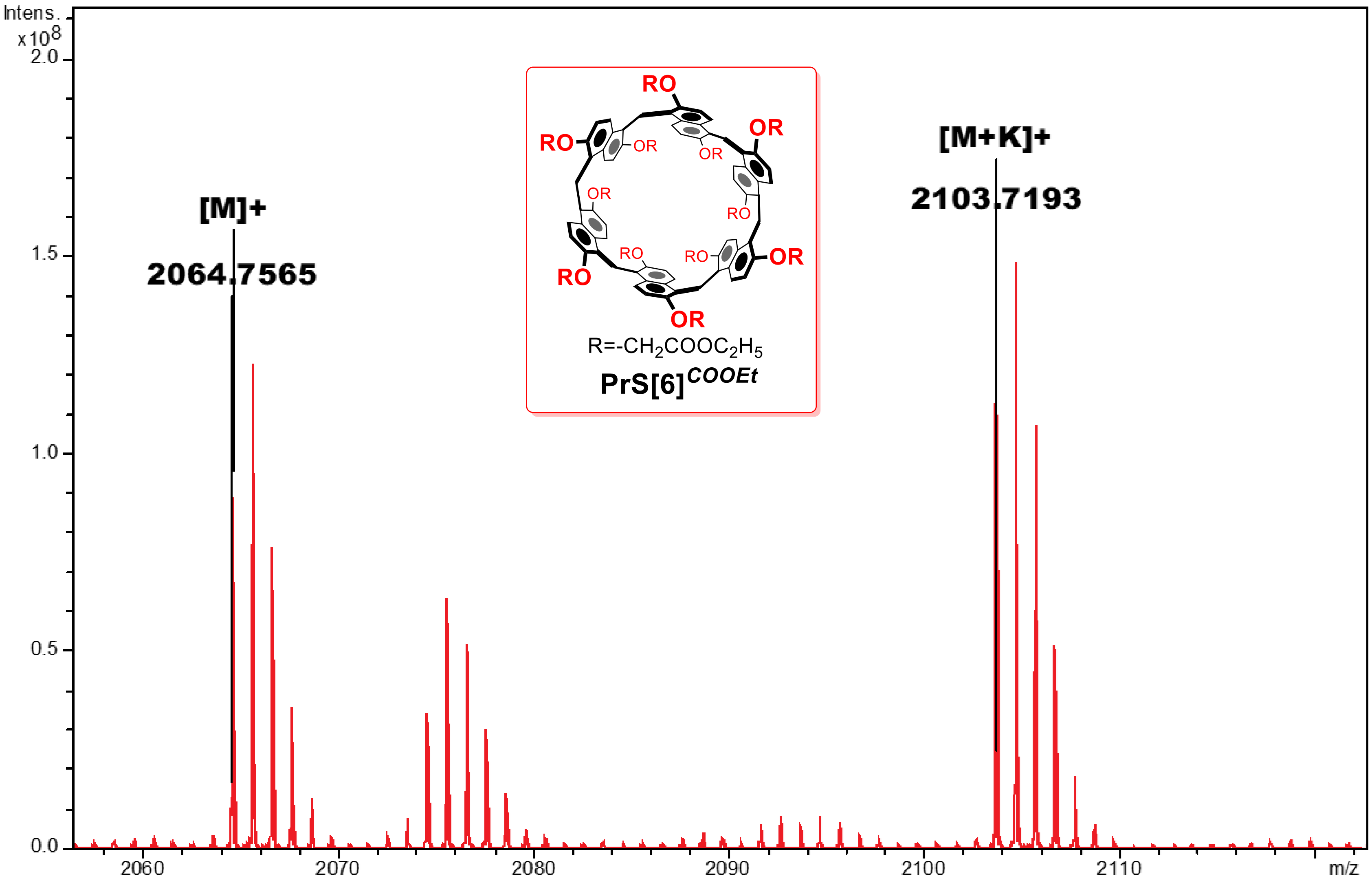

Figure S17: Significant portion of the HR MALDI FT-ICR mass spectrum of **PrS[6]*COOEt*** $[M]^+$ and $[M+K]^+$.

PrS[6]^{COOH}

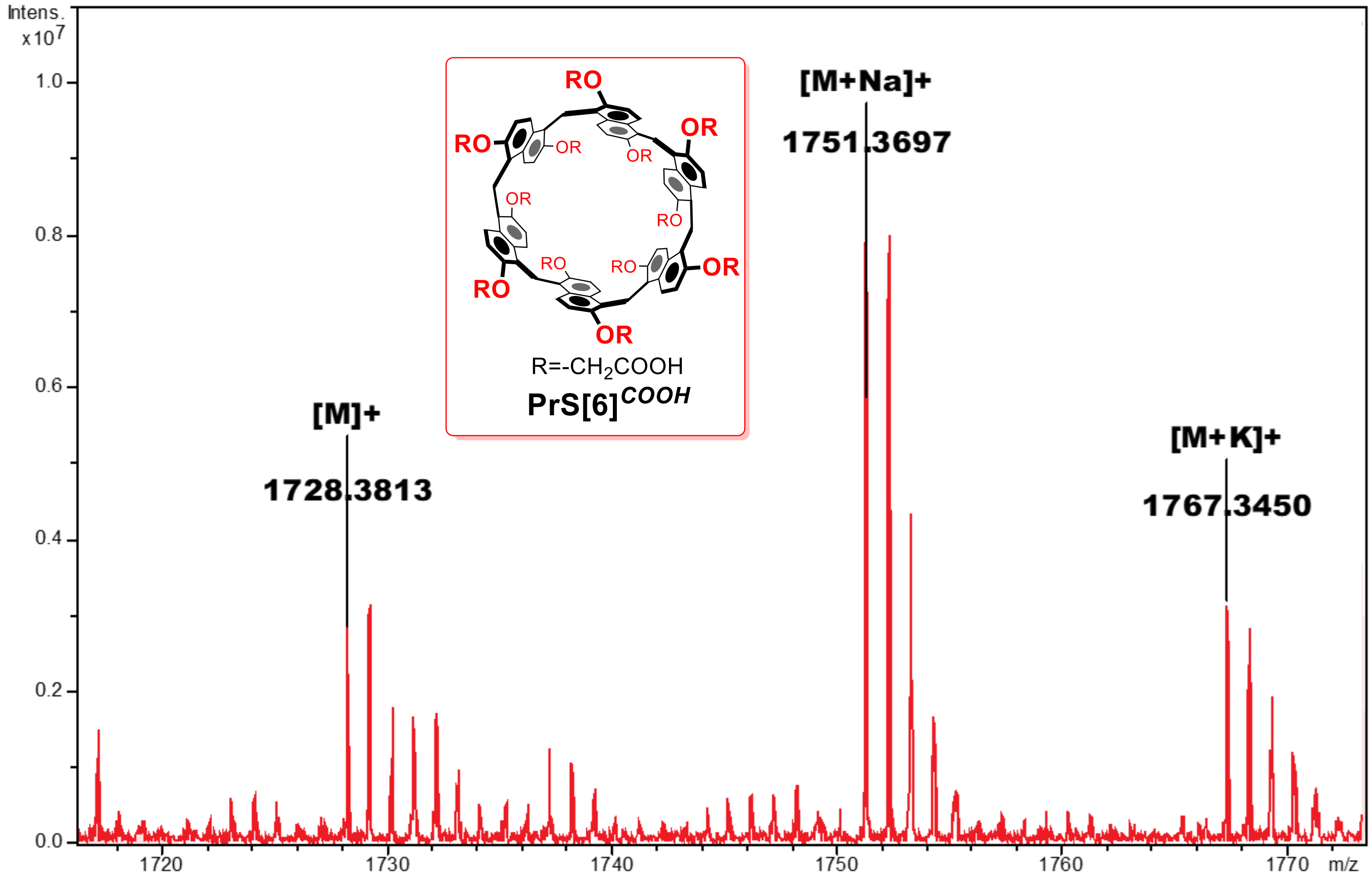

Figure S18: Significant portion of the HR MALDI FT-ICR mass spectrum of **PrS[6]^{COOH}** $[M]^+$, $[M+Na]^+$ and $[M+K]^+$.

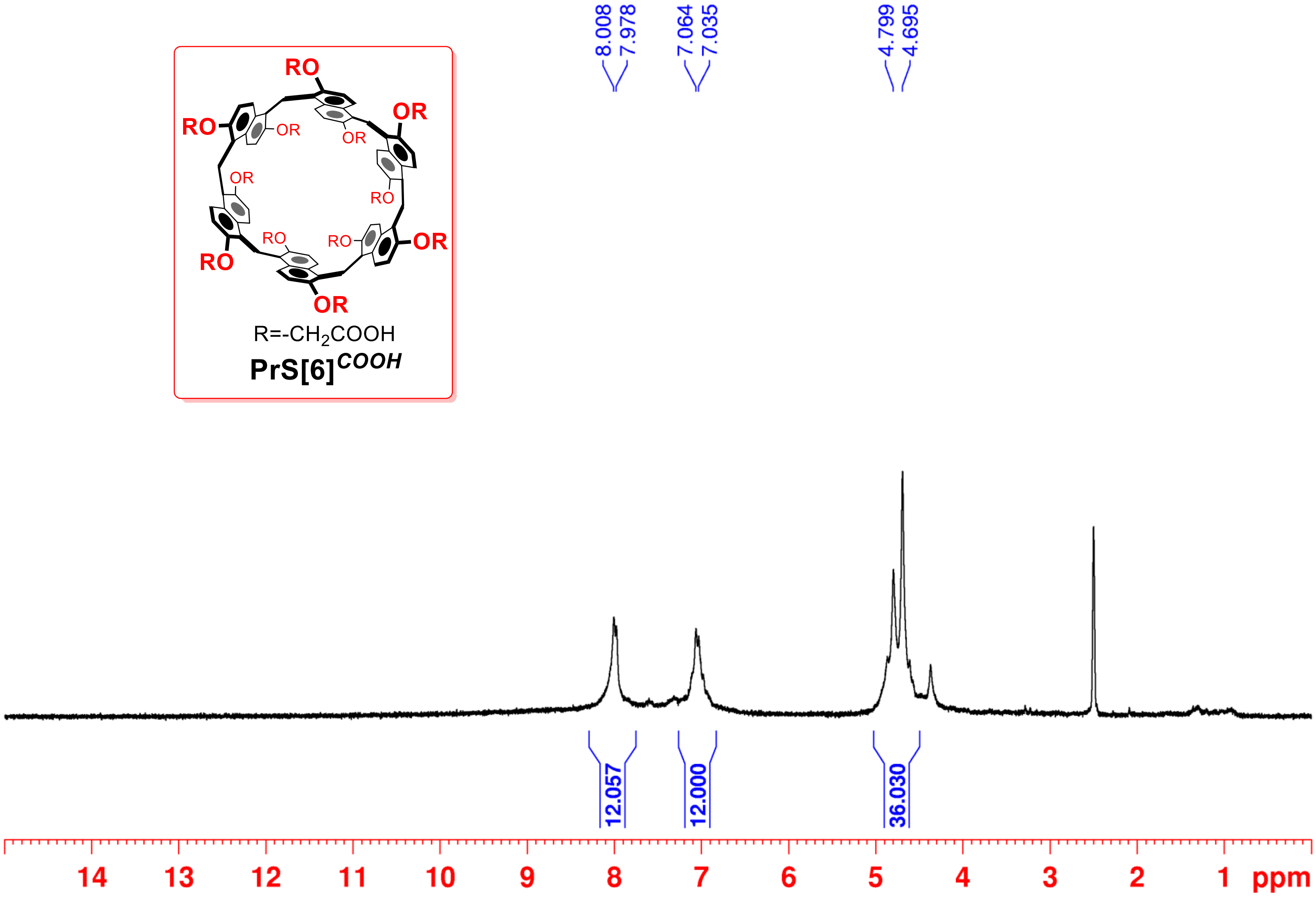

Figure S19: ^{1}H NMR spectrum of **PrS[6]^{COOH}** (DMSO-d_6, 300 MHz, 393 K).

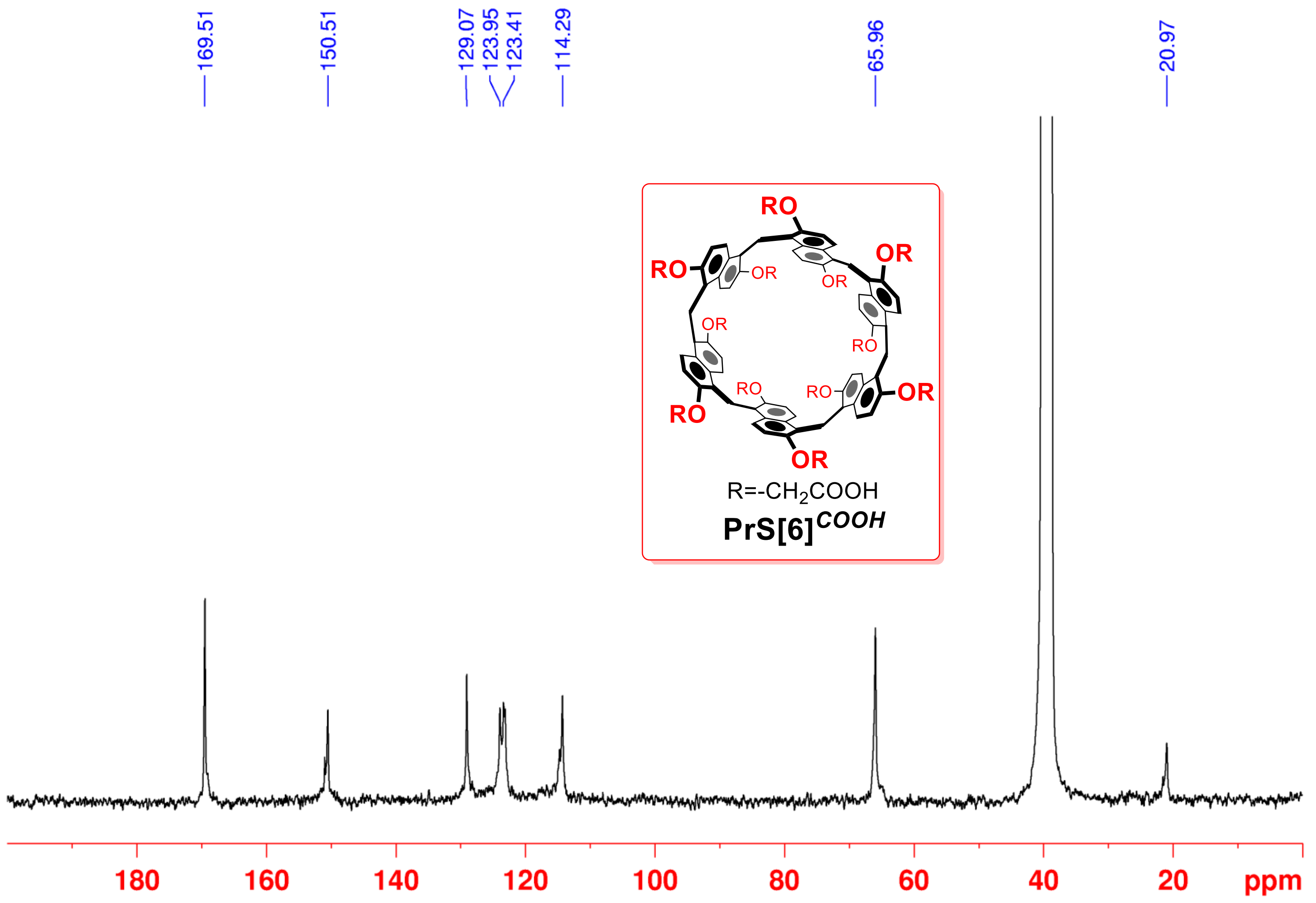

Figure S20: $^{13}C\{^{1}H\}$ NMR spectrum of **PrS[6]*^{COOH}*** (DMSO-d_6, 75 MHz, 393 K).

PrS[6]^{COONa}

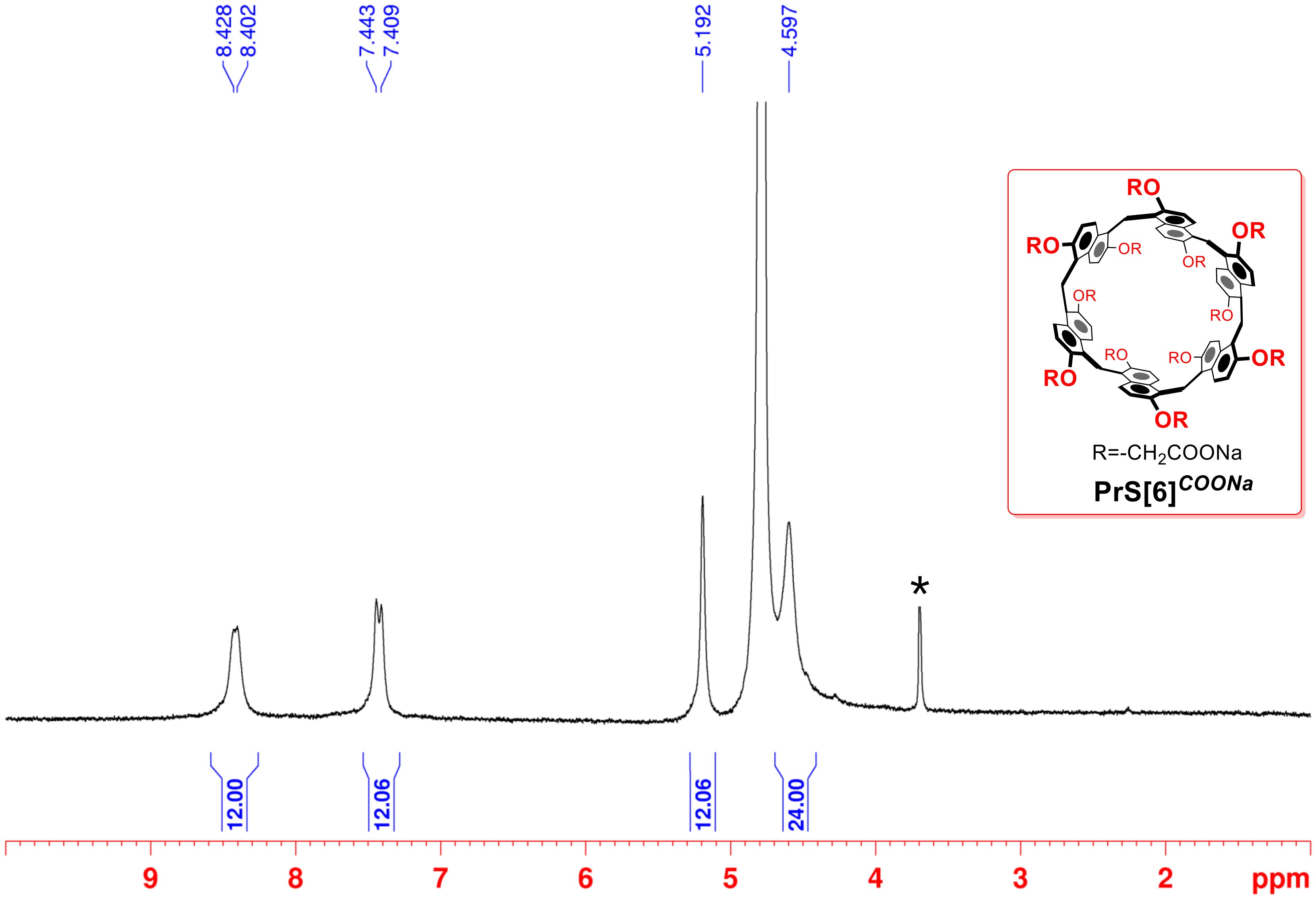

Figure S21: ^{1}H NMR spectrum of **PrS[6]**^{COONa} (D_2O, 250 MHz, 333 K) *residual purification solvent.

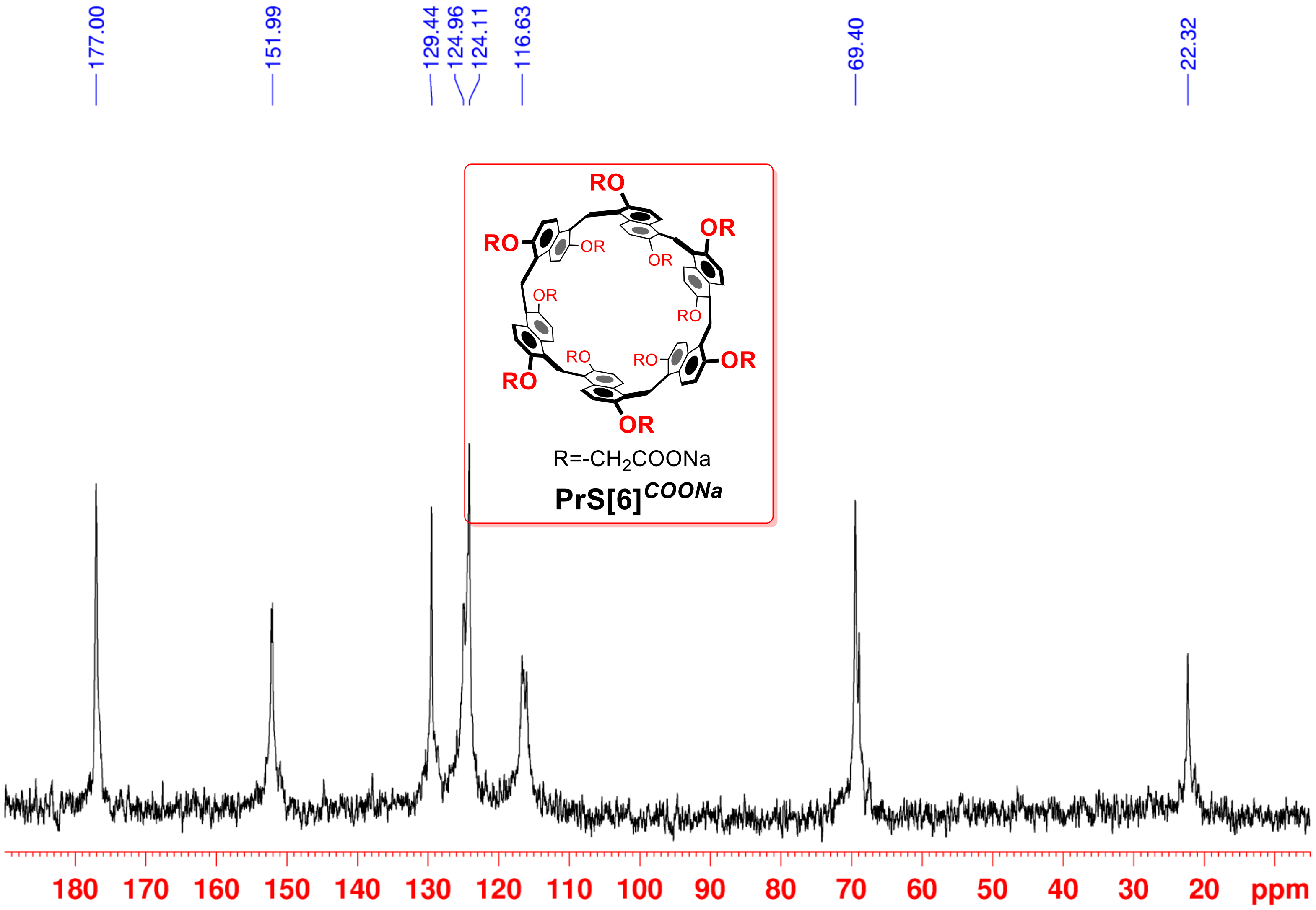

Figure S22: $^{13}C\{^1H\}$ NMR spectrum of **PrS[6]*^{COONa}*** (D_2O, 75 MHz, 333 K).

1D NMR titration experiments for the formation of the G^{n+}@PrS[n]$^{COO-}$ complexes

The samples were prepared by dissolving **PrS[5]^{COONa}** or **PrS[6]^{COONa}** (6.02 mM) and the appropriate ammonium guest **1^{2+} - 7^{2+}** as iodide salt (6.02 mM) in an aqueous deuterated phosphate buffer (68 mM) at pH 7.6. This solution (0.5 mL) was placed in an NMR tube and was equilibrated for 24 h at 40 °C.

7^{2+}@ PrS[5]$^{COO-}$

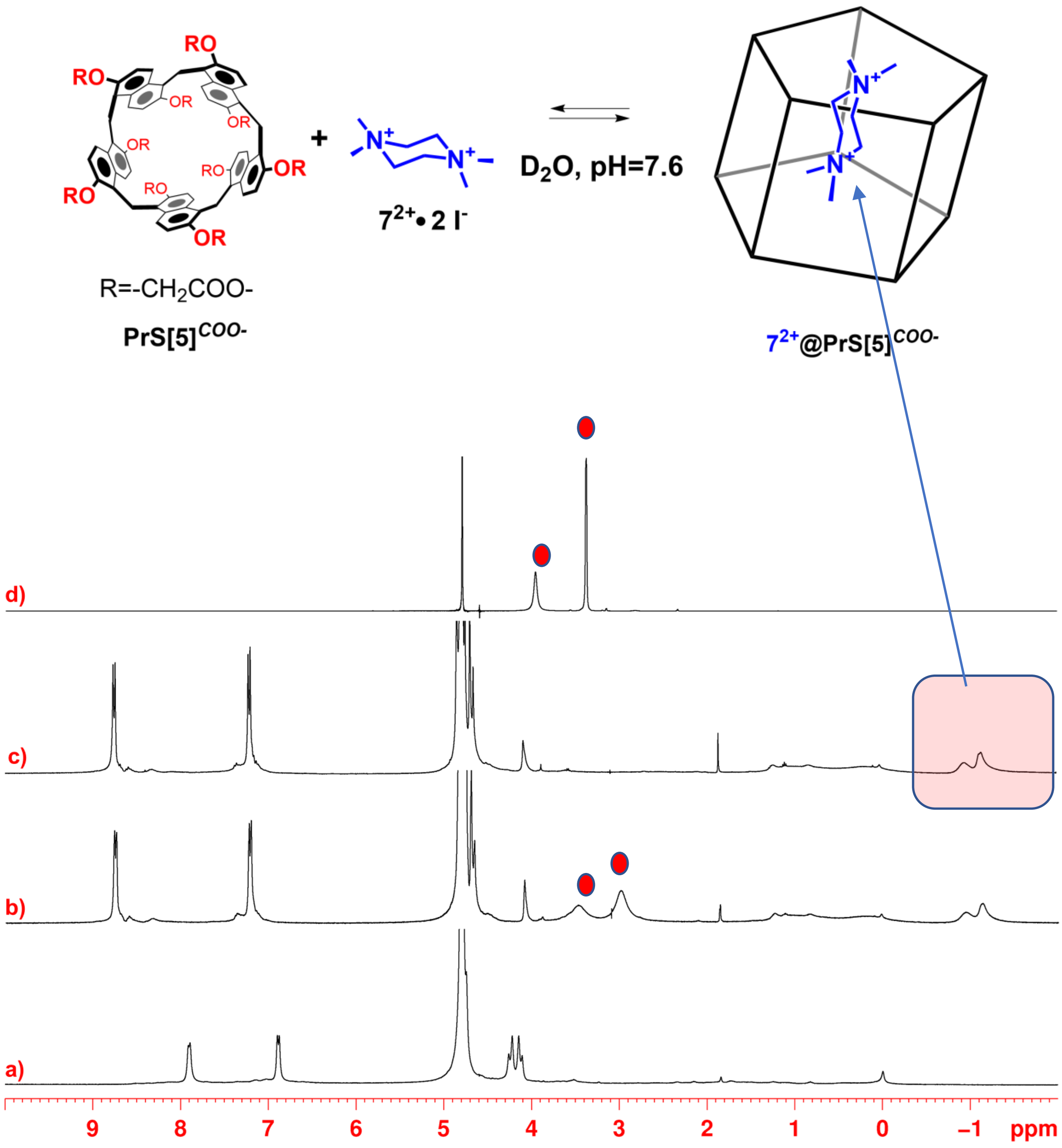

Figure S23: ^{1}H NMR spectra (400 MHz, buffered D_2O solution, pH 7.60, 298 K) of: (a) **PrS[5]$^{COO-}$**; (b) 1:2 mixture of **PrS[5]$^{COO-}$** and **7^{2+} · 2I$^-$**, marked in red the ^{1}H NMR signals of the free guest **7^{2+}**; (c) an equimolar solution of **PrS[5]$^{COO-}$** and **7^{2+} · 2I$^-$**; and (d) **7^{2+} · 2I$^-$**.

4^{2+}@ PrS[5]$^{COO-}$

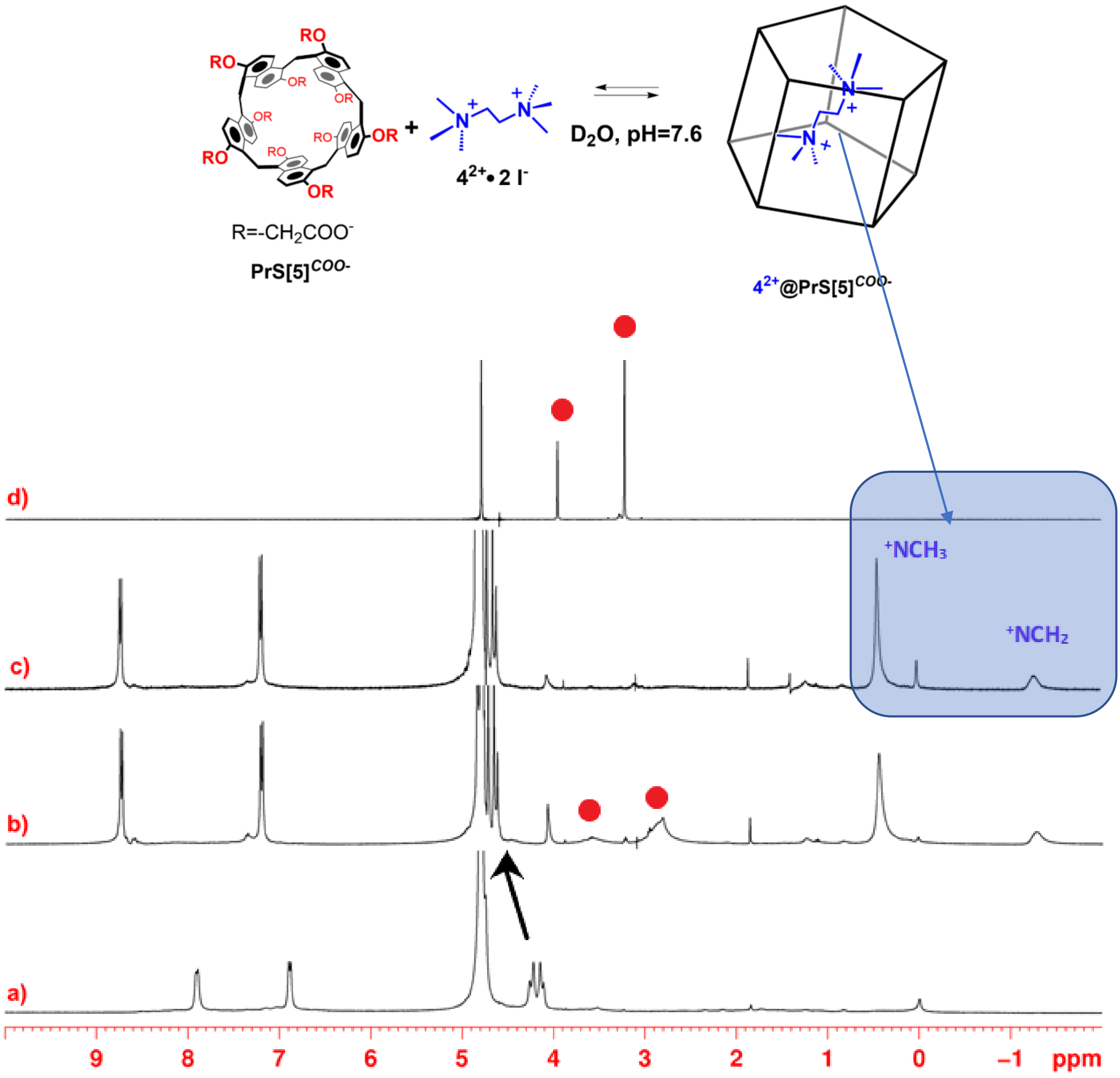

Figure S24: ^{1}H NMR spectra (400 MHz, buffered D_2O solution, pH 7.60, 298 K) of: (a) **PrS[5]$^{COO-}$**, (b) 1:2 mixture of **PrS[5]$^{COO-}$** and **4^{2+} · $2I^-$**, marked in red the ^{1}H NMR signals of the free guest **4^{2+}**; (c) an equimolar solution of **PrS[5]$^{COO-}$** and **4^{2+} · $2I^-$**; and (d) **4^{2+} · $2I^-$**.

1^{2+}@ PrS[5]$^{COO-}$

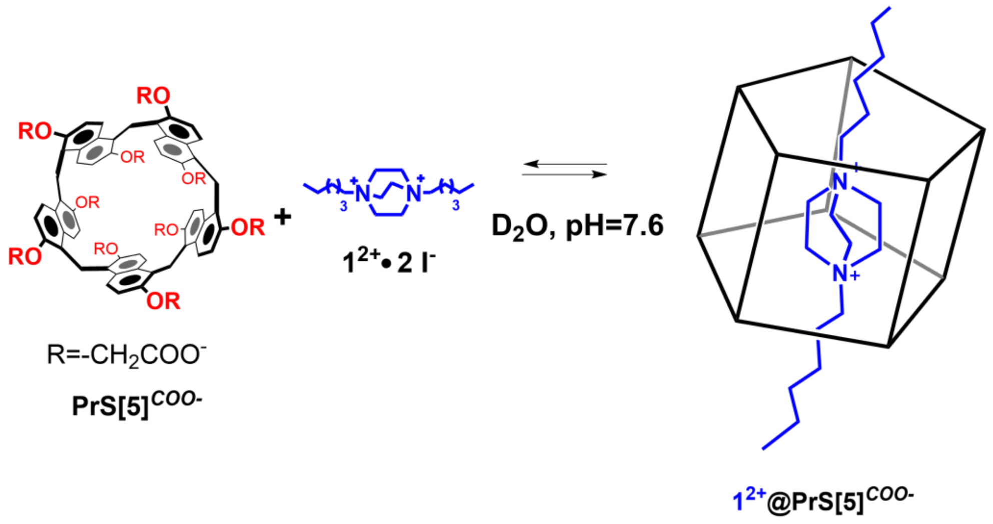

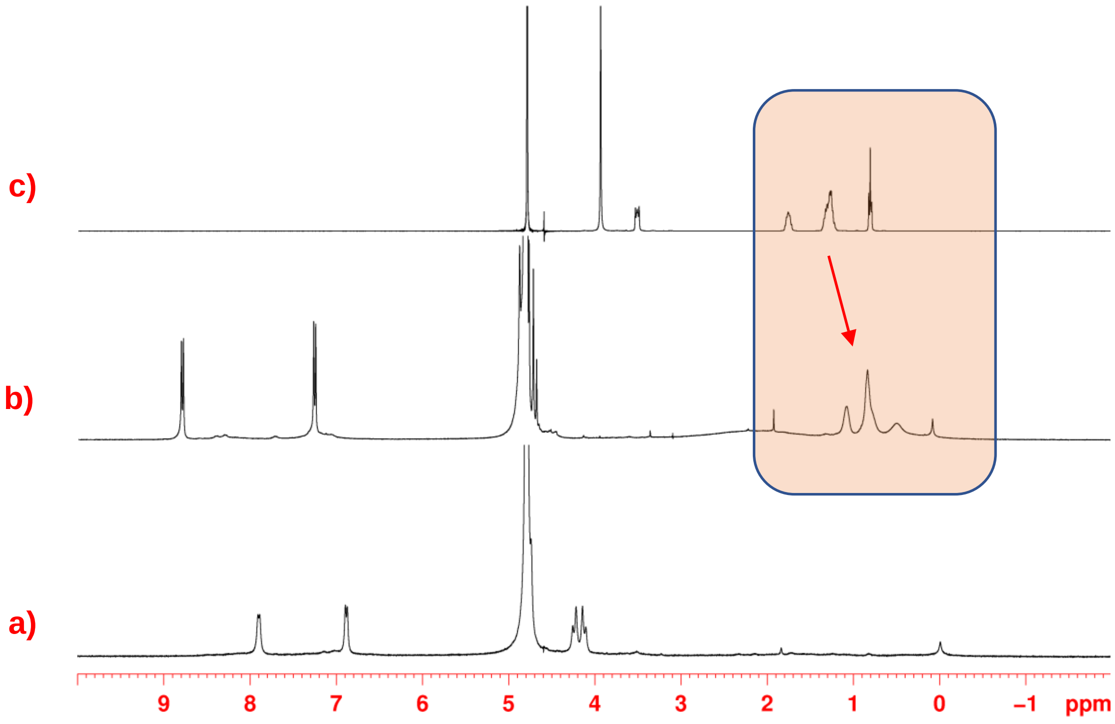

Figure S25: ^{1}H NMR spectra (400 MHz, buffered D_2O solution, pH 7.60, 298 K) of: (a) **PrS[5]$^{COO-}$**, (b) an equimolar solution of **PrS[5]$^{COO-}$** and **1^{2+} · 2I$^-$** and (c) **1^{2+} · 2I$^-$** .

3^{2+}@ PrS[5]$^{COO-}$

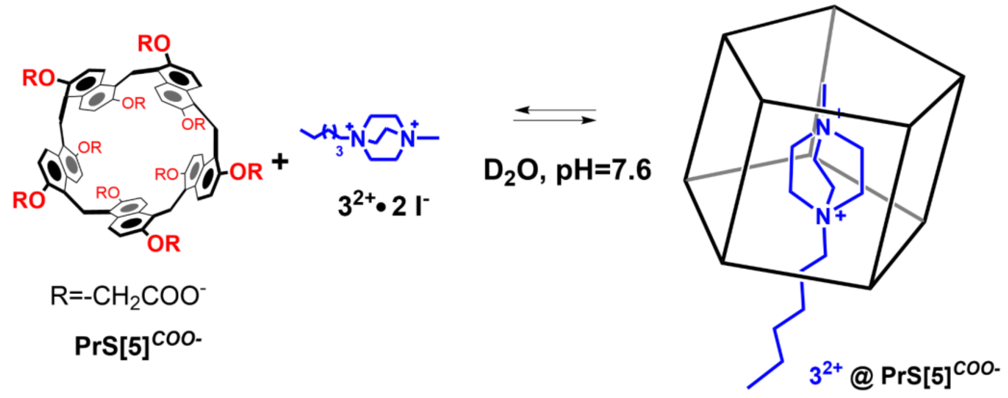

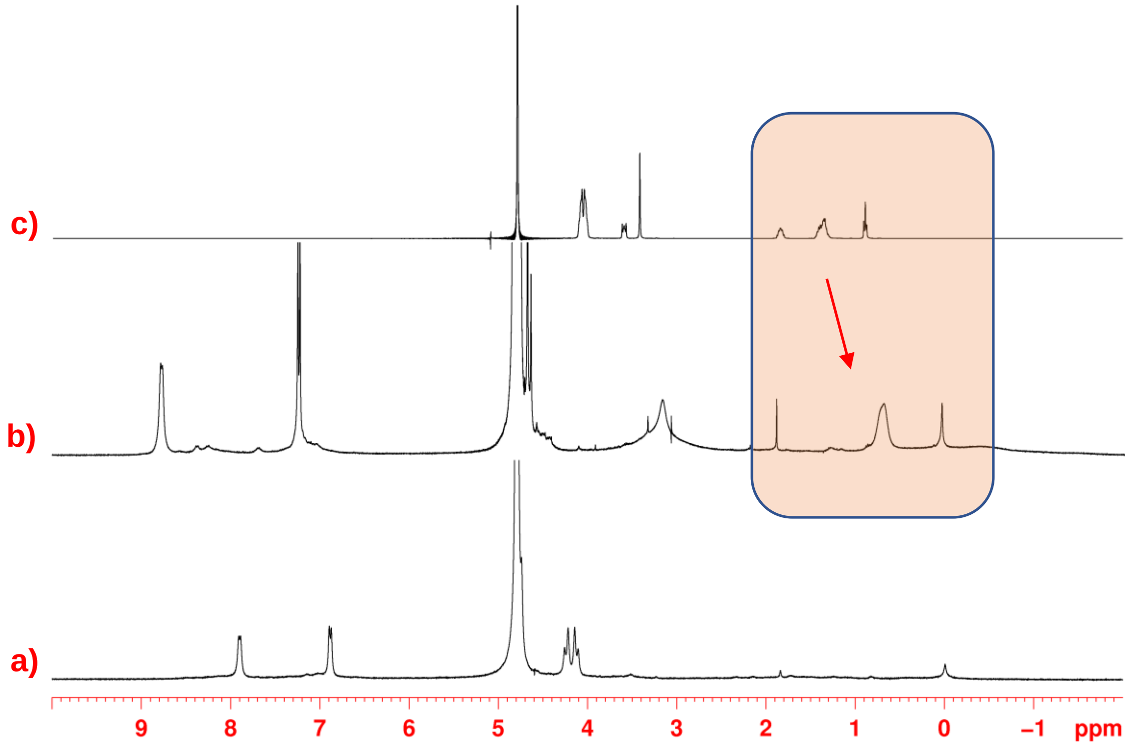

Figure S26: ^{1}H NMR spectra (400 MHz, buffered D_2O solution, pH 7.60, 298 K) of: (a) **PrS[5]$^{COO-}$**, (b) an equimolar solution of **PrS[5]$^{COO-}$** and **3^{2+} · 2I$^-$** and (c) **3^{2+} · 2I$^-$**.

6^+@PrS[5]$^{COO-}$

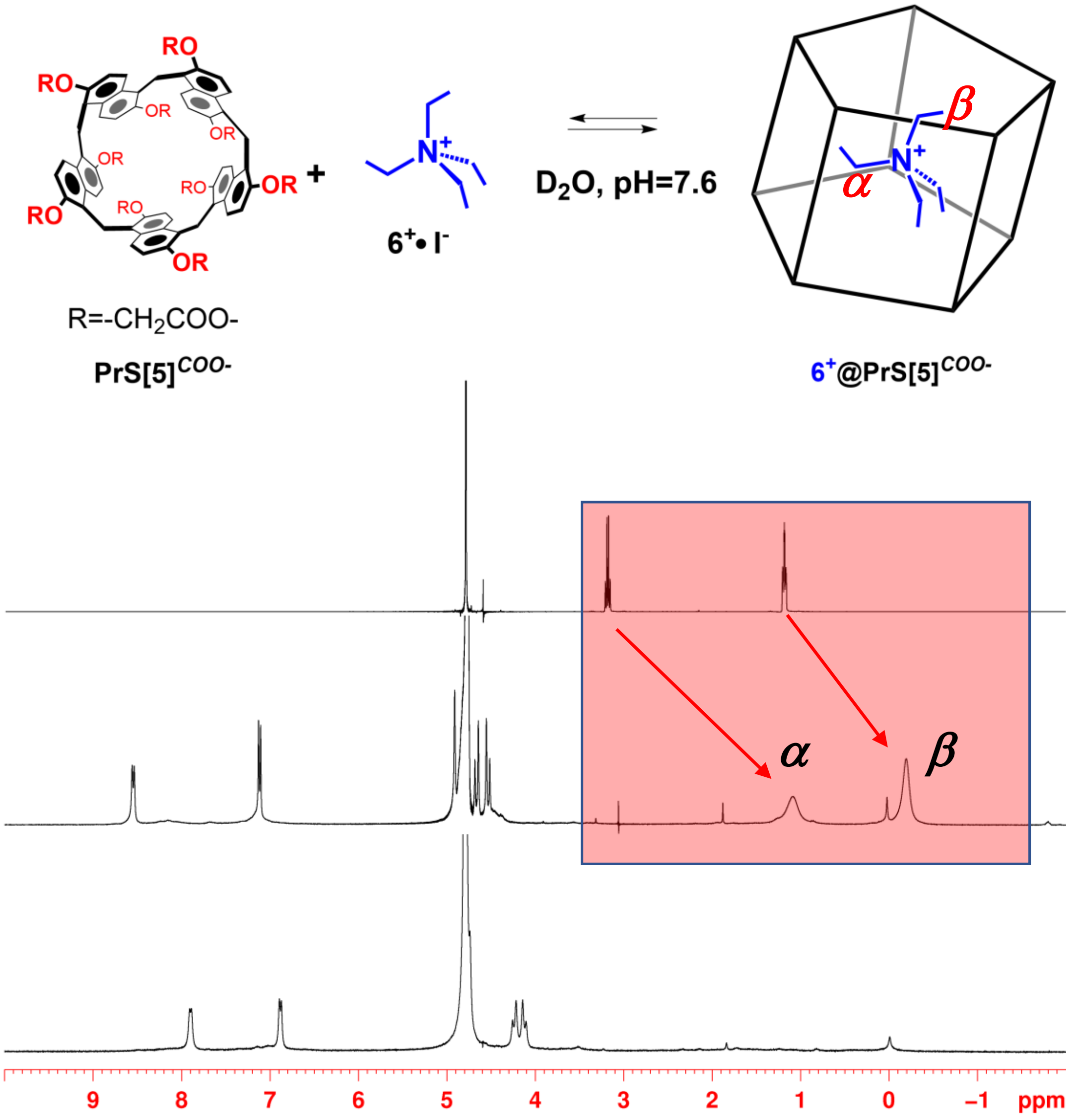

Figure S27: ^{1}H NMR spectra (400 MHz, buffered D_2O solution, pH 7.60, 298 K) of: (a) **PrS[5]$^{COO-}$**, (b) an equimolar solution of **PrS[5]$^{COO-}$** and **6^+ · I^-** and (c) **6^+ · I^-**.

5^+@ PrS[5]$^{COO-}$

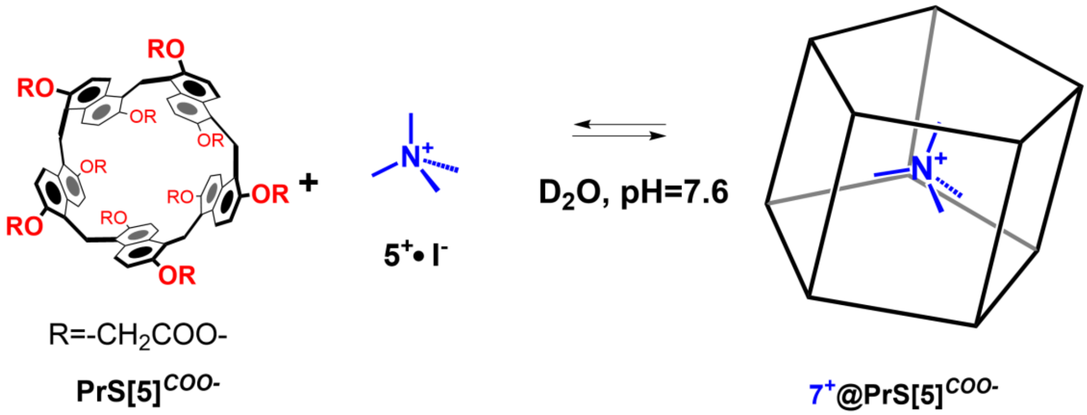

R=-CH_2COO-

PrS[5]$^{COO-}$

7^+@PrS[5]$^{COO-}$

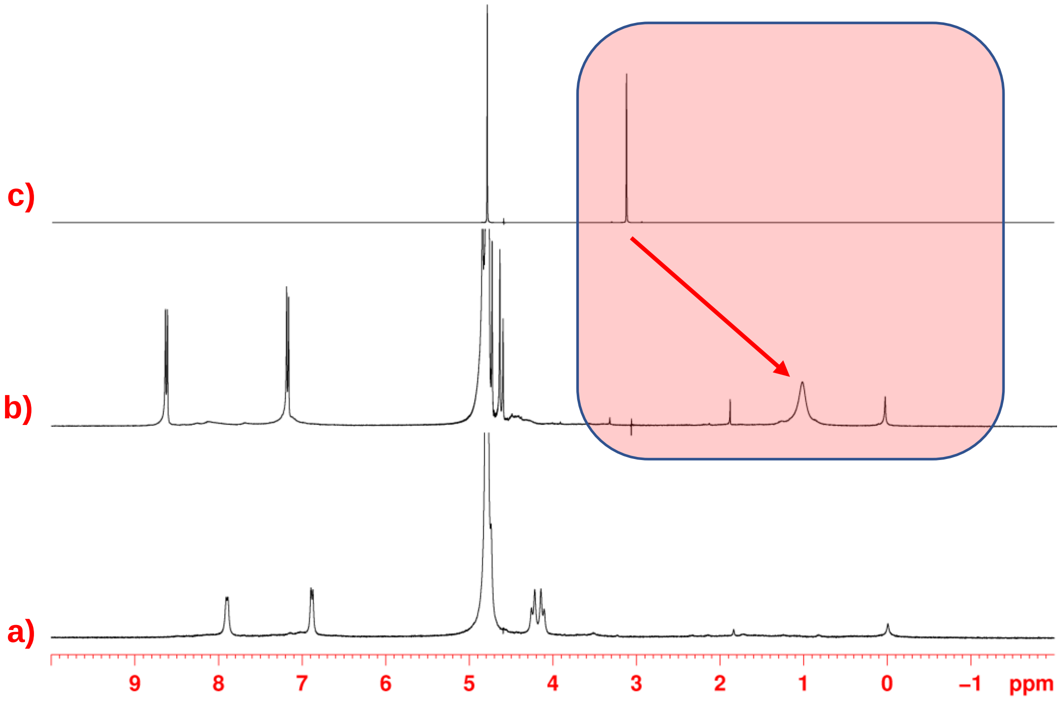

Figure S28: ^{1}H NMR spectra (400 MHz, buffered D_2O solution, pH 7.60, 298 K) of: (a) **PrS[5]$^{COO-}$**, (b) an equimolar solution of **PrS[5]$^{COO-}$** and **$5^+ \cdot I^-$** and (c) **$5^+ \cdot I^-$**.

1^{2+}@ **PrS[6]$^{COO-}$**

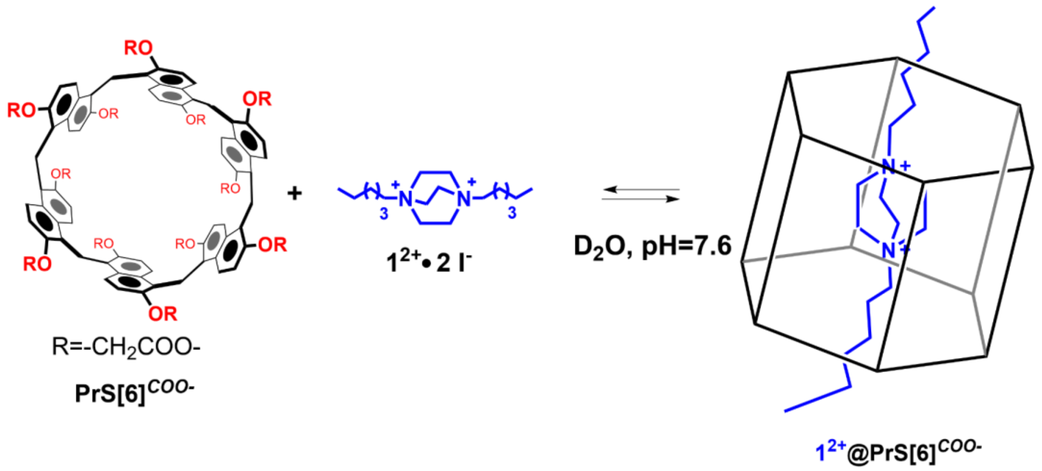

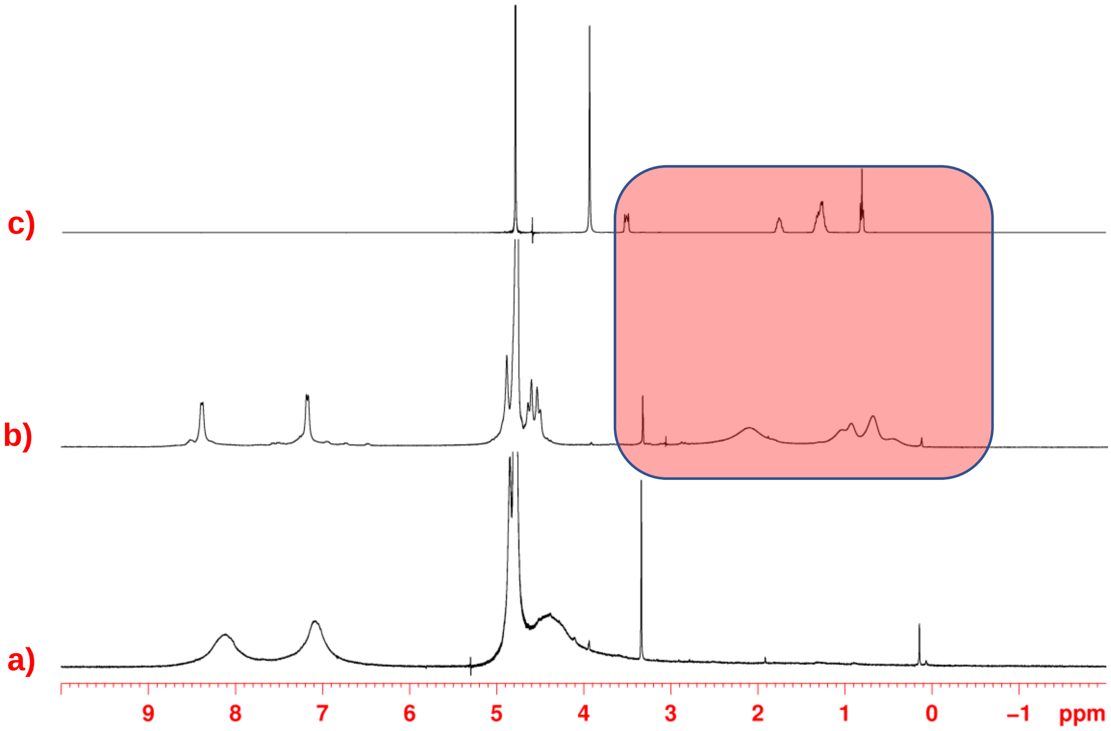

Figure S29: ^{1}H NMR spectra (400 MHz, buffered D_2O solution, pH 7.60, 298 K) of: (a) **PrS[6]$^{COO-}$**, (b) an equimolar solution of **PrS[6]$^{COO-}$** and **1^{2+} · 2I^{-}** and (c) **1^{2+} · 2I^{-}** .

2^{2+}@ PrS[6]$^{COO-}$

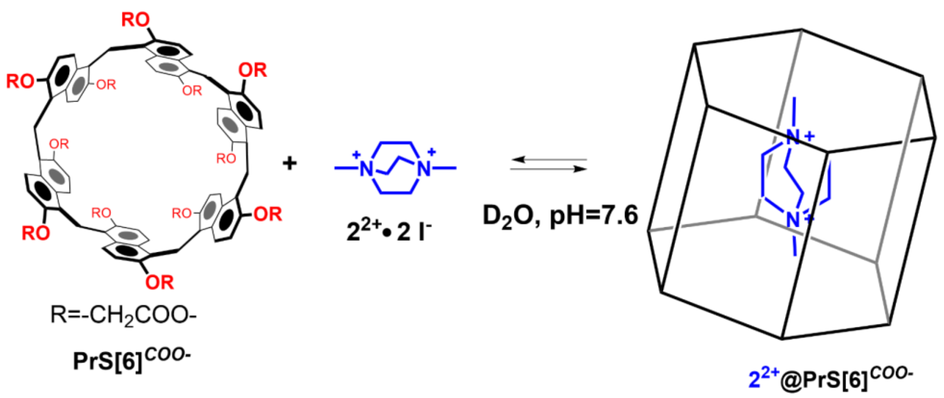

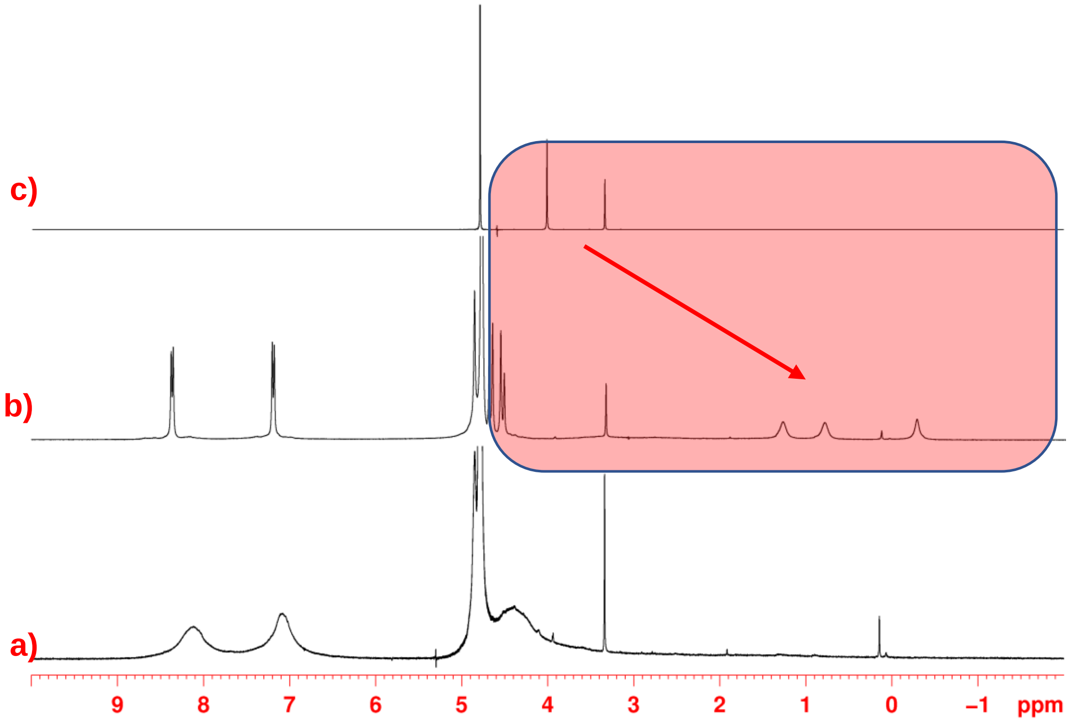

Figure S30: ^{1}H NMR spectra (400 MHz, buffered D_2O solution, pH 7.60, 298 K) of: (a) **PrS[6]$^{COO-}$**, (b) an equimolar solution of **PrS[6]$^{COO-}$** and **2^{2+} · 2I^{-}** and (c) **2^{2+} · 2I^{-}** .

5^{2+}@ PrS[6]$^{COO-}$

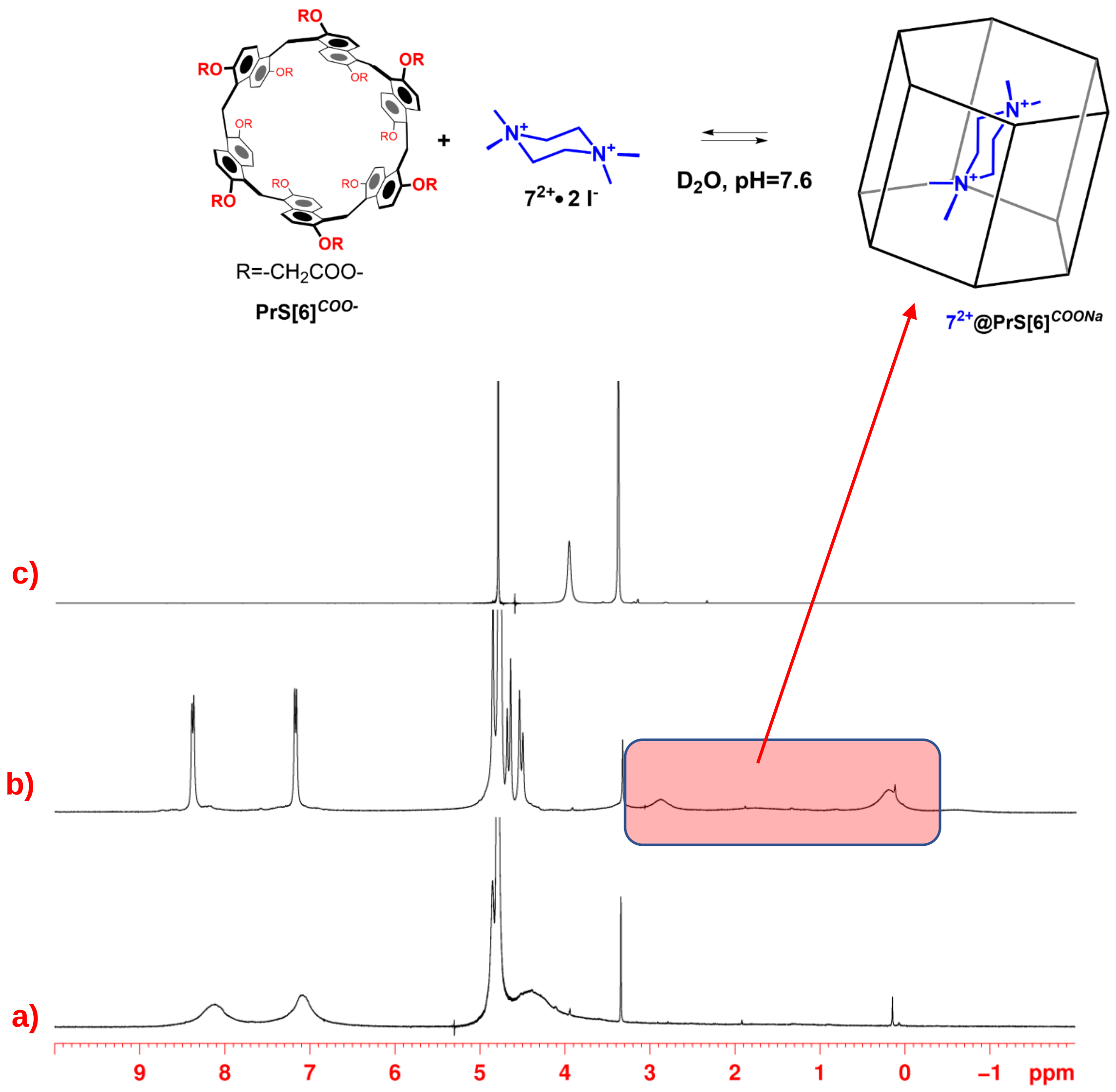

Figure S31: ^{1}H NMR spectra (400 MHz, buffered D_2O solution, pH 7.60, 298 K) of: (a) **PrS[6]$^{COO-}$**, (b) an equimolar solution of **PrS[6]$^{COO-}$** and **7^{2+} · 2I^{-}** and (c) **7^{2+} · 2I^{-}** .

6^+@ PrS[6]$^{COO-}$

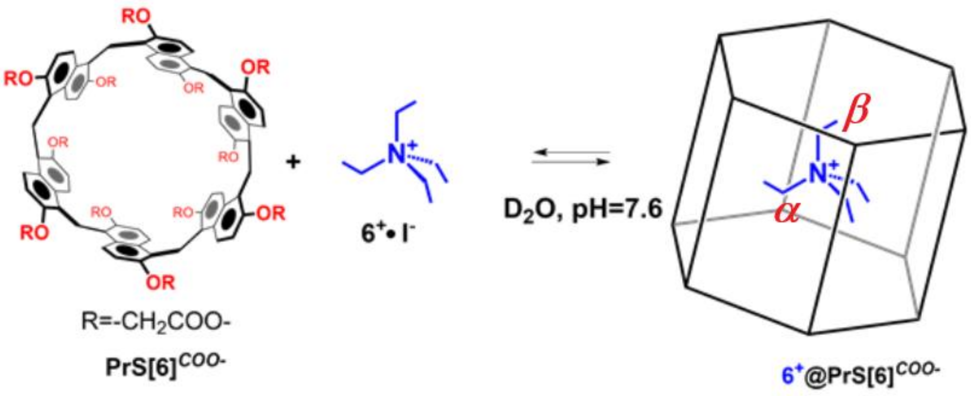

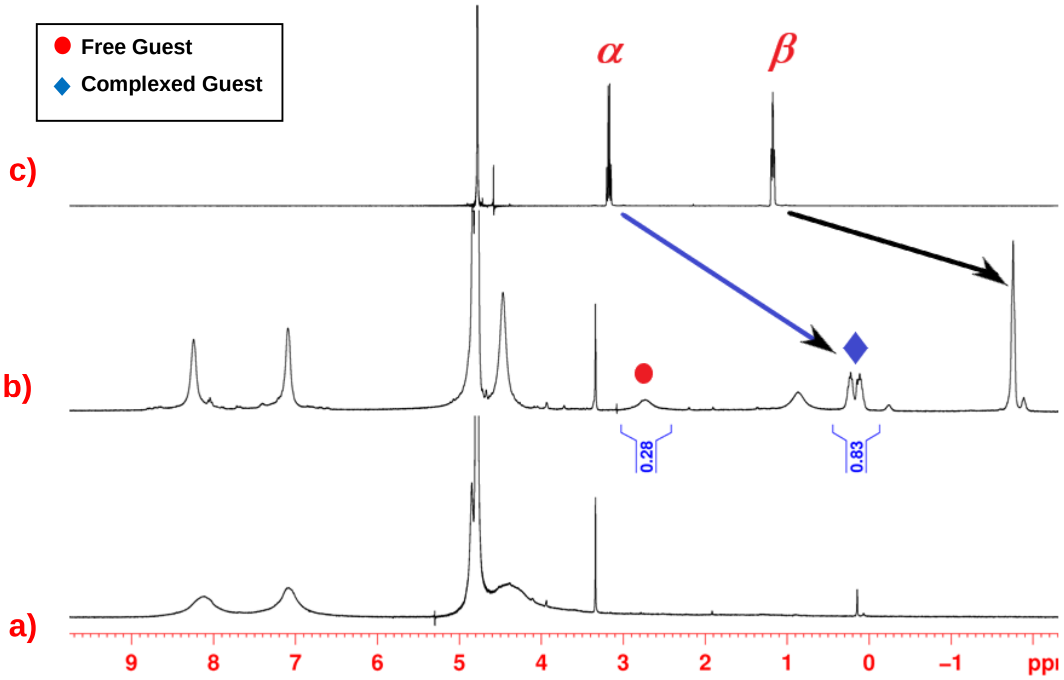

Figure S32: ^{1}H NMR spectra (400 MHz, buffered D_2O solution, pH 7.60, 298 K) of: (a) **PrS[6]$^{COO-}$**, (b) an equimolar solution of **PrS[6]$^{COO-}$** and **6^+ · I^-** and (c) **6^+ · I^-**.

The association constant value for the formation of the **6^+@PrS[6]$^{COO-}$** complex was calculated by following method: **[6^+@PrS[6]$^{COO-}$] =** [(0.83/1.11) x 0.0062 M] = $4.64{\cdot}10^{-3}$ M; **[PrS[6]$^{COO-}$]**$_{free}$= **[6^+]**$_{free}$ = 0.0016 M; K (**6^+@PrS[6]$^{COO-}$**) = $4.64{\cdot}10^{-3}/2.56{\cdot}10^{-6}$= **1815** M^{-1}

NMR-derived association constant K (**6^+@PrS[6]$^{COO-}$**) = **1815** M^{-1}

is in good agreement with the value obtained by ITC-derived measurement of

1990 M^{-1}

ITC titrations experiments

Experimental

Calorimetric titrations were carried out at 25 °C with a nano-isothermal titration calorimeter (Nano-ITC, TA Instruments, USA) having an active cell volume of 0.988 mL and a 250 μL injection syringe. Injection time intervals were chosen to guarantee equilibrium conditions before each subsequent addition. The reaction mixture in the sample cell was stirred at 250 rpm during the titration. The reference cell was always filled with ultrapure water. All solutions were stirred and degassed under vacuum for about 15 min before each run. Measurements were run in the overfilled mode. The power curve was integrated by NanoAnalyze (TA Instruments, USA) to obtain the gross heat of reaction. The calorimeter was calibrated chemically by a test HCl/TRIS reaction according to the procedure previously described.[5] The instrument was also checked through electrical calibrations.

ITC measurements were conducted by titrating an aqueous buffered solution of each guest (2.5–20.0 mM) into an aqueous buffered solution of **PrS[*n*]$^{COO-}$** (0.25–1.2 mM) host. All solutions were prepared in 70 mM phosphate buffer (pH 7.6). Typically, three independent experiments were run for each host-guest system. Heats of dilution were determined in separate "blank" experiments by titrating solutions of each guest into phosphate buffer (pH 7.6, 70 mM) only. The net heats of reaction (raw reaction heats - dilution heats) were obtained by subtracting the heat evolved/absorbed in the blank experiments. Net heat values obtained from different titrations were analyzed simultaneously by HypCal software,[6] which allows for the determination of both equilibrium constants and enthalpies of complex formation through a non-linear least-squares minimization procedure.

ITC Titrations of PrS[5]$^{COO-}$

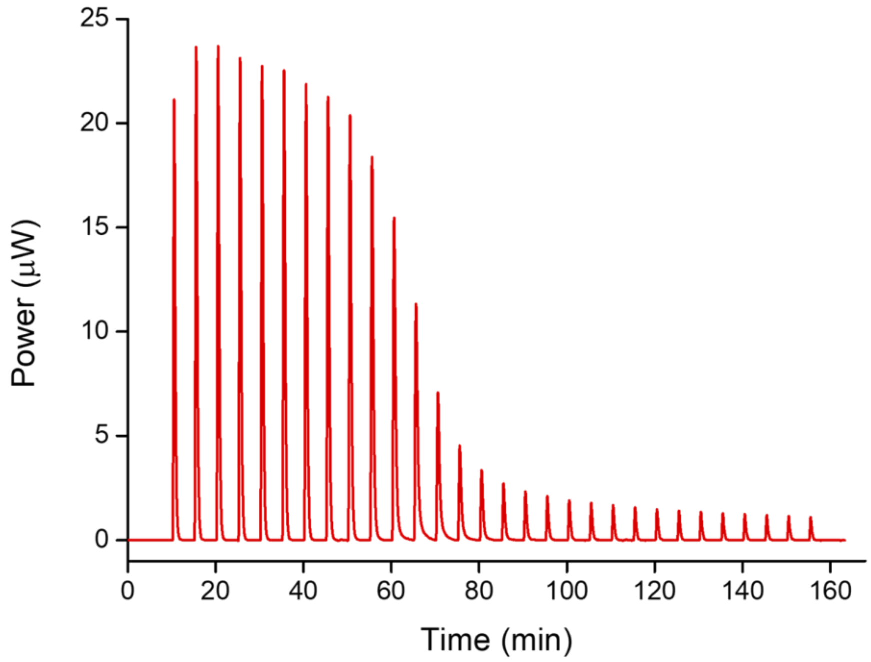

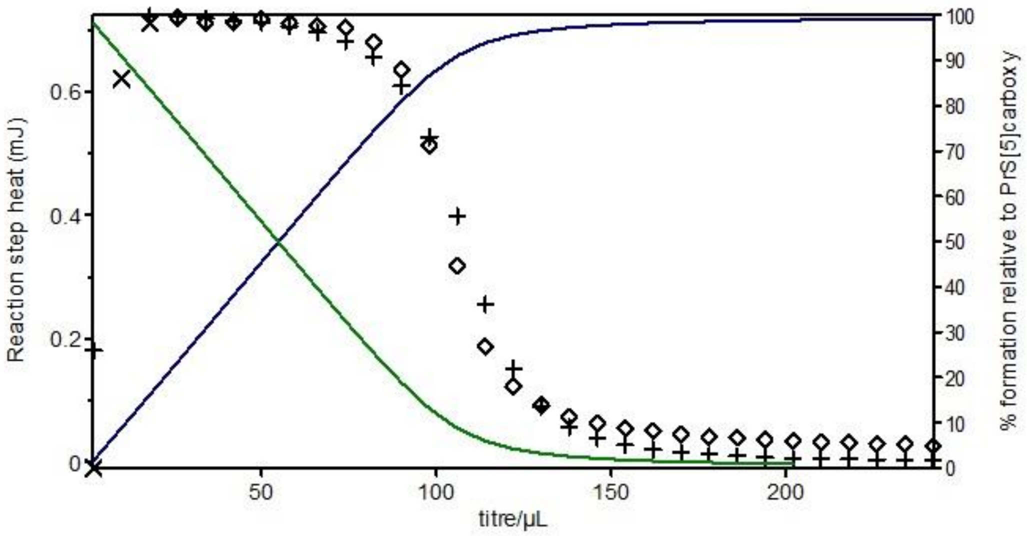

Figure S33: Typical ITC titration of **7**$^{2+}$ (2.51 mM) into a **PrS[5]**$^{COO-}$ (0.32 mM) solution at 25 °C and pH 7.6 (top) and HypCal output (◊: $Q_{obs.}$, +: $Q_{calc.}$) obtained from the analysis of the net heat values (raw reaction heat – dilution heat) for the formation of the **7**$^{2+}$**@PrS[5]**$^{COO-}$ complex (bottom). The species distribution diagram [complex (blue), free host (green)] is also calculated by the software.

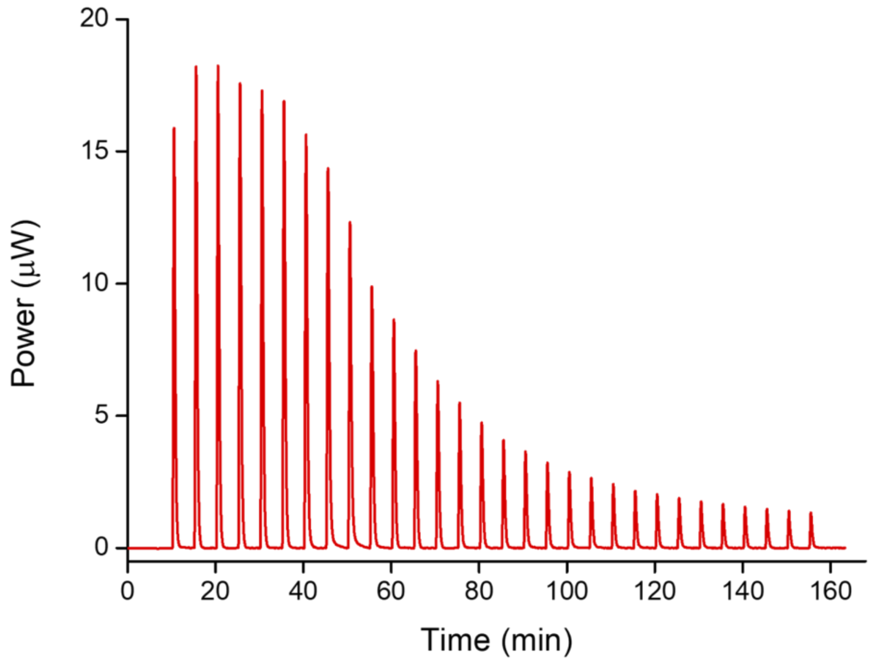

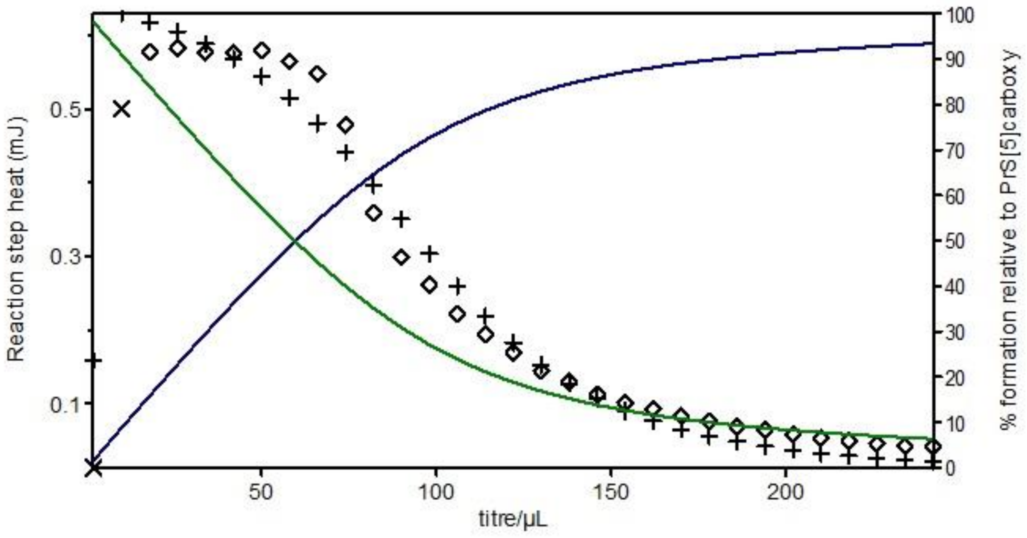

Figure S34: Typical ITC titration of **1^{2+}** (2.99 mM) into a **PrS[5]**$^{COO-}$ (0.33 mM) solution at 25 °C and pH 7.6 (top) and HypCal output (◊: $Q_{obs.}$, +: $Q_{calc.}$) obtained from the analysis of the net heat values (raw reaction heat – dilution heat) for the formation of the **1^{2+}@PrS[5]**$^{COO-}$ complex (bottom). The species distribution diagram [complex (blue), free host (green)] is also calculated by the software.

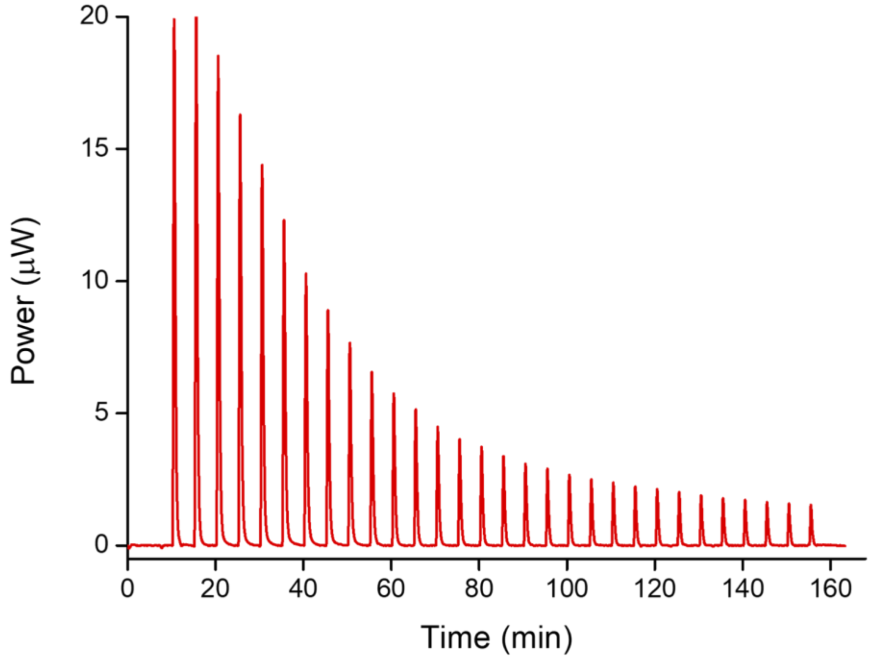

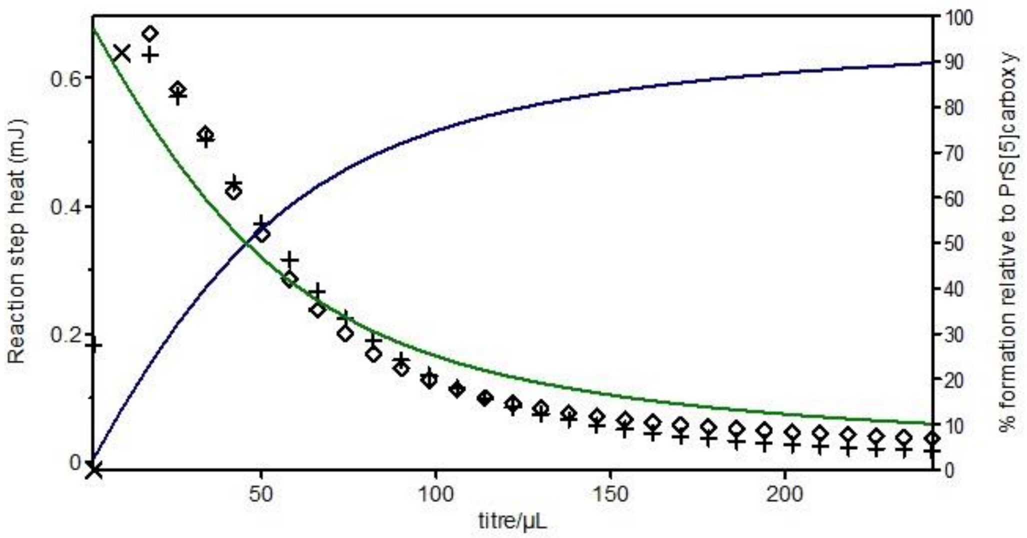

Figure S35: Typical ITC titration of **2**$^{2+}$ (6.45 mM) into a **PrS[5]**$^{COO-}$ (0.42 mM) solution at 25 °C and pH 7.6 (top) and HypCal output (◊: $Q_{obs.}$, +: $Q_{calc.}$) obtained from the analysis of the net heat values (raw reaction heat – dilution heat) for the formation of the **2**$^{2+}$**@PrS[5]**$^{COO-}$ complex (bottom). The species distribution diagram [complex (blue), free host (green)] is also calculated by the software.

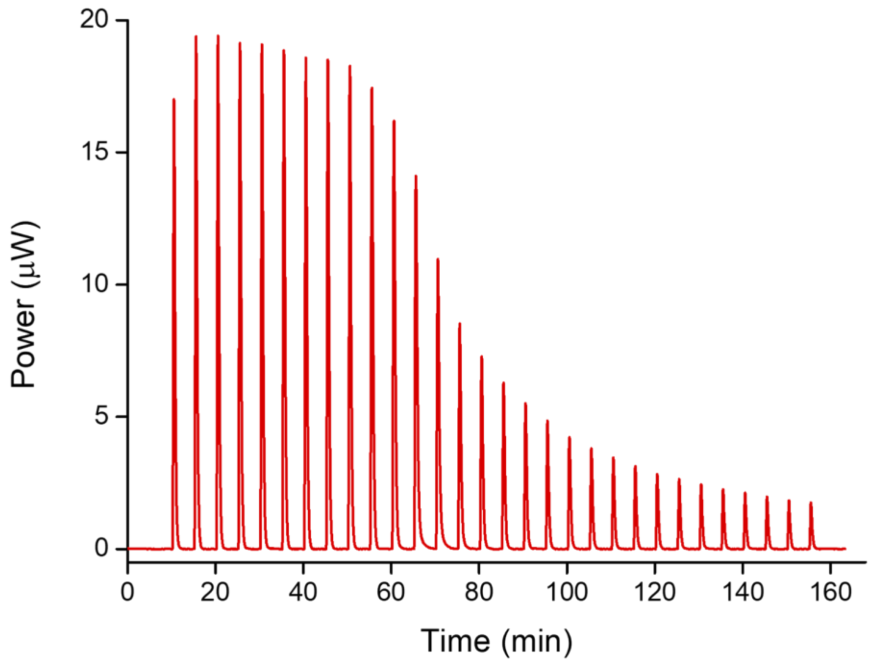

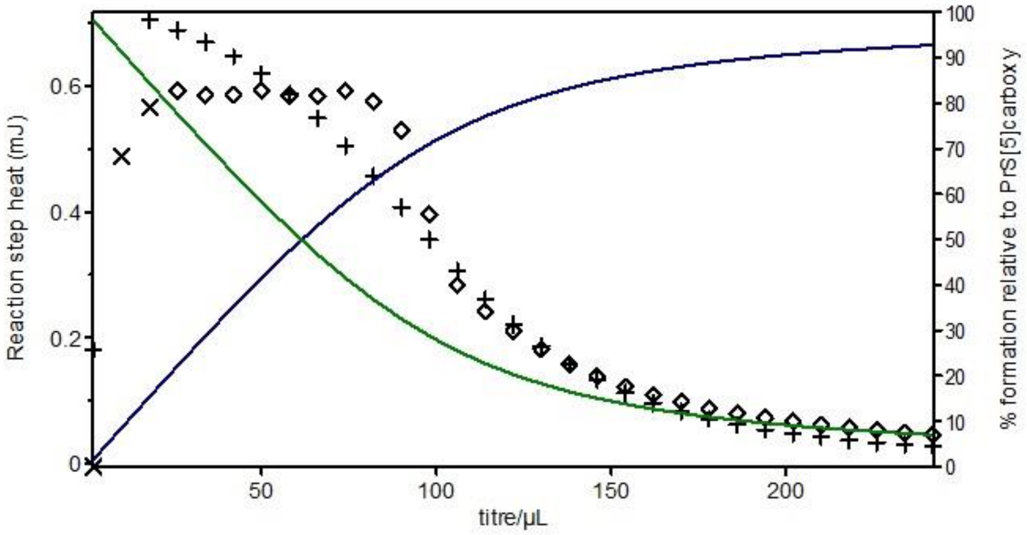

Figure S36: Typical ITC titration of **3**$^{2+}$ (2.99 mM) into a **PrS[5]**$^{COO-}$ (0.34 mM) solution at 25 °C and pH 7.6 (top) and HypCal output (◊: $Q_{obs.}$, +: $Q_{calc.}$) obtained from the analysis of the net heat values (raw reaction heat – dilution heat) for the formation of the **3**$^{2+}$**@PrS[5]**$^{COO-}$ complex (bottom). The species distribution diagram [complex (blue), free host (green)] is also calculated by the software.

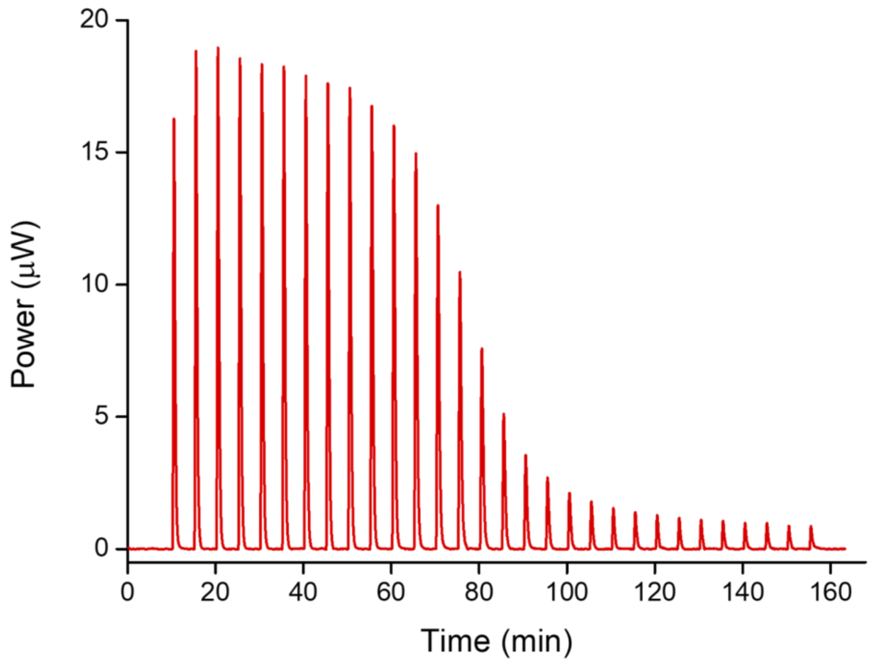

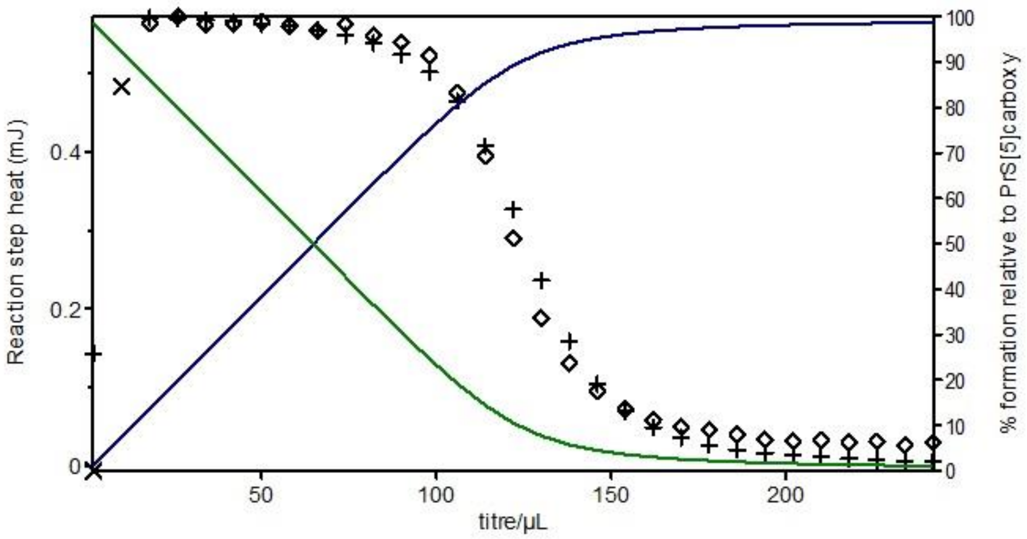

Figure S37: Typical ITC titration of $\mathbf{4^{2+}}$ (3.04 mM) into a **PrS[5]**$^{COO-}$ (0.35 mM) solution at 25 °C and pH 7.6 (top) and HypCal output (◊: $Q_{obs.}$, +: $Q_{calc.}$) obtained from the analysis of the net heat values (raw reaction heat – dilution heat) for the formation of the $\mathbf{4^{2+}}$**@PrS[5]**$^{COO-}$ complex (bottom). The species distribution diagram [complex (blue), free host (green)] is also calculated by the software.

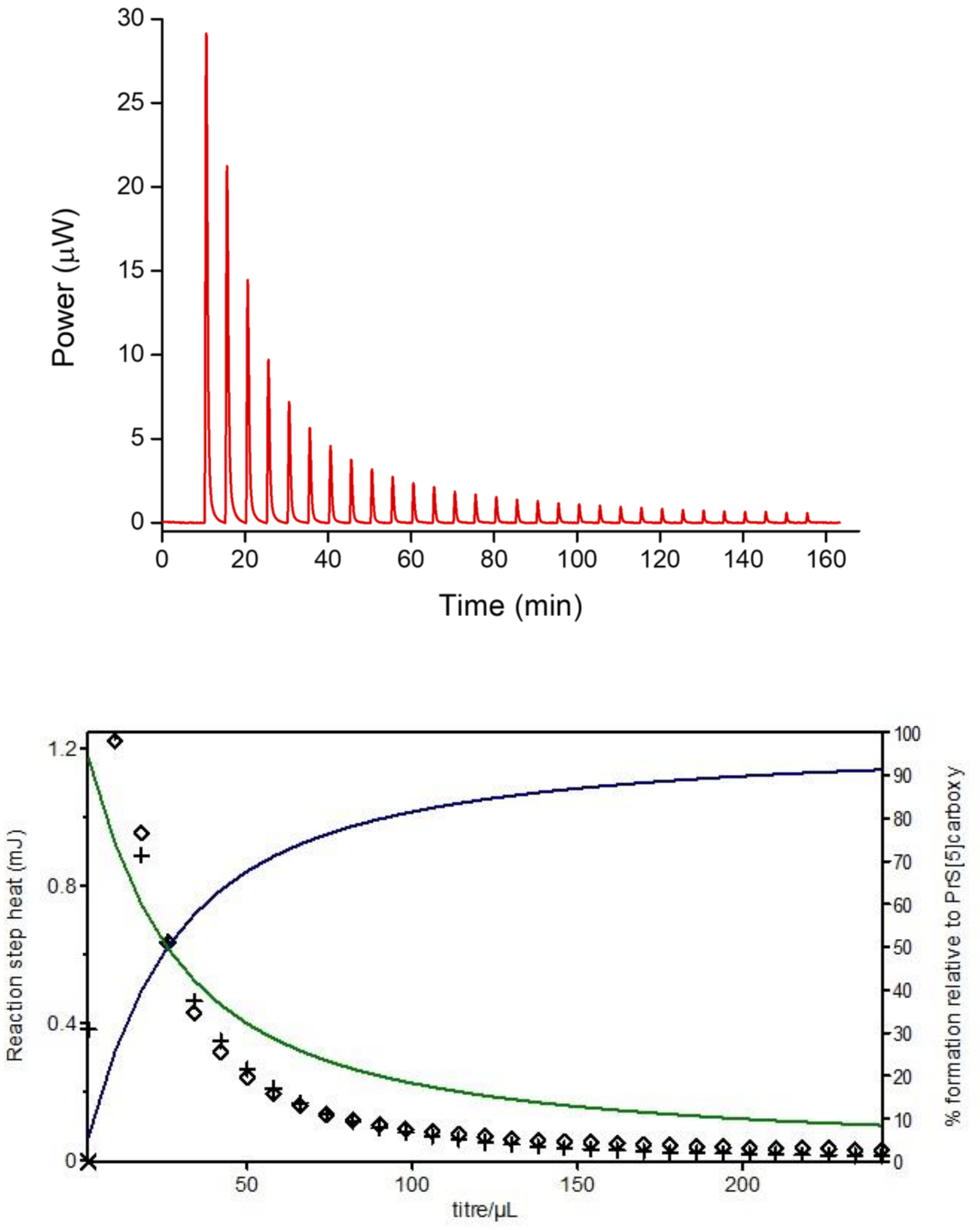

Figure S38: Typical ITC titration of **6**$^{+}$ (19.68 mM) into a **PrS[5]**$^{COO-}$ (0.26 mM) solution at 25 °C and pH 7.6 (top) and HypCal output (◊: $Q_{obs.}$, +: $Q_{calc.}$) obtained from the analysis of the net heat values (raw reaction heat – dilution heat) for the formation of the **6**$^{+}$**@PrS[5]**$^{COO-}$ complex (bottom). The species distribution diagram [complex (blue), free host (green)] is also calculated by the software.

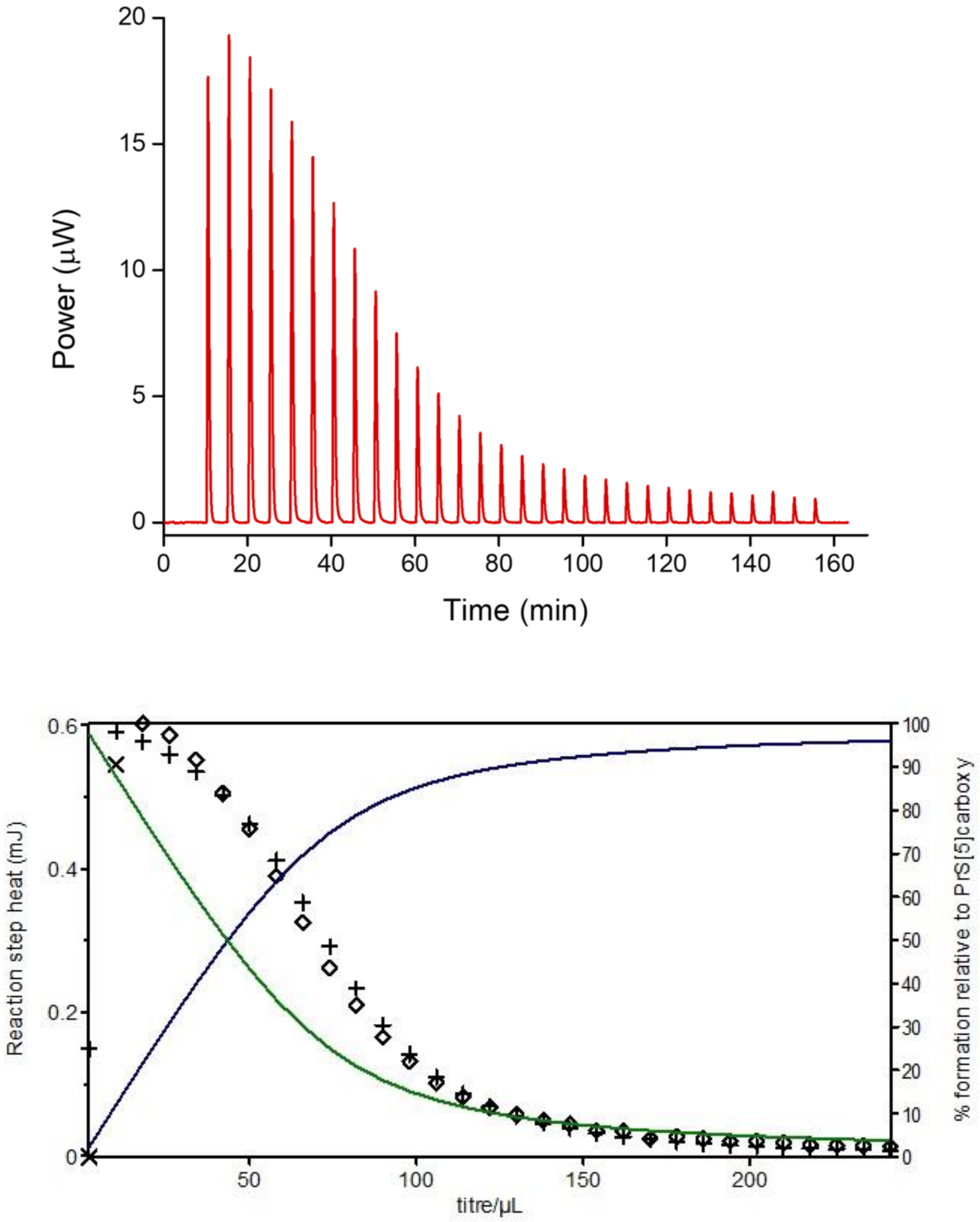

Figure S39: Typical ITC titration of **5^+** (5.03 mM) into a **PrS[5]**$^{COO^-}$ (0.47 mM) solution at 25 °C and pH 7.6 (top) and HypCal output (◊: $Q_{obs.}$, +: $Q_{calc.}$) obtained from the analysis of the net heat values (raw reaction heat – dilution heat) for the formation of the **5^+@PrS[5]**$^{COO^-}$ complex (bottom). The species distribution diagram [complex (blue), free host (green)] is also calculated by the software.

ITC Titrations of PrS[6]$^{COO-}$

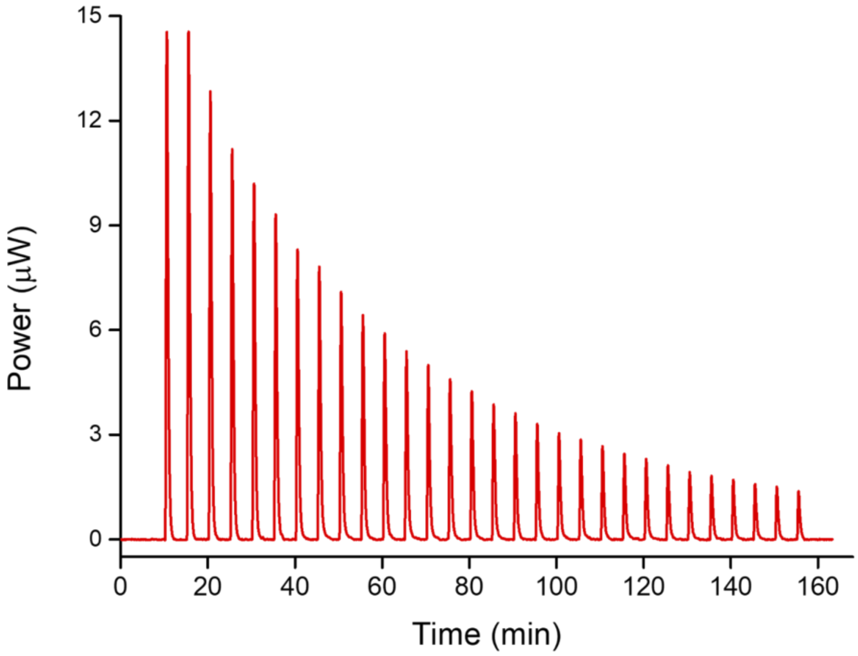

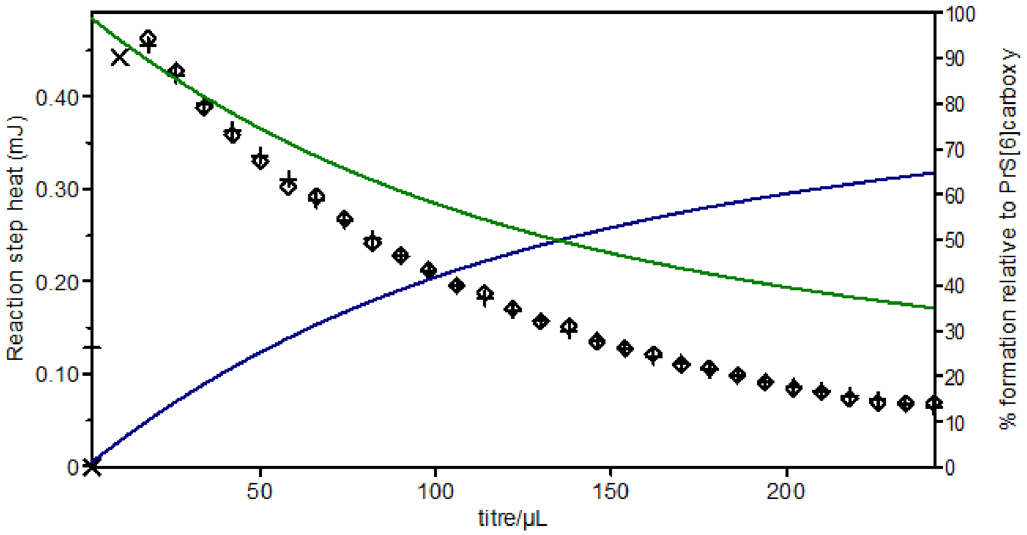

Figure S40: Typical ITC titration of **7**$^{2+}$ (11.0 mM) into a **PrS[6]**$^{COO-}$ (0.63 mM) solution at 25 °C and pH 7.6 (top) and HypCal output (◊: $Q_{obs.}$, +: $Q_{calc.}$) obtained from the analysis of the net heat values (raw reaction heat – dilution heat) for the formation of the **7**$^{2+}$**@PrS[6]**$^{COO-}$ complex (bottom). The species distribution diagram [complex (blue), free host (green)] is also calculated by the software.

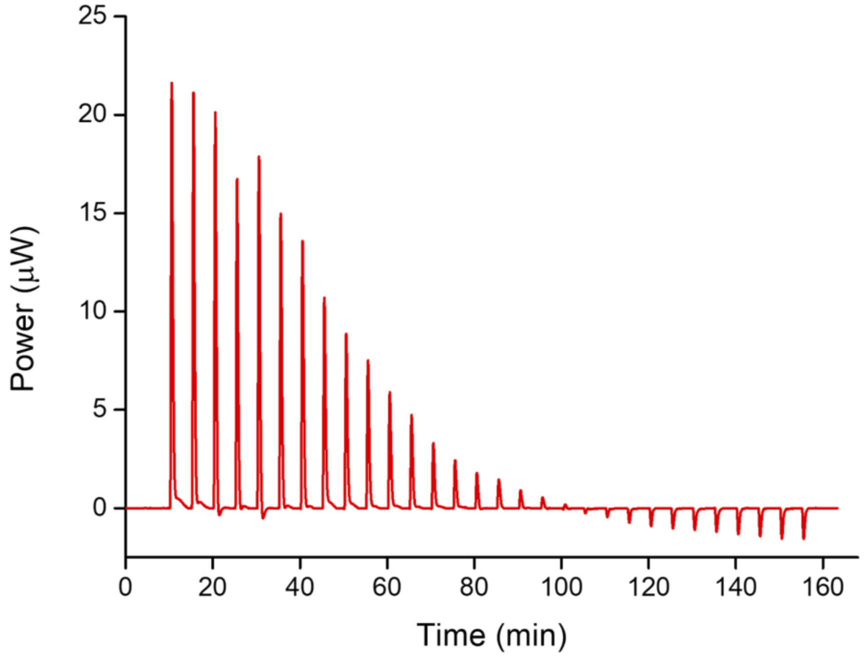

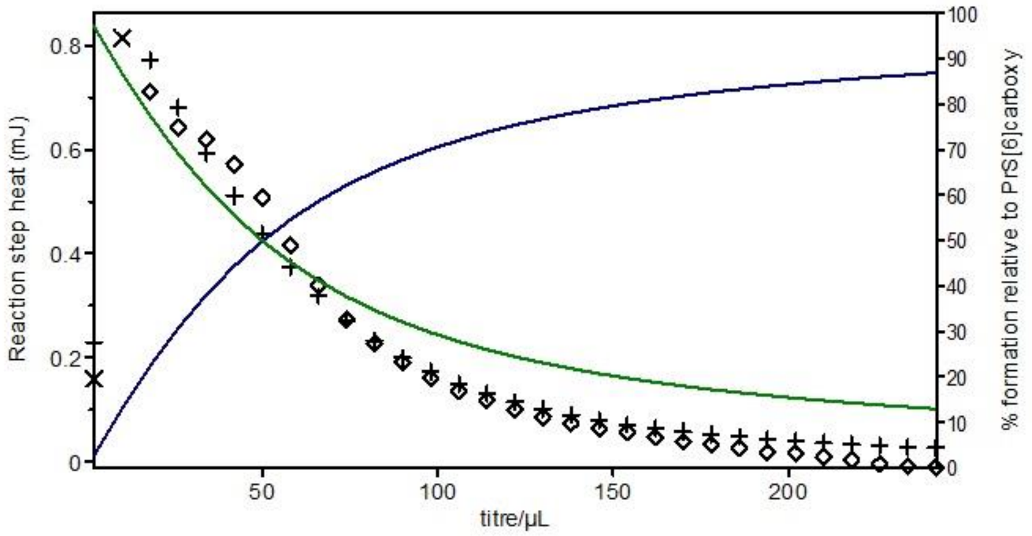

Figure S41: Typical ITC titration of **1**$^{2+}$ (14.34 mM) into a **PrS[6]**$^{COO-}$ (0.98 mM) solution at 25 °C and pH 7.6 (top) and HypCal output (◊: $Q_{obs.}$, +: $Q_{calc.}$) obtained from the analysis of the net heat values (raw reaction heat – dilution heat) for the formation of the **1**$^{2+}$**@PrS[6]**$^{COO-}$ complex (bottom). The species distribution diagram [complex (blue), free host (green)] is also calculated by the software.

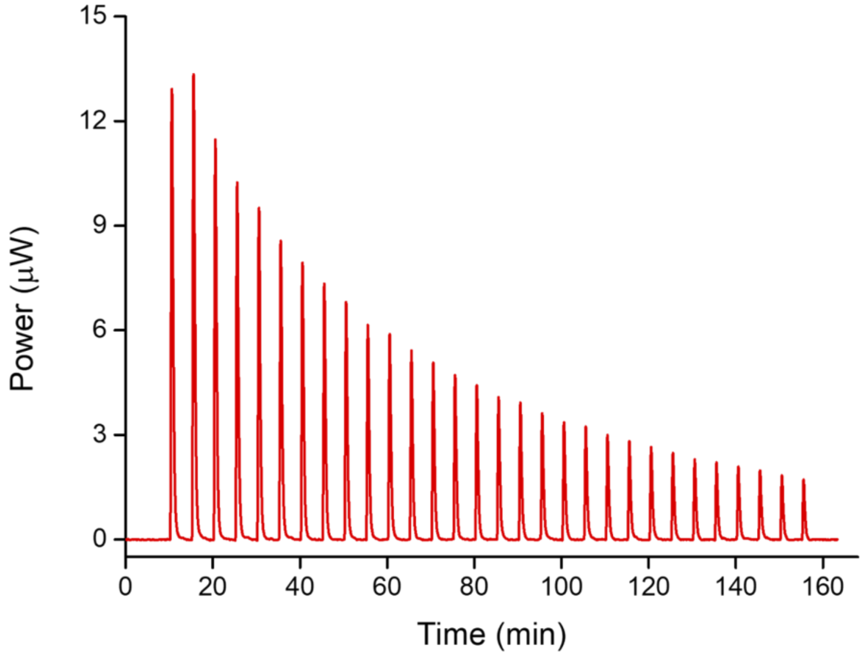

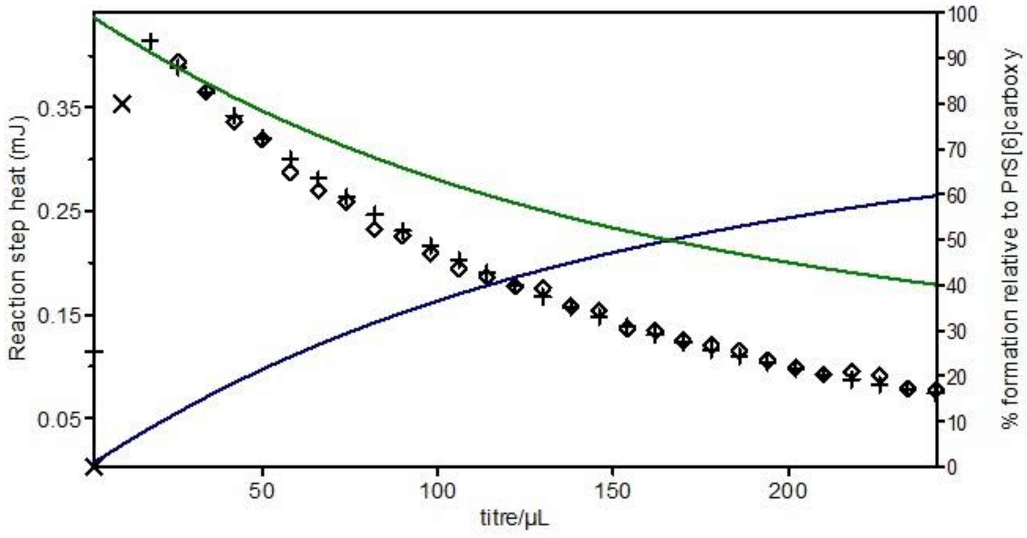

Figure S42: Typical ITC titration of **2**$^{2+}$ (9.65 mM) into a **PrS[6]**$^{COO-}$ (0.75 mM) solution at 25 °C and pH 7.6 (top) and HypCal output (◊: $Q_{obs.}$, +: $Q_{calc.}$) obtained from the analysis of the net heat values (raw reaction heat – dilution heat) for the formation of the **2**$^{2+}$**@PrS[6]**$^{COO-}$ complex (bottom). The species distribution diagram [complex (blue), free host (green)] is also calculated by the software.

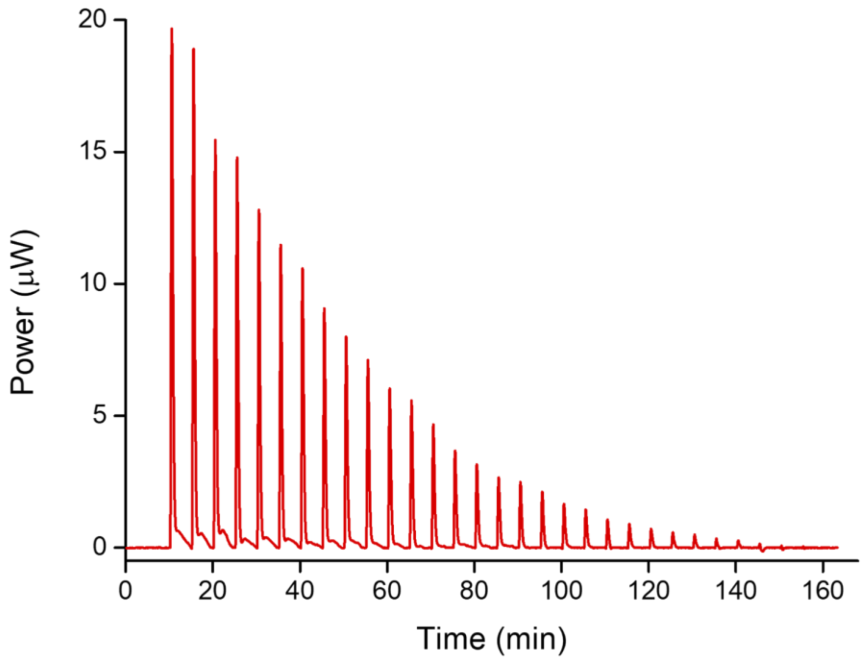

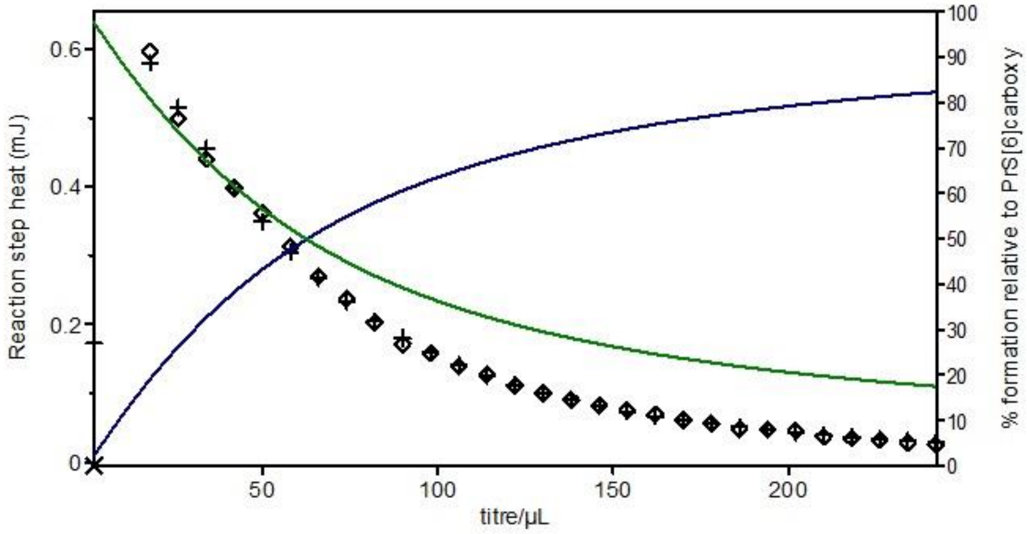

Figure S43: Typical ITC titration of **3**$^{2+}$ (12.92 mM) into a **PrS[6]**$^{COO-}$ (0.82 mM) solution at 25 °C and pH 7.6 (top) and HypCal output (◊: $Q_{obs.}$, +: $Q_{calc.}$) obtained from the analysis of the net heat values (raw reaction heat – dilution heat) for the formation of the **3**$^{2+}$**@PrS[6]**$^{COO-}$ complex (bottom). The species distribution diagram [complex (blue), free host (green)] is also calculated by the software.

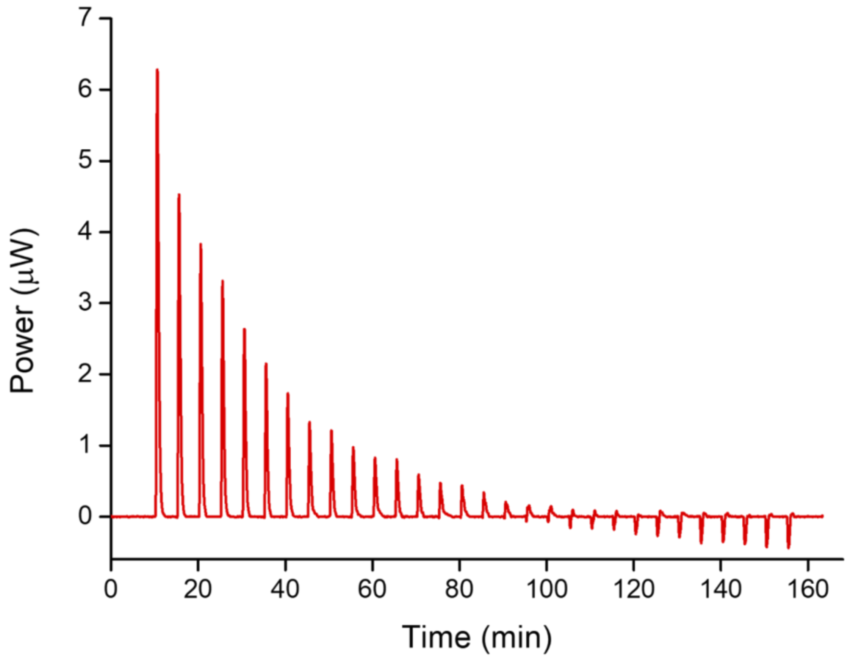

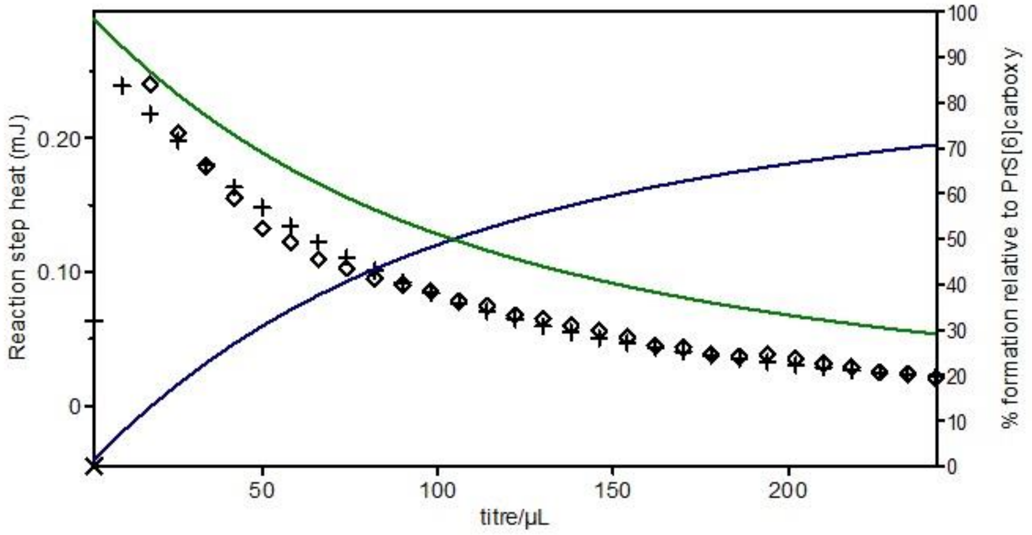

Figure S44: Typical ITC titration of **4^{2+}** (10.5 mM) into a **PrS[6]$^{COO-}$** (0.51 mM) solution at 25 °C and pH 7.6 (top) and HypCal output (◊: $Q_{obs.}$, +: $Q_{calc.}$) obtained from the analysis of the net heat values (raw reaction heat – dilution heat) for the formation of the **4^{2+}@PrS[6]$^{COO-}$** complex (bottom). The species distribution diagram [complex (blue), free host (green)] is also calculated by the software.

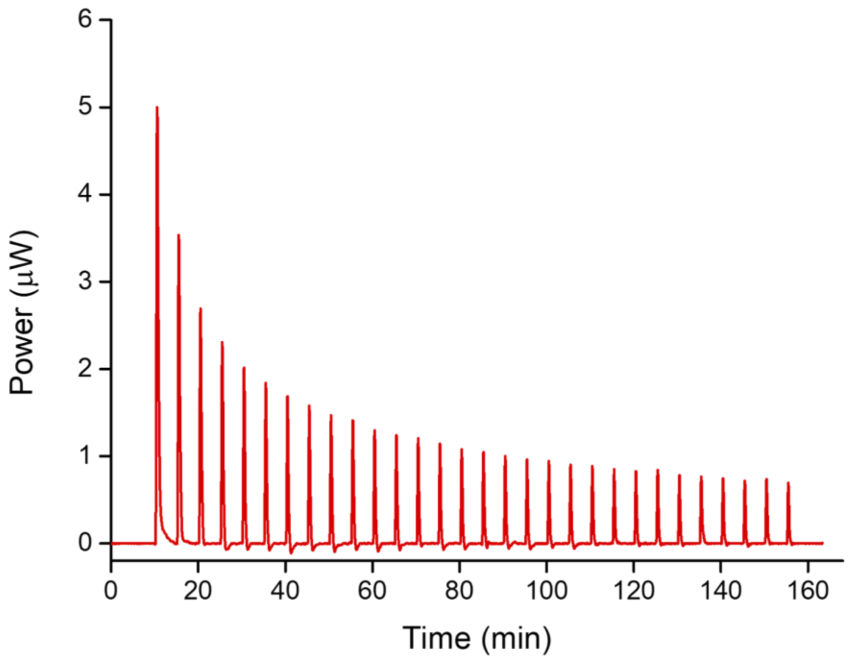

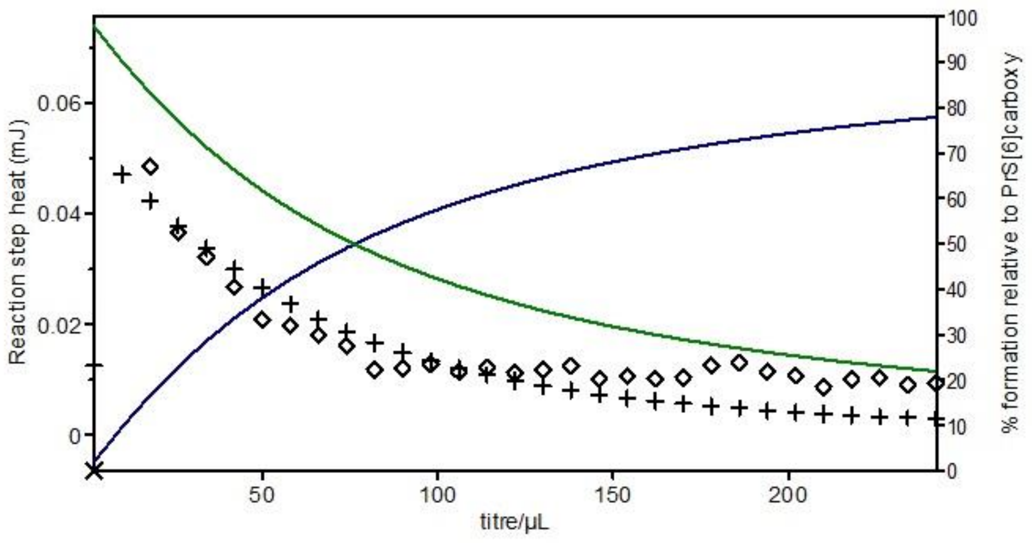

Figure S45: Typical ITC titration of **6⁺** (19.68 mM) into a **PrS[6]**$^{COO-}$ (0.75 mM) solution at 25 °C and pH 7.6 (top) and HypCal output (◊: $Q_{obs.}$, +: $Q_{calc.}$) obtained from the analysis of the net heat values (raw reaction heat – dilution heat) for the formation of the **6⁺@PrS[6]**$^{COO-}$ complex (bottom). The species distribution diagram [complex (blue), free host (green)] is also calculated by the software.

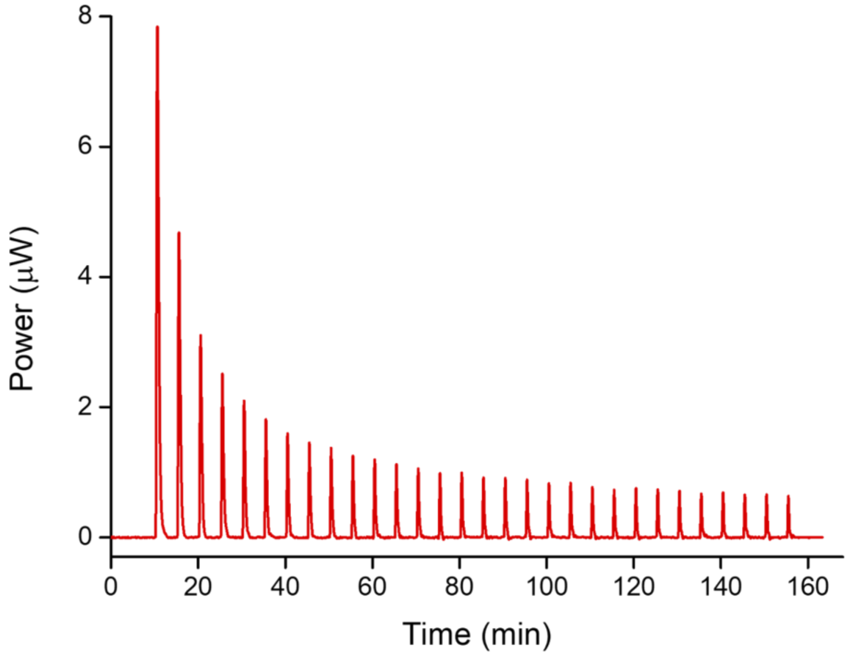

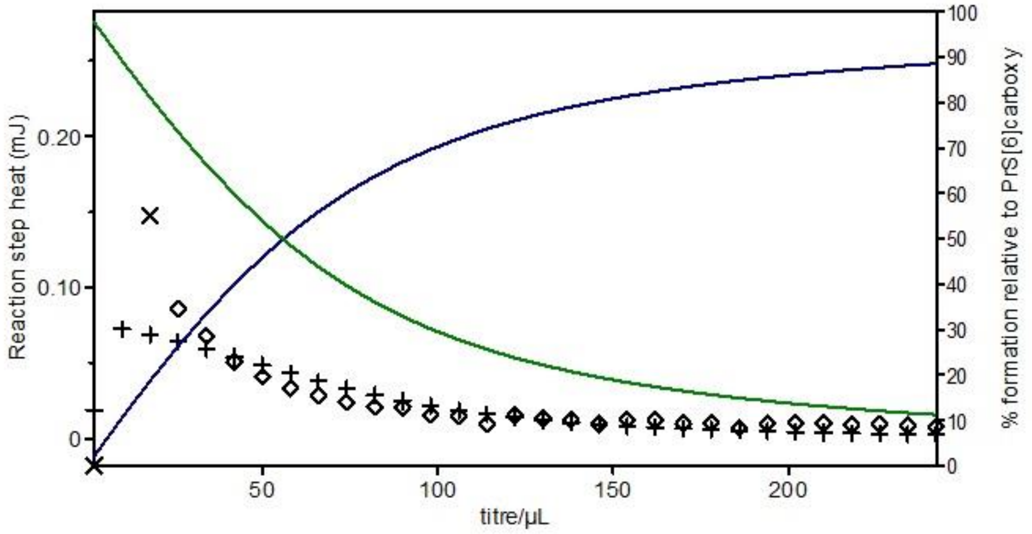

Figure S46: Typical ITC titration of **5⁺** (11.0 mM) into a **PrS[6]**$^{COO-}$ (0.75 mM) solution at 25 °C and pH 7.6 (top) and HypCal output (◊: $Q_{obs.}$, +: $Q_{calc.}$) obtained from the analysis of the net heat values (raw reaction heat – dilution heat) for the formation of the **5⁺@PrS[6]**$^{COO-}$ complex (bottom). The species distribution diagram [complex (blue), free host (green)] is also calculated by the software.

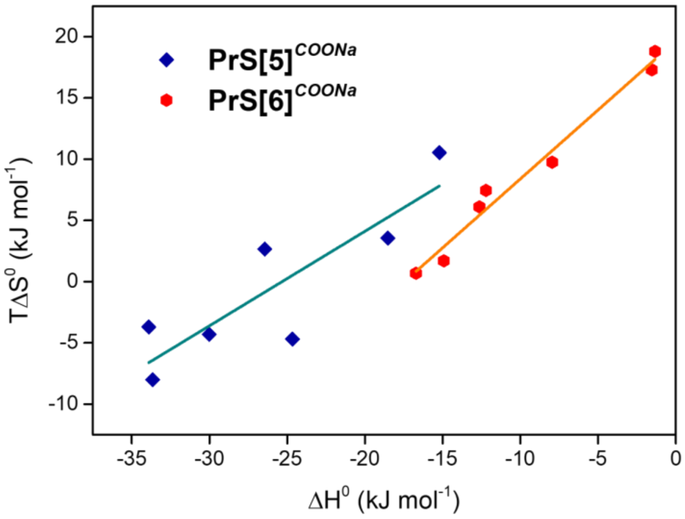

Figure S47. Enthalpy-entropy compensation plot for **PrS[n]**$^{\mathbf{COO^–}}$ - guest complex formation at 25 °C in buffered aqueous solution (pH 7.6).

DFT optimized structure of 7^{2+}@ PrS[5]$^{COO-}$ complex

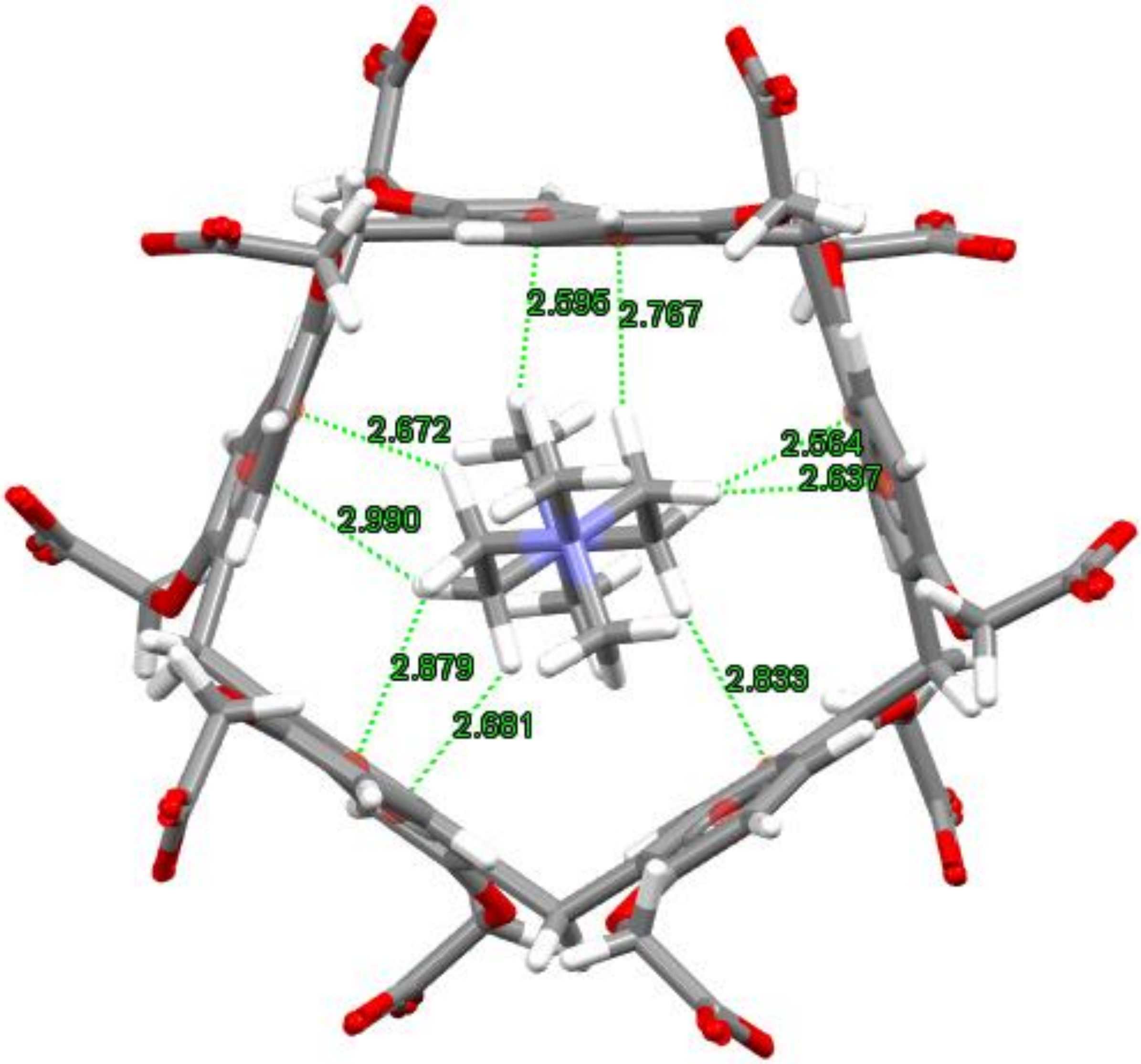

Figure S48. DFT-optimized structure of the complex **7**$^{2+}$@**PrS[5]**$^{COO–}$ at B97D3/SVP/SVPFIT level of theory. Highlighted C–H···π interactions between the guest **7**$^{2+}$ and the aromatic walls of **PrS[5]**$^{COO–}$

C–H···π^{centroid} distance of 2.73 Å

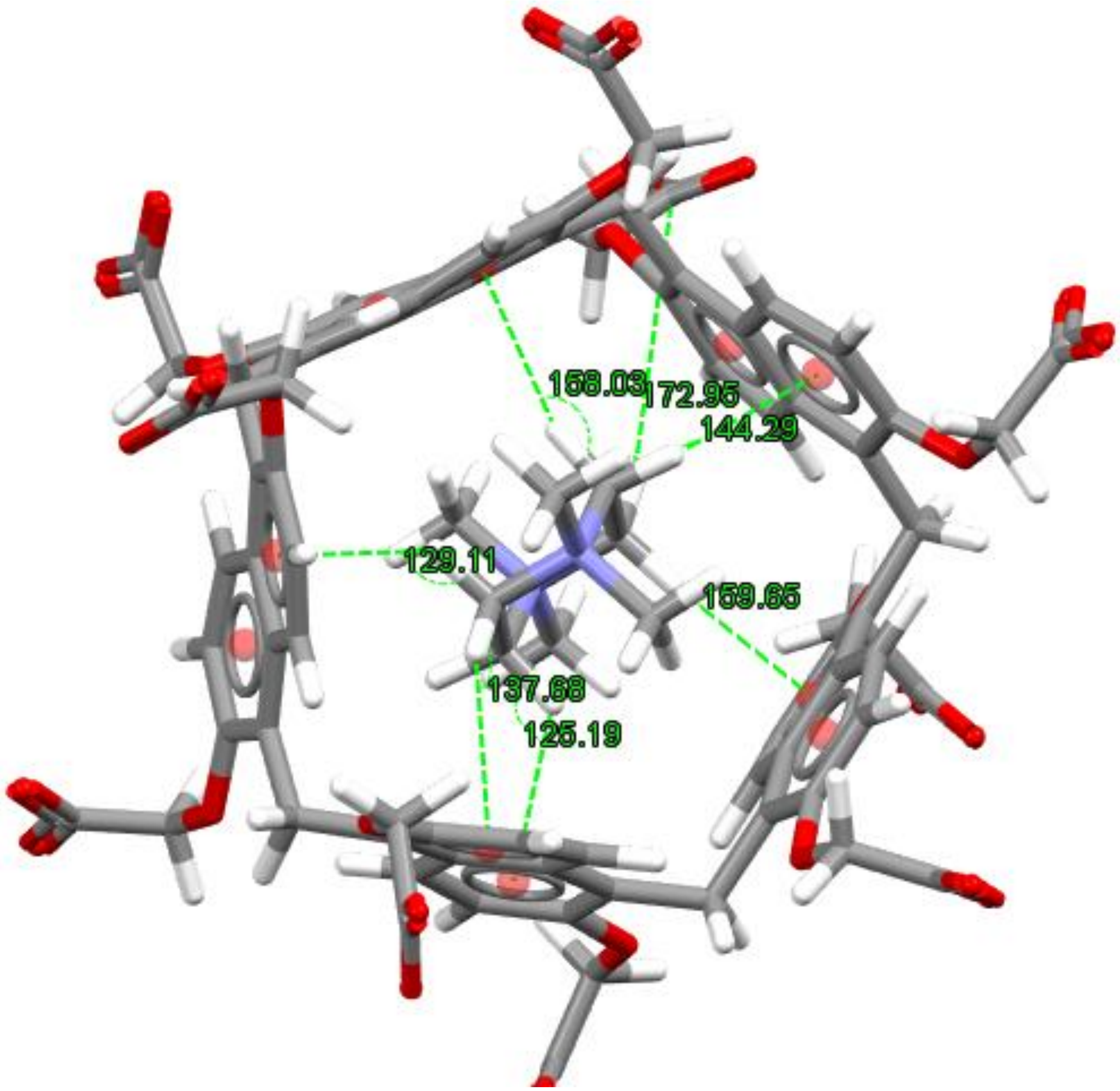

Figure S49. DFT-optimized structure of the complex **7**$^{2+}$@**PrS[5]**$^{COO-}$ at B97D3/SVP/SVPFIT level of theory. Highlighted C–H···π^{centroid} angles.

Atomic coordinates of **7**$^{2+}$ **@PrS]5]**$^{COO-}$ complex.

C1	2.3988	3.7980	-0.2541	C
C2	1.1453	4.2851	0.2945	C
C3	0.0950	4.7791	-0.5629	C
C4	0.3562	4.9149	-1.9427	C
C5	1.5841	4.4328	-2.4808	C
C6	2.5507	3.8573	-1.6733	C
C7	3.4850	3.3969	0.6060	C
C8	3.3729	3.6284	1.9930	C
C9	2.1420	4.0996	2.5316	C
C10	1.0546	4.3759	1.7178	C
O11	-0.6043	5.4890	-2.7120	O
O12	4.4579	3.3649	2.7656	O
C13	0.3616	-4.4165	-0.2941	C
C14	1.6817	-4.1559	0.2528	C
C15	2.8187	-3.9436	-0.6089	C
C16	2.6646	-4.1438	-1.9968	C
C17	1.3692	-4.3903	-2.5338	C
C18	0.2527	-4.4806	-1.7176	C
C19	-0.7562	-4.7265	0.5641	C
C20	-0.5198	-4.9112	1.9428	C
C21	0.7742	-4.6514	2.4792	C
C22	1.8240	-4.2480	1.6712	C
O23	3.7770	-4.0698	-2.7717	O
O24	-1.5631	-5.3146	2.7125	O
C25	-2.1473	-4.9987	-0.0099	C
C26	-2.9495	3.3728	-0.2733	C
C27	-3.8718	2.3960	0.2764	C
C28	-4.6856	1.5708	-0.5795	C
C29	-4.6913	1.8302	-1.9660	C
C30	-3.7736	2.7763	-2.5083	C
C31	-2.9075	3.4921	-1.6960	C
C32	-2.1938	4.2514	0.5866	C
C33	-2.4619	4.2369	1.9724	C
C34	-3.3506	3.2634	2.5118	C
C35	-4.0058	2.3558	1.6973	C
O36	-5.5689	1.1338	-2.7330	O
O37	-1.8371	5.1608	2.7463	O
C38	-1.2273	5.2882	0.0125	C
C39	4.4570	-1.0592	-0.2858	C
C40	4.5779	0.2718	0.2806	C
C41	4.7172	1.4325	-0.5624	C
C42	4.8873	1.2467	-1.9501	C
C43	4.7504	-0.0572	-2.5081	C
C44	4.5079	-1.1636	-1.7093	C
C45	4.3804	-2.2260	0.5575	C
C46	4.5765	-2.0727	1.9458	C
C47	4.6756	-0.7652	2.5037	C
C48	4.6413	0.3663	1.7042	C
O49	5.1549	2.3432	-2.7053	O
O50	4.6423	-3.1988	2.7019	O
C51	4.2001	-3.6233	-0.0364	C
C52	-4.2413	-1.6866	-0.2709	C
C53	-3.5059	-2.8113	0.2778	C
C54	-2.9189	-3.8096	-0.5832	C
C55	-3.1838	-3.7493	-1.9681	C
C56	-3.8871	-2.6340	-2.5065	C
C57	-4.3682	-1.6237	-1.6916	C
C58	-4.8929	-0.7295	0.5863	C
C59	-4.9432	-0.9843	1.9727	C
C60	-4.2084	-2.0791	2.5137	C
C61	-3.4838	-2.9368	1.7004	C
O62	-2.7322	-4.7694	-2.7424	O
O63	-5.6812	-0.1426	2.7411	O
C64	-5.6013	0.4942	0.0042	C
C65	4.7877	2.8392	0.0316	C
H66	1.7859	4.5554	-3.5479	H
H67	3.4771	3.5090	-2.1344	H
H68	2.0641	4.2887	3.6053	H
H69	0.1392	4.7518	2.1795	H
C70	-0.4470	5.7069	-4.1266	C
C71	4.4759	3.5759	4.1893	C
H72	1.2571	-4.5563	-3.6084	H
H73	-0.7145	-4.6919	-2.1784	H
H74	0.9541	-4.8131	3.5450	H
H75	2.7977	-4.0671	2.1311	H
C76	3.7546	-4.2749	-4.1963	C
C77	-1.4436	-5.5563	4.1268	C
H78	-2.7457	-5.4602	0.7907	H
H79	-2.0618	-5.7496	-0.8109	H
H80	-3.7857	2.9790	-3.5824	H
H81	-2.2327	4.2151	-2.1599	H
H82	-3.5630	3.2672	3.5838	H
H83	-4.6912	1.6423	2.1586	H
C84	-5.6844	1.3177	-4.1560	C
C85	-2.0432	5.2578	4.1682	C
H86	-1.7370	5.8469	-0.7875	H
H87	-1.0119	6.0124	0.8136	H
H88	4.8880	-0.1974	-3.5834	H
H89	4.4211	-2.1422	-2.1854	H
H90	4.8331	-0.6521	3.5794	H
H91	4.7318	1.3447	2.1802	H
C92	5.3731	2.2811	-4.1267	C
C93	4.8638	-3.1754	4.1240	C
H94	4.9336	-3.7738	-0.8434	H
H95	4.4447	-4.3513	0.7525	H
H96	-4.0984	-2.6002	-3.5783	H
H97	-4.9098	-0.8082	-2.1477	H
H98	-4.2551	-2.2766	3.5879	H
H99	-2.9474	-3.7683	2.1632	H
C100	-2.9611	-4.8335	-4.1626	C
C101	-5.8276	-0.3048	4.1640	C

H102	-6.2912	0.1763	-0.7926	H
H103	-6.2207	0.9314	0.8024	H
H104	5.1556	3.5130	-0.7576	H
H105	5.5375	2.8582	0.8375	H
C106	-6.9232	-1.3329	4.6693	C
O107	-7.4336	-2.0967	3.8230	O
O108	-7.1284	-1.2222	5.9089	O
C109	-4.3718	-5.3665	-4.6511	C
H110	-2.7578	-3.8500	-4.6351	H
H111	-2.1866	-5.5301	-4.5292	H
O112	-4.3793	-5.5914	-5.8919	O
O113	-5.2694	-5.4962	-3.7923	O
C114	-0.8471	-6.9526	4.5809	C
H115	-0.8728	-4.7389	4.6144	H
H116	-2.4806	-5.4894	4.5007	H
O117	-1.0088	-7.1482	5.8163	O
O118	-0.3097	-7.6635	3.7057	O
C119	3.7774	-5.7720	-4.7168	C
H120	2.8968	-3.7404	-4.6546	H
H121	4.6708	-3.7743	-4.5564	H
O122	3.9922	-5.8296	-5.9582	O
O123	3.5897	-6.6783	-3.8781	O
C124	6.3596	-3.0221	4.6256	C
H125	4.2344	-2.3981	4.6048	H
H126	4.4851	-4.1524	4.4731	H
O127	7.2235	-2.7075	3.7800	O
O128	6.4619	-3.2507	5.8617	O
C129	6.8199	1.8666	-4.6242	C
H130	4.6177	1.6275	-4.6100	H
H131	5.1740	3.3097	-4.4764	H
O132	6.9635	2.0717	-5.8605	O
O133	7.6137	1.4073	-3.7762	O
H134	5.2927	2.9255	4.5492	H
C135	4.7589	5.0483	4.7033	C
H136	3.5399	3.1996	4.6519	H
O137	4.9883	5.0725	5.9433	O
O138	4.7237	5.9707	3.8618	O
C139	0.3798	6.9801	-4.5822	C
H140	-0.0269	4.8037	-4.6158	H
H141	-1.4807	5.8200	-4.4984	H
O142	0.2578	7.1956	-5.8188	O
O143	1.0261	7.5932	-3.7067	O
H144	-1.1571	5.8070	4.5324	H
C145	-3.3369	6.0266	4.6664	C
H146	-2.0120	4.2516	4.6354	H
O147	-4.2039	6.3111	3.8134	O
O148	-3.2977	6.2474	5.9072	O
C149	-6.5770	2.5258	-4.6626	C
H150	-4.6808	1.3806	-4.6251	H
H151	-6.1447	0.3798	-4.5142	H
O152	-6.9387	3.3715	-3.8175	O
O153	-6.8013	2.4511	-5.9015	O
H154	-6.1112	0.7002	4.5238	H
H155	-4.8516	-0.5481	4.6323	H
N156	-0.0737	-0.0492	-1.5003	N
C157	-1.3245	-0.2148	-0.6508	C
C158	-1.2243	0.4956	0.6804	C
N159	-0.0154	0.0535	1.4907	N
C160	1.2385	0.2178	0.6423	C
C161	1.1377	-0.4921	-0.6905	C
C162	0.0754	1.3807	-1.9635	C
C163	-0.1969	-0.9548	-2.6961	C
H164	-1.4996	-1.2958	-0.5387	H
H165	-2.1814	0.2024	-1.2025	H
H166	-2.1345	0.2751	1.2594	H
H167	-1.1621	1.5897	0.5713	H
C168	0.1072	0.9591	2.6866	C
C169	-0.1732	-1.3754	1.9547	C
H170	1.4187	1.2989	0.5285	H
H171	2.0905	-0.1998	1.2010	H
H172	1.0745	-1.5861	-0.5772	H
H173	2.0456	-0.2746	-1.2753	H
H174	0.7073	-0.8358	-3.3101	H
H175	-0.2756	-1.9971	-2.3502	H
H176	-1.1009	-0.6696	-3.2527	H
H177	0.9853	1.4582	-2.5726	H
H178	-0.8226	1.6546	-2.5349	H
H179	0.1652	2.0614	-1.1079	H
H180	0.9852	0.6413	3.2673	H
H181	0.2470	1.9953	2.3409	H
H182	-0.8161	0.8767	3.2775	H
H183	0.7149	-1.6555	2.5360	H
H184	-1.0914	-1.4447	2.5547	H
H185	-0.2626	-2.0576	1.1002	H

1 1 2 Ar
2 1 6 Ar
3 1 7 Ar
4 2 3 Ar
5 2 10 Ar
6 3 4 Ar
7 3 38 1
8 4 5 Ar
9 4 11 1
10 5 6 Ar
11 5 66 1
12 6 67 1
13 7 8 Ar
14 7 65 1
15 8 9 Ar
16 8 12 1
17 9 10 Ar
18 9 68 1
19 10 69 1

20 11 70 1
21 12 71 1
22 13 14 Ar
23 13 18 Ar
24 13 19 Ar
25 14 15 Ar
26 14 22 Ar
27 15 16 Ar
28 15 51 1
29 16 17 Ar
30 16 23 1
31 17 18 Ar
32 17 72 1
33 18 73 1
34 19 20 Ar
35 19 25 1
36 20 21 Ar
37 20 24 1
38 21 22 Ar
39 21 74 1
40 22 75 1
41 23 76 1
42 24 77 1
43 25 54 1
44 25 78 1
45 25 79 1
46 26 27 Ar
47 26 31 Ar
48 26 32 Ar
49 27 28 Ar
50 27 35 Ar
51 28 29 Ar
52 28 64 1
53 29 30 Ar
54 29 36 1
55 30 31 Ar
56 30 80 1
57 31 81 1
58 32 33 Ar
59 32 38 1
60 33 34 Ar
61 33 37 1
62 34 35 Ar
63 34 82 1
64 35 83 1
65 36 84 1
66 37 85 1
67 38 86 1
68 38 87 1
69 39 40 Ar
70 39 44 Ar
71 39 45 Ar
72 40 41 Ar
73 40 48 Ar
74 41 42 Ar
75 41 65 1
76 42 43 Ar
77 42 49 1
78 43 44 Ar
79 43 88 1
80 44 89 1
81 45 46 Ar
82 45 51 1
83 46 47 Ar
84 46 50 1
85 47 48 Ar
86 47 90 1
87 48 91 1
88 49 92 1
89 50 93 1
90 51 94 1
91 51 95 1
92 52 53 Ar
93 52 57 Ar
94 52 58 Ar
95 53 54 Ar
96 53 61 Ar
97 54 55 Ar
98 55 56 Ar
99 55 62 1
100 56 57 Ar
101 56 96 1
102 57 97 1
103 58 59 Ar
104 58 64 1
105 59 60 Ar
106 59 63 1
107 60 61 Ar
108 60 98 1
109 61 99 1
110 62 100 1
111 63 101 1
112 64 102 1
113 64 103 1
114 65 104 1
115 65 105 1
116 70 139 1
117 70 140 1
118 70 141 1
119 71 134 1
120 71 135 1
121 71 136 1
122 76 119 1
123 76 120 1

124 76 121 1
125 77 114 1
126 77 115 1
127 77 116 1
128 84 149 1
129 84 150 1
130 84 151 1
131 85 144 1
132 85 145 1
133 85 146 1
134 92 129 1
135 92 130 1
136 92 131 1
137 93 124 1
138 93 125 1
139 93 126 1
140 100 109 1
141 100 110 1
142 100 111 1
143 101 106 1
144 101 154 1
145 101 155 1
146 106 107 2
147 106 108 2
148 109 112 2
149 109 113 2
150 114 117 2
151 114 118 2
152 119 122 2
153 119 123 2
154 124 127 2
155 124 128 2
156 129 132 2
157 129 133 2
158 135 137 2
159 135 138 2
160 139 142 2
161 139 143 2
162 145 147 2
163 145 148 2
164 149 152 2
165 149 153 2
166 156 157 1
167 156 161 1
168 156 162 1
169 156 163 1
170 157 158 1
171 157 164 1
172 157 165 1
173 158 159 1
174 158 166 1
175 158 167 1
176 159 160 1
177 159 168 1
178 159 169 1
179 160 161 1
180 160 170 1
181 160 171 1
182 161 172 1
183 161 173 1
184 162 177 1
185 162 178 1
186 162 179 1
187 163 174 1
188 163 175 1
189 163 176 1
190 168 180 1
191 168 181 1
192 168 182 1
193 169 183 1
194 169 184 1
195 169 185 1

---

0 imaginary frequency
SCF Done: E(RB97D3) = -5562.93228536 A.U.

2D NOESY of 7^{2+}@PrS[5]$^{COO-}$

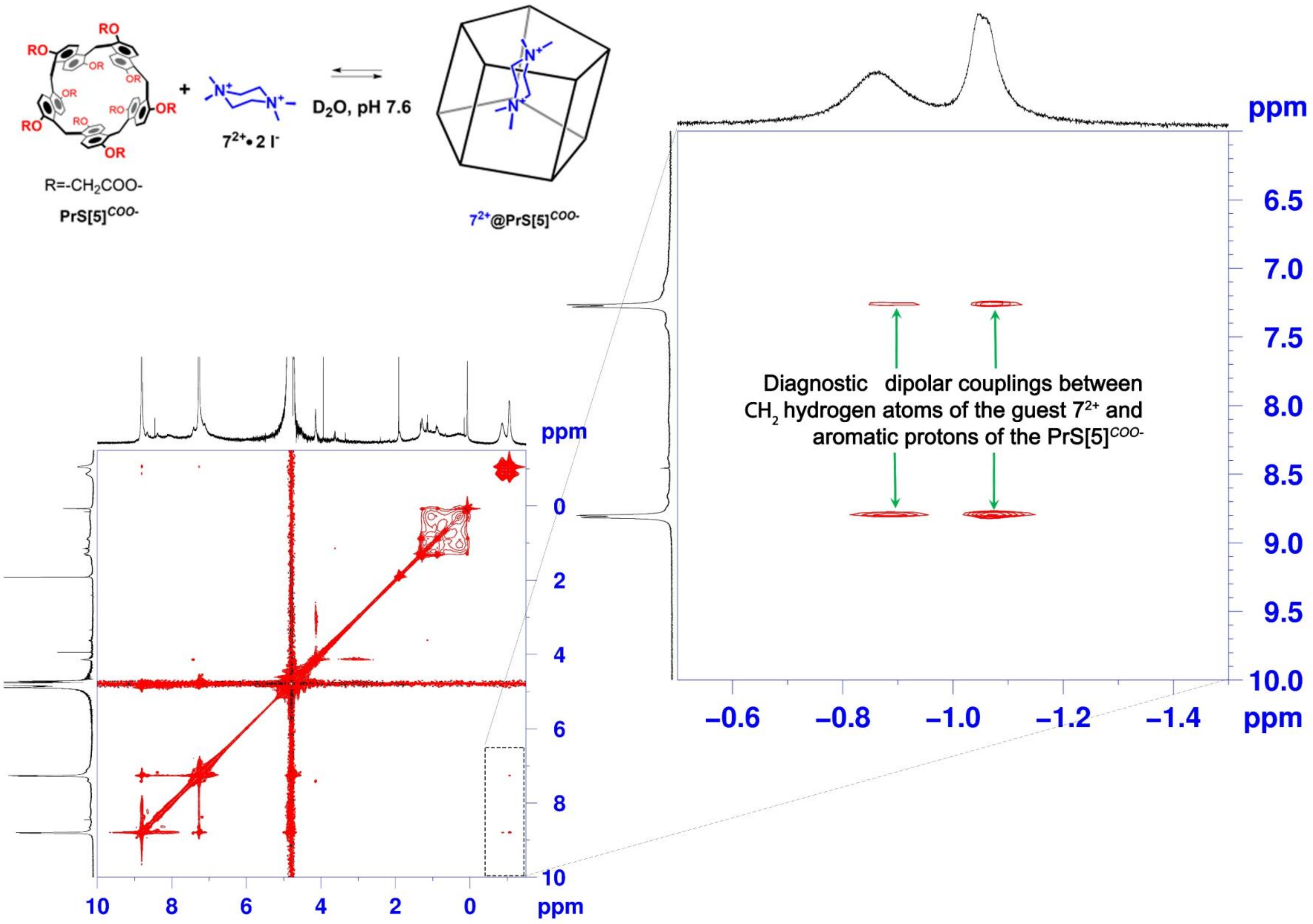

Figure S50. Significant portion of 2D-NOESY spectrum of **7^{2+}@PrS[5]$^{COO-}$** (600 MHz, buffered D_2O solution, pH 7.60, 298 K).

References:

1) Furniss, B. S.; Hannaford, A. J.; Smith, P. W. G.; Tatchell, A. R. Vogel's Textbook of Practical Organic Chemistry, 5th ed.; Pearson: Harlow, 1989 and references cited therein.

2) D. B. G. Williams, M. Lawton, Drying of organic solvents: quantitative evaluation of the efficiency of several desiccants. *J. Org. Chem*. **2010**, *75*, 8351−8354.

3) Fulmer, G. R.; Miller, A. J. M.; Sherden, N. H.; Gottlieb, H. E.; Nudelman, A.; Stoltz, B.M.; Bercaw, J.E.; Goldberg, K.I. Chemical Shifts of Trace Impurities: Common Laboratory Solvents, Organics, and Gases in Deuterated Solvents Relevant to the Organometallic Chemist. Organometallics 2010, 29, 2176–2179.

4) Del Regno, R.; Della Sala, P.; Picariello, D.; Talotta, C.; Spinella, A.; Neri, P.; Gaeta, C. per-Hydroxylated Prism[n]arenes: Supramolecularly Assisted Demethylation of Methoxy-Prism[5]arene. *Org. Lett.* **2021**, *21*, 8143–8146.

5) Sgarlata, C.; Zito, V.; Arena. G. Conditions for calibration of an isothermal titration calorimeter using chemical reactions. *Anal. Bioanal. Chem*. **2013**, *405*, 1085–1094.

6) Arena, G.; Gans, P.; Sgarlata, C. HypCal, a general-purpose computer program for the determination of standard reaction enthalpy and binding constant values by means of calorimetry. *Anal. Bioanal. Chem*. **2016**, *408*, 6413–6422.

7) Gaussian 16, Revision C.01, Frisch, M. J.; Trucks, G. W.; Schlegel, H. B.; Scuseria, G. E.; Robb, M. A.; Cheeseman, J. R.; Scalmani, G.; Barone, V.; Petersson, G. A.; Nakatsuji, H.; Li, M.; Caricato, M.; Marenich, A. V.; Bloino, J.; Janesko, B. G.; Gomperts, R.; Menucci, B.; Hratchian, H. P.; Ortiz, J. V.; Izmaylov, A. F.; Sonnenberg, J. L.; Williams-Young, D.; Ding, F.;Lipparini, F.; Egidi, F.; Goings, J.; Peng, B.; Petrone, A.; Henderson, T.; Ranasinghe, D.; Zakrzewski, V. G.; Gao, J.; Rega, N.; Zheng, G.; Liang, W.; Hada, M.; Ehara, M.; Toyota, K.; Fukuda, R.; Hasegawa, J.; Ishida, M.; Nakajima, T.; Honda, Y.; Kitao, O.; Nakaj, H.; Vreven, T.; Throssell, K.; Montgomery, J. A.; Peralta, Jr. J. E.; Ogliaro, F.; Bearpark, M. J.; Heyd, J. J.; Brothers, E. N.; Kudin, K. N.; Staroveroy, V. N.; Keith, T. A.; Kobayashi, R.; Normand, J.; Raghavachari, K.; Rendell, A. P.; Burant, J. C.; Iyengar, S. S.; Tomasi, J.; Cossi, M.; Millam, J. M.; Klene, M.; Adamo, C.; Cammi, R.; Ochterski, J. W.; Martin, R. L.; Morokuma, K.; Farkas, O.; Foresman, J. B.; Fox, D. J. Gaussian, Inc., Wallingford CT, 2019.
